# Simulating the council-specific impact of anti-malaria interventions: A tool to support malaria strategic planning in Tanzania

**DOI:** 10.1371/journal.pone.0228469

**Published:** 2020-02-19

**Authors:** Manuela Runge, Robert W. Snow, Fabrizio Molteni, Sumaiyya Thawer, Ally Mohamed, Renata Mandike, Emanuele Giorgi, Peter M. Macharia, Thomas A. Smith, Christian Lengeler, Emilie Pothin

**Affiliations:** 1 Swiss Tropical and Public Health Institute, Basel, Switzerland; 2 University of Basel, Basel, Switzerland; 3 Centre for Tropical Medicine and Global Health, Nuffield Department of Clinical Medicine, University of Oxford, Oxford, England, United Kingodm; 4 Population Health Unit, Kenya Medical Research Institute-Wellcome Trust Research Programme, Nairobi, Kenya; 5 National Malaria Control Programme (NMCP), Dar es Salaam, Tanzania; 6 CHICAS, Lancaster Medical School, Lancaster University, Lancaster, England, United Kingodm; 7 Clinton Health Access Initiative, Boston, Massachusetts, United States of America; Imperial College London, UNITED KINGDOM

## Abstract

**Introduction:**

The decision-making process for malaria control and elimination strategies has become more challenging. Interventions need to be targeted at council level to allow for changing malaria epidemiology and an increase in the number of possible interventions. Models of malaria dynamics can support this process by simulating potential impacts of multiple interventions in different settings and determining appropriate packages of interventions for meeting specific expected targets.

**Methods:**

The *OpenMalaria* model of malaria dynamics was calibrated for all 184 councils in mainland Tanzania using data from malaria indicator surveys, school parasitaemia surveys, entomological surveillance, and vector control deployment data. The simulations were run for different transmission intensities per region and five interventions, currently or potentially included in the National Malaria Strategic Plan, individually and in combination. The simulated prevalences were fitted to council specific prevalences derived from geostatistical models to obtain council specific predictions of the prevalence and number of cases between 2017 and 2020. The predictions were used to evaluate *in silico* the feasibility of the national target of reaching a prevalence of below 1% by 2020, and to suggest alternative intervention stratifications for the country.

**Results:**

The historical prevalence trend was fitted for each council with an agreement of 87% in 2016 (95%CI: 0.84–0.90) and an agreement of 90% for the historical trend (2003–2016) (95%CI: 0.87–0.93) The current national malaria strategy was expected to reduce the malaria prevalence between 2016 and 2020 on average by 23.8% (95% CI: 19.7%-27.9%) if current case management levels were maintained, and by 52.1% (95% CI: 48.8%-55.3%) if the case management were improved. Insecticide treated nets and case management were the most cost-effective interventions, expected to reduce the prevalence by 25.0% (95% CI: 19.7%-30.2) and to avert 37 million cases between 2017 and 2020. Mass drug administration was included in most councils in the stratification selected for meeting the national target at minimal costs, expected to reduce the prevalence by 77.5% (95%CI: 70.5%-84.5%) and to avert 102 million cases, with almost twice higher costs than those of the current national strategy. In summary, the model suggested that current interventions are not sufficient to reach the national aim of a prevalence of less than 1% by 2020 and a revised strategic plan needs to consider additional, more effective interventions, especially in high transmission areas and that the targets need to be revisited.

**Conclusion:**

The methodology reported here is based on intensive interactions with the NMCP and provides a helpful tool for assessing the feasibility of country specific targets and for determining which intervention stratifications at sub-national level will have most impact. This country-led application could support strategic planning of malaria control in many other malaria endemic countries.

## Introduction

In the last decade, the malaria burden has substantially decreased globally. However, in recent years the decline in malaria burden has stagnated, with 435,000 estimated deaths due to malaria in 2017, compared to 451,000 in 2016, and only marginal improvements in insecticide treated bed net (ITN) coverage since 2015 [1]. Mosquito resistance to insecticides used for vector control, parasite resistance against antimalarials, weak case management systems as well as waning immunity and insufficient funding are current challenges for achieving more ambitious malaria control and elimination goals [1,2]. Intensified efforts are needed, especially in high burden countries in Sub-Saharan Africa, and national malaria control strategies need to be adapted to local settings and challenges [1,2]. At the same time, the decision-making process for selecting appropriate malaria control and elimination strategies has become more challenging because of changing malaria epidemiology. As risk levels have decreased significantly in many areas but not in others, there is now a need to target interventions at sub-national level, in order to prioritise and efficiently allocate resources [3]. At the same time, there is a need to consider new interventions, and more generally, the cost and impact of all interventions as well as their combinations [2,4]. The combination of available empirical data with mathematical models is a powerful approach for providing and additional layer of information for strategic planning by predicting the impact of interventions given local knowledge [5].

### Malaria in Tanzania

Tanzania is one of the highest malaria burden countries in the world [6], but transmission intensity (predominantly of *Plasmodium falciparum*) is very heterogeneous, with areas of low prevalence in the middle part of the country, from north-east to south-west, and areas with high prevalence around the Lake Zone and in the South-East. The main malaria vectors are *An*. *funestus s*.*l*., *An*. *gambiae s*.*s* and *An*. *arabiensis* [7]. Scale-up of malaria control interventions after 2000 started relatively early in Tanzania, as described in the national epidemiological profile [8]. In brief, the Tanzanian National Long-Lasting Insecticide Nets Voucher Scheme (TNVS) was introduced in 2004 [9], artemisinin combination therapy (ACT) became first-line antimalarial therapy in 2006, and indoor residual spraying (IRS) was introduced in 2007 in selected councils in the Lake Zone [8]. ITNs were distributed in three mass campaigns in the whole country between 2009 and 2011 [8,10,11] and again in 2016, while in 2013 ITN distribution in schools started in selected regions (Mtwara, Ruvuma, Lindi) [12,13]. These campaigns were recently scaled up to include Geita and Kagera Regions. According to recent Demographic Health and Malaria Indicator Surveys, the national malaria prevalence in children less than five years had been reduced from 18% in 2008 to 10% in 2012, it then increased to 14% in 2016 before decreasing to 5% in 2017 [14–17]. The achievement of the past years are challenged by insufficient coverage rates in all interventions, resistance against pyrethroids and by changing vector occurrence and their contribution to transmission [18–21]. These factors require an intensified approach to control, with higher intensity of implementation, new products to deal with resistance, and finally also new interventions to deal with residual transmission. Since available resources are unlikely to increase, it is now more important than ever to define appropriate mixes of interventions according to the epidemiological situation, as mentioned above [1].

### National Malaria Strategic Plan 2015–2020

The National Malaria Strategic Plan 2015–2020 (‘current NMSP’) envisages ITN distribution in all councils, IRS in some councils around the Lake Zone, strategies to improve case management (CM) everywhere [22] and larval source management (LSM), in particular, larviciding in urban areas. The current NMSP aimed at halving the malaria prevalence from 10% in 2012 to 5% in 2015 to less than 1% in 2020 and to achieve at least 85% access to ITNs. In line with World Health Organisation (WHO) recommendations, Tanzania is poised to define ways to maximise the future disease impact, reduce inefficiencies and create, for the first time, a platform to sub-nationally target resources and monitor progress [6].

### Modelling to support strategic planning

Many malaria models exist, and a detailed description of existing models and classifications has been published elsewhere [23–25]. Mathematical modelling is used to simulate the impact of interventions to explore and assess relationships among malaria transmission parameters [26–28] or simulate the impact of interventions for defined geographical areas at different spatial resolutions [29–34]. Geographical and temporal predictions are useful for decision-making processes at country level or global level (e.g. WHO) [2,35,36]. Mathematical models have been applied in various studies at high resolution in all sub-Saharan African (SSA) countries [29,30,37,38], or more local levels, for example in Kenya [39–43], Nigeria [31,32,44], South Africa [45,46], Ghana [41,47,48], Uganda [41], Mozambique [49], and Tanzania [41], and modelling is also being done in Asia [50–52]. Previous country specific model predictions were either generalised based on archetypical settings at regional level (admin 1) [31], at 5x5 km^2^ level [29], or applied for a specific sub-area of the country [33,53–55]. But this process has never been applied for a whole country at an administrative level useful for sub-national strategic planning or for resource allocation (e.g. council for financing) and based on country specific data as well as the history of malaria in each setting.

The NMCP in Tanzania is in the process of re-evaluating its national malaria strategic plan, moving towards interventions targeted at council level. Modelling has been used previously as part of the strategic planning for the NMCP [41], but not for all councils. This paper describes the developed modelling calibration and analysis workflow for all 184 councils in Tanzania [56]. The objectives were: (1) to present how this methodology can simulate the current epidemiology of malaria in each council, (2) to present the council-specific predicted impact of different anti-malaria interventions, (3) to assess *in silico* whether the 2015–2020 NMSP objectives to reduce malaria prevalence below 1% by 2020 is feasible with currently available interventions assuming malaria situation is known until 2016, and (4) to suggest an allocation of interventions per council optimised for cost-effectiveness or one that would reach the 2015–2020 NMSP target at minimised costs.

## Materials and methods

### Data collation

An assemblage of malaria prevalence data built up from the Mapping Malaria Risk in Africa (MARA) project database [57] and updated in 2013 and 2018, provided a national geo-coded repository of malaria survey data on mainland Tanzania [58]. These data have been used within a model based geo-statistical framework to provide properties of malaria risk, as measured by the parasite rate in children aged 2–10 years (*Pf*PR_2-10_), across the entire country for every year between 1990–2017. The geostatistical model did not include covariates, following the same approach as applied in Somalia [59], Kenya [60] and Malawi [61] (methods provided in the S1 File)). The high resolution prevalence estimates were aggregated at council level for the years 2003 to 2016. Intervention coverage estimates were obtained from malaria indicator surveys [15,16,62], and in addition, average insecticide treated net coverage estimates were obtained from the Malaria Atlas Project (MAP) [63,64]. MAP uses geo-spatial modelling to generate high-resolution predictions for different malaria outcomes using household survey data, surveillance and research data. Information on vector occurrence and sporozoites rates were obtained from national entomological surveillance surveys carried out in 2016, entomological research studies conducted in Tanzania, previous model parameterisations [26,65,66], as well as expert discussion with local entomologists. Commodities data for net distributions and indoor residual spraying coverage per council per months were also extracted from NMCP records. The ITN distribution data was compared to reported coverage from Malaria Indicator Surveys (MIS) and model-predicted estimates from MAP (S2 File). Population estimates were obtained per council from the national census in 2012 with forward projections based on an assumed constant growth rate per council [67]. The collated data was combined into a comprehensive database including regional and council estimates if available.

### Simulation models

The *OpenMalaria* modelling platform was used to simulate the impact of different intervention strategies at council level. *OpenMalaria* is a stochastic simulator of malaria epidemiology and control developed at the Swiss Tropical and Public Health Institute (Swiss TPH) [68]. It includes individual-based models of the dynamics of malaria in humans combined with a population model of malaria vectors. Biological parameter and intervention efficacy were calibrated to field data and not changed. Country specific were obtained from the collated data, including intervention deployment and coverage, case management levels, as well as vector composition, resistance and biting behaviour. Details on *OpenMalaria* development and parameterisations are available in previous literature [26,66,68–70] and online [71]. A comprehensive comparison of the model with other models was published by Smith et al. [23].

#### Simulation design

*OpenMalaria* version 32, model variant *R0068* [72] was used. The simulations ran for a population of 10,000 individuals, assuming an importation rate of five infections per 1,000 population per year [73,74]. The parasite detection limit was set to 200 parasites per microliter (p/*μ*L), corresponding to the detection limit for standard microscopy procedures as well as rapid diagnostic tests [75]. Both diagnostics were used in generating data underlying the study but the differences between model estimates based on the different diagnostics would be small in relation to the size of effects needed to change the conclusions of this study. The simulations were run per region and fitted to prevalence per councils after the simulations. The assumption of similar parameters among councils within regions was shown in the available data for seasonality and historical ITN coverage (S2 File), and necessary for parameters for which only sparse data (i.e. vector bionomics), or no representative data was available at council level (i.e. case management). This substantially reduced the number of simulations to run.

**Fig 1:**
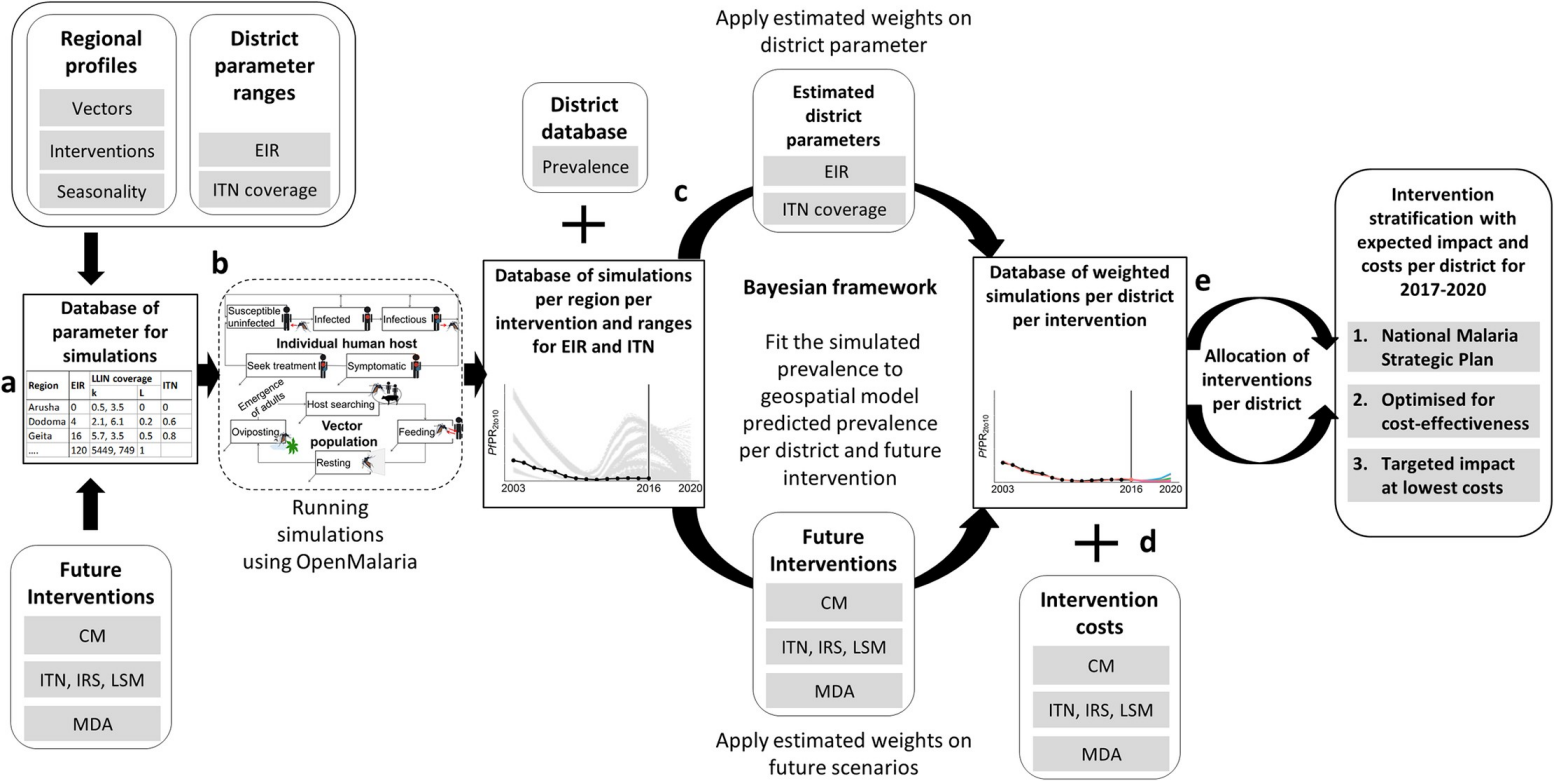
Schematic visualisation of the calibration and analysis methodology. **a**) Illustrates model parameterisation, including selection of parameters derived from data per region, base transmission parameter previously determined from field data, and future intervention scenarios. **b**) The regional transmission models were run using *OpenMalaria* resulting in a database of simulation outcomes. **c**) The simulated prevalence per region, including several levels of pre-intervention EIR and ITN coverage and future intervention scenarios, were fitted to the to geospatial model-predicted prevalence per council, using a Bayesian framework. In this process weights were generated for each simulated historical scenario and each council and applied on the future intervention scenarios, resulting in a reduced database of weighted simulations per council per intervention. **d**) Intervention unit costs from literature were attached to the database, and the costs for every single intervention per council were calculated. **e**) Allocation of intervention combinations per council according to the current NMSP or by either optimising for cost-effectiveness or by conditioning the simulated prevalence to meet the national target (*Pf*PR <1% by 2020).

Separate model parameterisations were used for each of the 184 councils and 26 regions of Tanzania (Fig 1A). Each simulation was initiated at an approximate endemic steady state determined by vector bionomics parameters (Table 1). Simulations then ran for a further pre-intervention phase of 46 years at this steady state; a historical intervention phase of 13 years starting in 2003 (simulation time), and a future intervention phase from 2017 to 2020.

**Table 1:** List of parameters assigned at regional level for 2003–2016.

Category	Parameters
Vector bionomics	Mosquito population characterised by biting and resting behaviours Contribution of each mosquito population to transmission
	Proportion of vectors affected by indoor intervention
	Seasonality
Case management	Proportion of symptomatic cases effectively treated
Insecticide treated bed nets	Coverage (assumption: coverage = effective usage)
	Deployment scheme
	Frequency
Indoor residual spraying	Coverage
	Timing
	Frequency

During the historical intervention phase, simulated levels of coverage of both vector control and effective case management (Table 1) changed annually based on values obtained by averaging the local field data given for each region. A full factorial experiment of simulations was run with ranges of values for varying pre-intervention EIR values in 2003 and effective ITN coverage between 2012 and 2016 per region (Table 2). Council-specific simulations were obtained by estimating the levels of those two parameters council by assigning weights based on simulated and geospatial model predicted prevalence as reference for the historical trend 2003 to 2016. The methodology to assign an estimated weight for each simulation in order to fit council-specific data is described in S2 File.

**Table 2:** Description of the full factorial simulation experiment for each region.*).

Region parameters	Council parameters to fit historical prevalence trend			Future intervention scenarios and coverages					
Region	EIR	ITN coverage***		CM	ITN		IRS	Additional	
		ITN decay 2011 (k,L)	ITN distribution coverage 2012–2016						
Arusha	0	0.5, 3.5	0	Region specific	0		0	None	
Daressalaam	4	2.1, 6.1	0.2	0.85	MRC (0.8)		0.85	LSM (0.6)	
Dodoma	16	5.7, 3.5	0.5		Continuous (0.7)			MDA (0.8)	
Geita	54	6, 5.3	0.8						
….	120	5449, 749	0.95						
[26 regions]	550**								

*) In total 23940 scenarios were simulated, including, 36 future scenarios for all 26 regions and 25 levels for the council parameters baseline EIR and ITN coverage in 23 regions with 30 levels in the other 3 regions.

**) The regions receiving nets through schools (SNP) had higher prevalence estimates in 2003 and six instead of five pre-intervention EIR levels were simulated.

***) ITN coverage for regions receiving nets through schools between 2012–2016 (Mtwara, Lindi and Ruvuma) and ITN decay for regions that had a mass campaign in 2012 and no large scale ITN distribution until 2015/16.

The future intervention phase considered five interventions simulated individually and in combination, leading to a total of 36 scenarios for future strategies for the 2017–2020 period. These, combined with factors representing the historical intervention phase led to a total of 900 simulations per region (Table 2). The future interventions considered were: effective treatment coverage (improved case management—CM), long-lasting insecticidal nets (ITNs), wither as mass campaign (ITN MRC), or annual distribution targeting schoolchildren (“ITN continuous”), indoor residual spraying (IRS), larviciding (larval source management—LSM), and mass drug administration (MDA). The interventions were selected based on the core interventions (CM, ITN, and IRS) included in the national malaria strategic plan in Tanzania and potential additional interventions (MDA, LSM). Details on the simulated intervention effectiveness and deployment are provided in the S2 File.

#### Fitting and future projections per council

The *OpenMalaria* simulations resulted in a large database with predicted malaria prevalences and number of cases for the years 2003 to 2020 per region, per future intervention scenario, for five different levels of pre-intervention EIR until intervention scale-up starting from 2003, and for five ITN coverage levels determining the population protected by ITNs between 2012 and 2016 (Table 2). The varying ITN coverage levels at council level between the mass campaigns in 2011 and 2016 were obtained by applying a standard model for net attrition to the coverage achieved by the net distribution in 2011 for 23 regions. In the three other regions (Mtwara, Lindi, and Ruvuma) nets were distributed yearly via a school net program, and the annual ITN coverage level was varied between 2012 and 2016 [13]. Each council was treated as a weighted average of the 25 simulations per region, based on varying council parameter (5 EIR levels x 5 ITN coverage levels). The weights were estimated using a Bayesian MCMC model that compared the weighted average prevalence with the values extracted from the geostatistical model (S1 File). This provided weighted averages of simulation outcomes per council over time (Fig 1C). The simulation outcomes were processed and fitted using R and JAGS software [76,77]. The median estimates of the posterior distribution resulting from the model calibration were used for the analysis. Lin’s concordance correlation coefficients (CCC) were calculated to assess fitting performance between geospatial model predicted and mathematical predicted prevalence estimates [78].

#### Approximation of costs

For each intervention, assumptions on unit costs in USD per population were extracted from the literature and agreed upon based on local expert knowledge on intervention deployments in Tanzania. Total costs were calculated for the years 2017 to 2020, for each intervention and their combinations for the total population per council (Fig 1D). A full description of the process is provided in the supplement (S3 File).

### Intervention stratification

The council-specific simulation database included predicted yearly prevalence and cases and was combined with the cost for each intervention in order to explore impact and cost of three alternative intervention allocations per council (referred to as ‘strategies’) (Fig 1E).

#### Strategy 1: Allocation of interventions according to the NMSP 2015–2020

The current NMSP 2015–2020 described intervention packages at the regional level. The whole country is expected to receive ITNs distributed through different channels and combined with IRS in selected councils in the Lake Zone. Additionally, LSM is expected to be implemented in urban councils (city councils and municipalities). The current NMSP was simulated with considering vector control only or in combination with improved case management in all councils.

#### Strategy 2: Allocation of interventions optimised for cost-effectiveness

The interventions were optimised for cost-effectiveness at council level, by iteratively minimising the incremental cost-effectiveness ratio (ICER) among ‘admissible’ interventions (interventions that remain after removing interventions that are more expensive but less effective than the least costly intervention [79]). Impact was defined by the cumulative cases averted compared to the counterfactual scenario, and costs defined as the total costs per capita from 2017 to 2020 per council. The ICER calculation followed the approach used by Otieno *et al*. [40] based on Okosun *et al*. [80].

#### Strategy 3: Allocation of interventions according to the selection of cost-minimised interventions that lead to the NSMP target (National prevalence below 1% in 2020)

The second algorithm for intervention allocation per council based on simulated impact and costs consisted of selecting the intervention scenario with the minimum cost and expected prevalence of less than 1% per council by 2020.

### Presentation of results

The prevalence was categorised into six groups (*Pf*PR_2-10_ <1%, 1–5%, 5–10%, 10–25%, 25–50% and >50%) according to the adapted traditional endemicity classes as described in the epidemiological profile of Tanzania [8]. The simulated total number of cases included both, the total number of uncomplicated and severe cases, with the incidence calculated as total cases per 1000 population. Two scenarios were defined as a comparator for the calculation of relative reductions per future intervention scenario: 1) the situation in 2016 before deployment of future interventions, referred to as “baseline”, and 2) the future scenario with discontinuation of vector control referred to as “counterfactual”. The relative prevalence reductions were calculated by comparing each future intervention to the baseline scenario, and the number of cases averted was calculated in comparison to the counterfactual. At national level, an un-weighted mean of the council estimates was calculated with confidence intervals based on the variation between councils. Maps were generated using QGIS [81] and R [76].

## Results

### Historical trend of malaria–geostatistical model predictions

The national prediction from the geostatistical model for malaria prevalence in 2003 was 29.9%, ranging from 0.96% to 71.5% among councils and in 2016 15.7%, ranging from 0.006% to 52.9% among councils. The average decline in prevalence between those years was 56.6%, ranging from -51.6% to 99.4% among councils.

### Fitting performance

**Fig 2:**
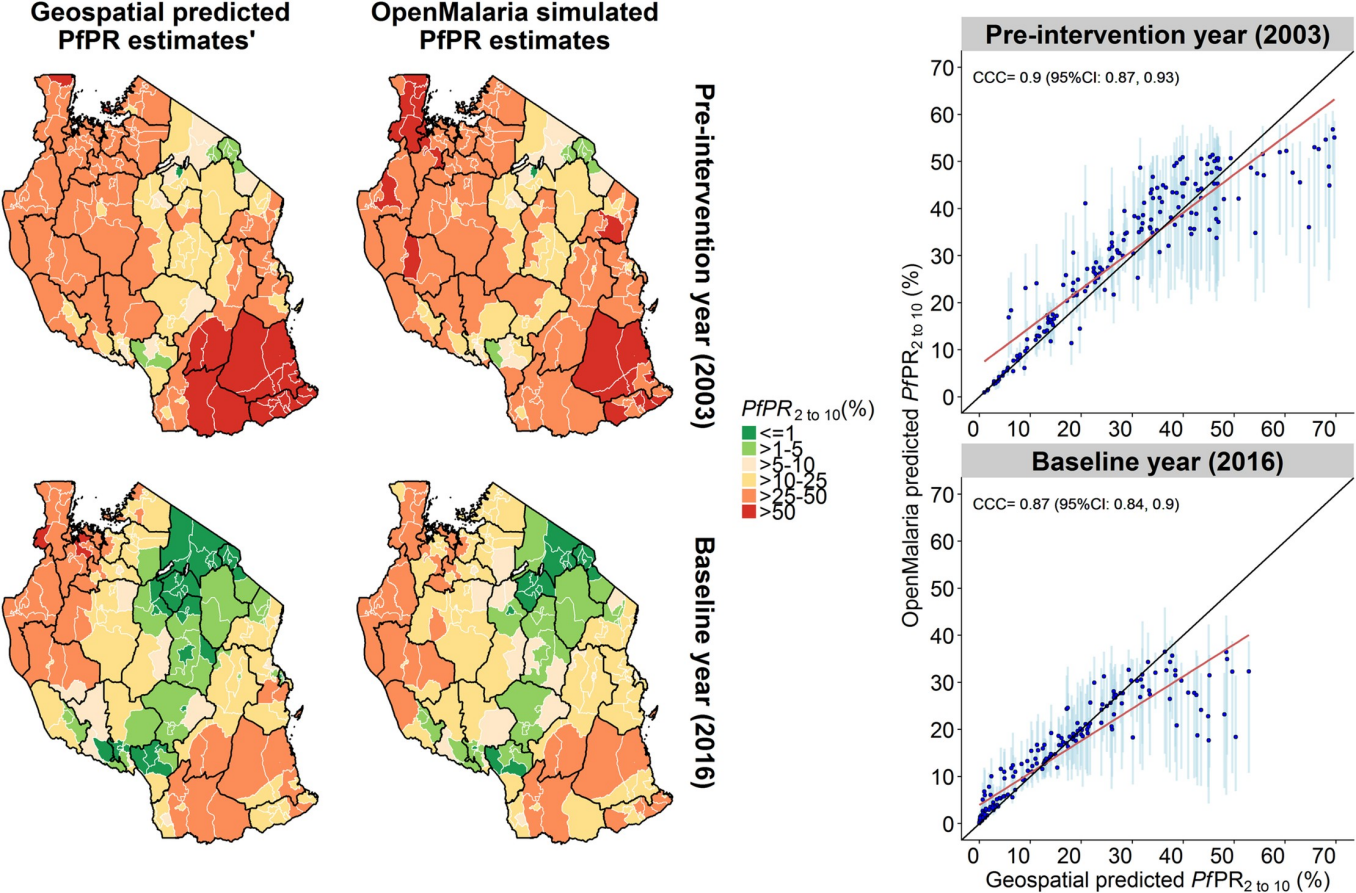
Fitting performance for simulated prevalence per council. The figure shows the predicted prevalence (median) and the geo-statistical model prevalence (mean). The pre-intervention (2003) and baseline year (2016) are the most relevant historical time points since the prevalence before the deployment of interventions determines the level of possible rebound, and the prevalence in the baseline year is used as a comparator for assessing the relative impact of future interventions. The scatter plots (right) shows the respective prevalence estimates (points), with the regression line (red line), and perfect correspondence line (black line). CCC = Lin’s concordance correlation coefficients [78].

The typical geographical pattern of malaria prevalence in Tanzania, with the highest prevalence around the Lake Zone as well as in the South-Eastern Zone and low prevalence in the “middle corridor”, was well reproduced with *OpenMalaria* simulations. The concordance correlation coefficient for the whole historical trend between geostatistical model predictions and *OpenMalaria* predictions was on average 0.83 (95%CI: 0.82, 0.84), 0.91 (95%CI: 0.89, 0.93) for the year 2003, and 0.93 (95%CI: 0.91, 0.94) for the year 2016. The mean deviance between observed and simulated prevalence in 2003 was 5.8%, and in 2016–0. 8% (Fig 2). In the baseline year 2016, the fitting was best in twelve regions (CCC > 0.80), moderate in nine regions (CCC > = 0.6), poor in four regions (CCC < 0.6), and very poor in Geita region (CCC < 0) (S4 File). Very low or very high prevalence was not fitting the historical trends as well.

### Council specific parameter estimates

The estimated mean pre-intervention EIR (2003) was 94 infectious bites per person per annum (ibpa) (95%CI: 76–111 ibpa), ranging from 0.7, in Siha Council (Kilimanjaro Region) to 507 ibpa in Tandahimba (Mtwara Region) (IQR: 26–96 ibpa). The estimated pre-intervention EIRs are shown in the S4 File. The geographical pattern of the estimated pre-intervention EIR corresponds to the frequently described pattern of malaria in Tanzania, with low malaria transmission in the dry and hot ‘middle corridor’ and higher transmission around the Lake Zone and the South-Eastern areas. The estimated mean ITN coverage between 2012 and 2016 for councils in the school net program was 56% (95%CI: 49% - 65%), ranging from 16% in Namtumbo (Ruvuma Region) to 79% in Nachingwea (Lindi Region) (IQR: 41% - 73%).

### Historical trend of malaria–mathematical model predictions

The simulated national prevalence was 30.8% (95%CI: 28.6% - 33.1%) in 2003 and 14.7% (95%CI: 13.1% - 16.2%) in 2016, dropping by 59.9% (95%CI: 57.1% - 62.8%). In 2003, the simulated national incidence was 752 cases per 1000 population (95%CI: 727–779) with a reduction of 25.6% (95%CI: 20.3% - 30.1%) between 2003 and 2016.

### Predicted impact in 2020 of single interventions and combinations

**Fig 3:**
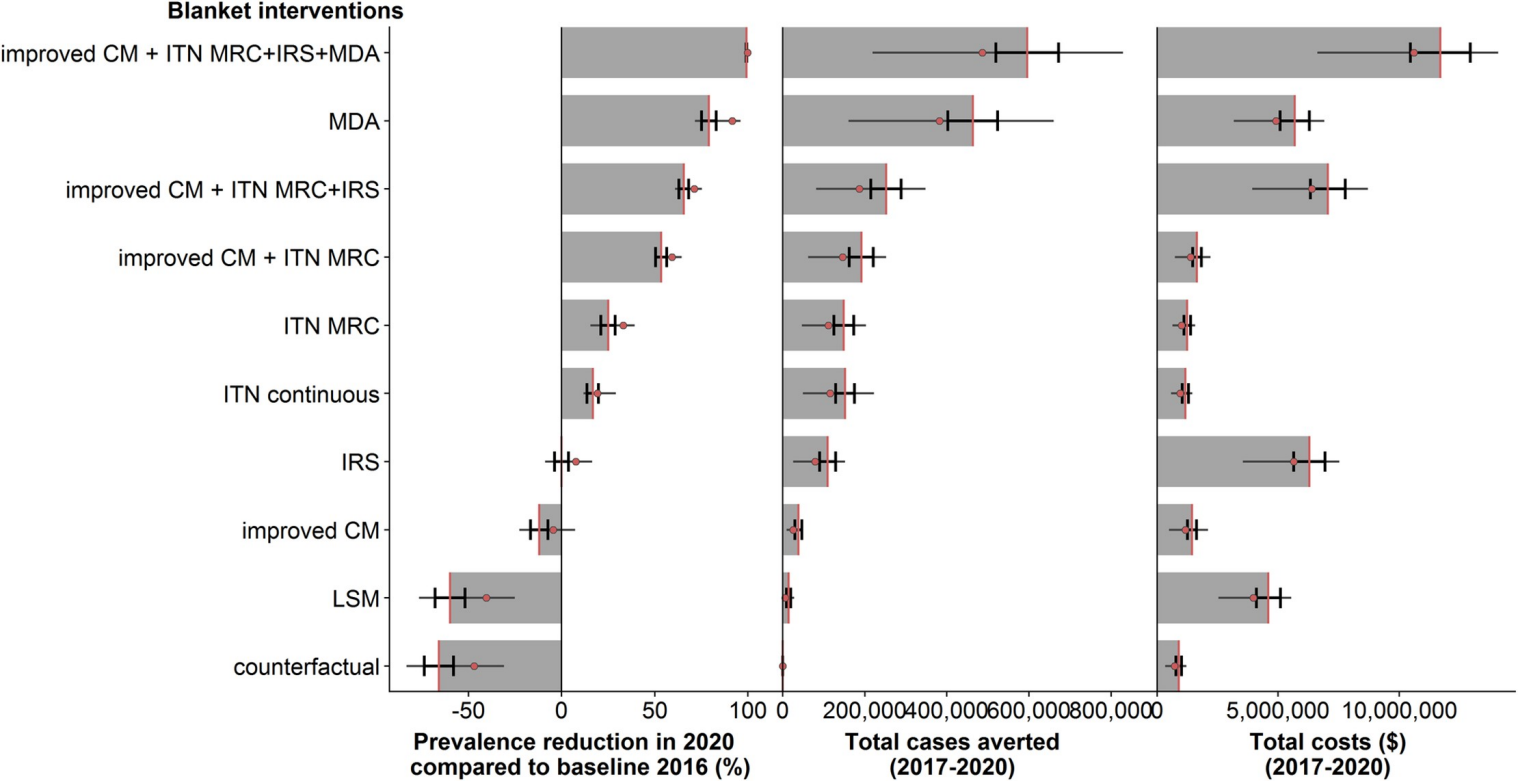
Simulated prevalence, cases averted and costs for single and incremental intervention scenarios from 2017–2020. The interventions scenarios shown visualise blanket distributions, with the same intervention scenario for all councils. The vertical lines indicate the confidence interval around the mean (red line), the red point the median, and the horizontal lines the 50% interquartile range between councils.

Without additional interventions (counterfactual scenario), the national prevalence was expected to increase by 65.7% (95%CI: 58.0% - 73.6%) in 2020 compared to the baseline in 2016. A scenario with an improvement in case management, assuming 85% of the cases would be effectively treated, and discontinued vector control predicted an increase in prevalence by 12.0% (95%CI: 7.3% - 16.7%, median = 4.5%), but the increase was expected to be lower than the increase of the counterfactual scenario (31.0% increase, 95%CI: 29.3% - 32.7%). The simulated ITN mass campaign in 2019, with 80% coverage, was simulated with a national prevalence reduction of 25.0% (95%CI: 21.1% - 28.8%). ITN distributed annually, maintaining 70% coverage, were expected to lead to a prevalence reduction of 16.7% (95%CI: 13.6% - 19.8%). When IRS was implemented, the simulated national prevalence was roughly maintained (95%CI: -3.9–3.6). The single deployment of larviciding at 60% for three months during the dry season was on average not expected to decrease the prevalence in the absence of other interventions (60.0% increase, 95%CI: 51.8% - 67.9%). MDA was expected to result in the highest impact with a prevalence reduction of 79.0% (95%CI: 75.1%-83.0%). The combination of improved case management and a ITN mass campaign resulted in an expected prevalence reduction of 53.4% (95%CI: 50.4% - 56.5%), additional IRS deployment led to a further reduction of 65.5% (95%CI: 63.0% - 68.1%), while additional MDA deployment let to an even further reduction of 99.3% (95%CI: 99.1%-99.5%). Overall, the higher the prevalence reduction, the higher the number of cases averted, whereas the costs were highest for interventions including MDA, IRS and LSM (blanket intervention for four years, not considering treatment savings) (Fig 3).

**Fig 4:**
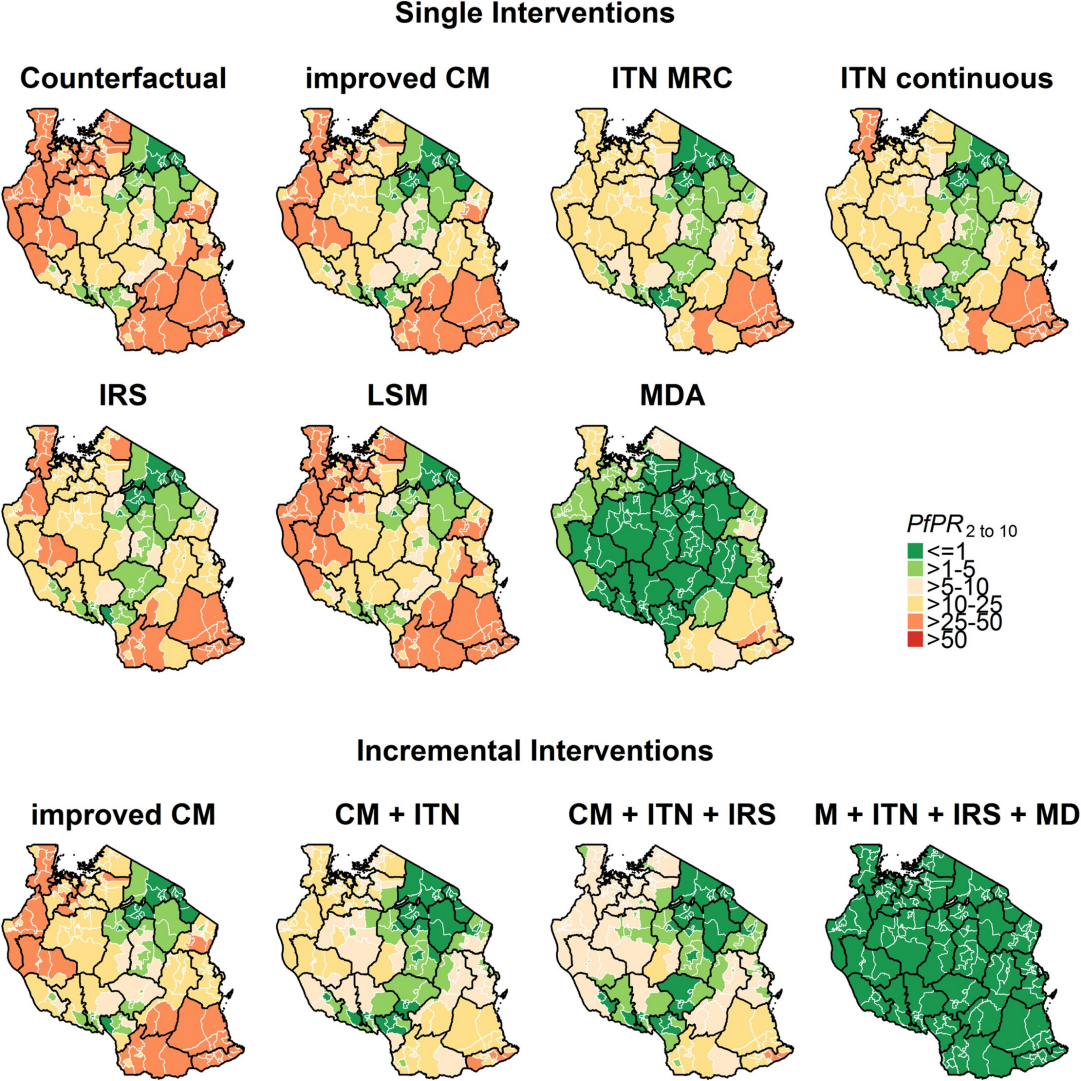
Simulated prevalence in 2020 for single and incremental intervention scenarios between 2017 and 2020. Each map shows the 2020 council-specific predicted prevalence for the age-group 2–10 years, resulting from the scale-up of every single intervention, or selected combinations of interventions. The interventions scenarios shown visualise blanket distributions, with the same intervention scenario for all councils.

The simulated outcomes showed high heterogeneity between councils, as shown for the prevalence per single intervention in Fig 4.

### Predicted impact in 2020 of intervention stratifications

#### Strategy 1: Allocation of interventions according to the NMSP 2015–2020

**Fig 5:**
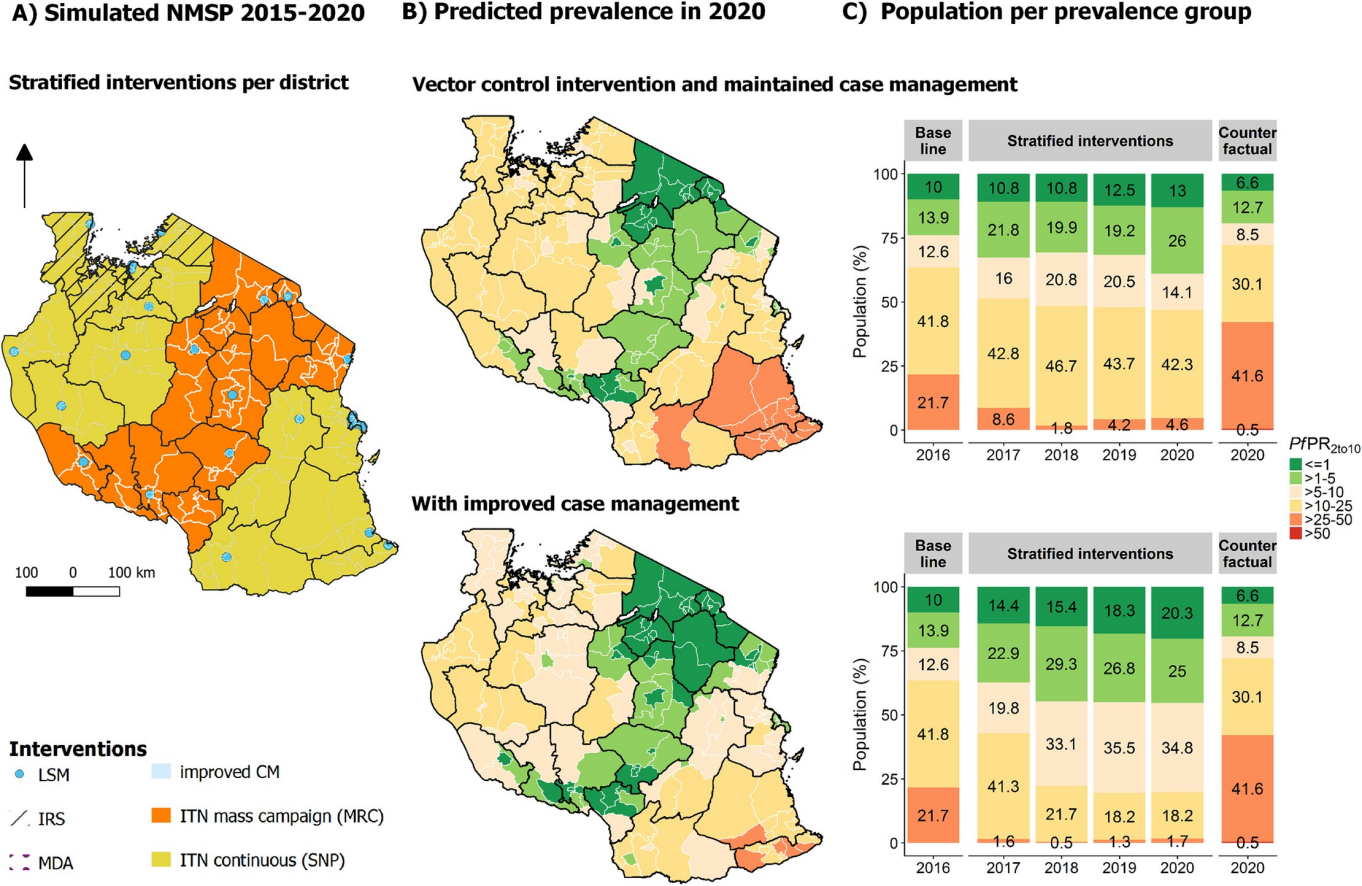
Strategy based on the NMSP 2015–2020 and simulated impact (Strategy 1). (A) Allocation of interventions per council, showing vector control interventions only. (B) Predicted prevalence per council in 2020 shown in the map and number of councils per simulated endemic group by year for 2017–2020 shown in the bar charts.

The NMSP envisaged ITN mass campaigns in 79 councils, ITN continuous distribution through schools in 105 councils, IRS in 24 councils and LSM in 25 councils (Fig 4). The simulated malaria prevalence in 2020 was 7.4% (95%CI: 6.4% - 8.4%), with improved case management levels, and 11.2% (95%CI: 9.9% - 12.6%) with maintained case management levels (Fig 5). This corresponds to a reduction of 23.8% (95% CI: 19.7%-27.9%) between 2016 and 2020 if current case management levels were maintained, and of 52.1% (95% CI: 48.8%-55.3%) if the case management were improved. The strategy was further expected to avert around 39–46 million cases between 2017 and 2020 while saving around $42–101 millions in treatment costs, with the ranges depending on improvement in case management. The total costs for this strategy (ITNs, treatments, IRS and LSM) were estimated at around $536–606 Mio for 2017–2020 ($16.7–15.2 per case averted, $412 Mio excluding treatment costs). While this impact is impressive, these results suggest that the current plan would not be sufficient to achieve the stated target to obtain a national prevalence of less than 1% by 2020.

#### Strategy 2: Allocation of interventions optimised for cost-effectiveness

**Fig 6:**
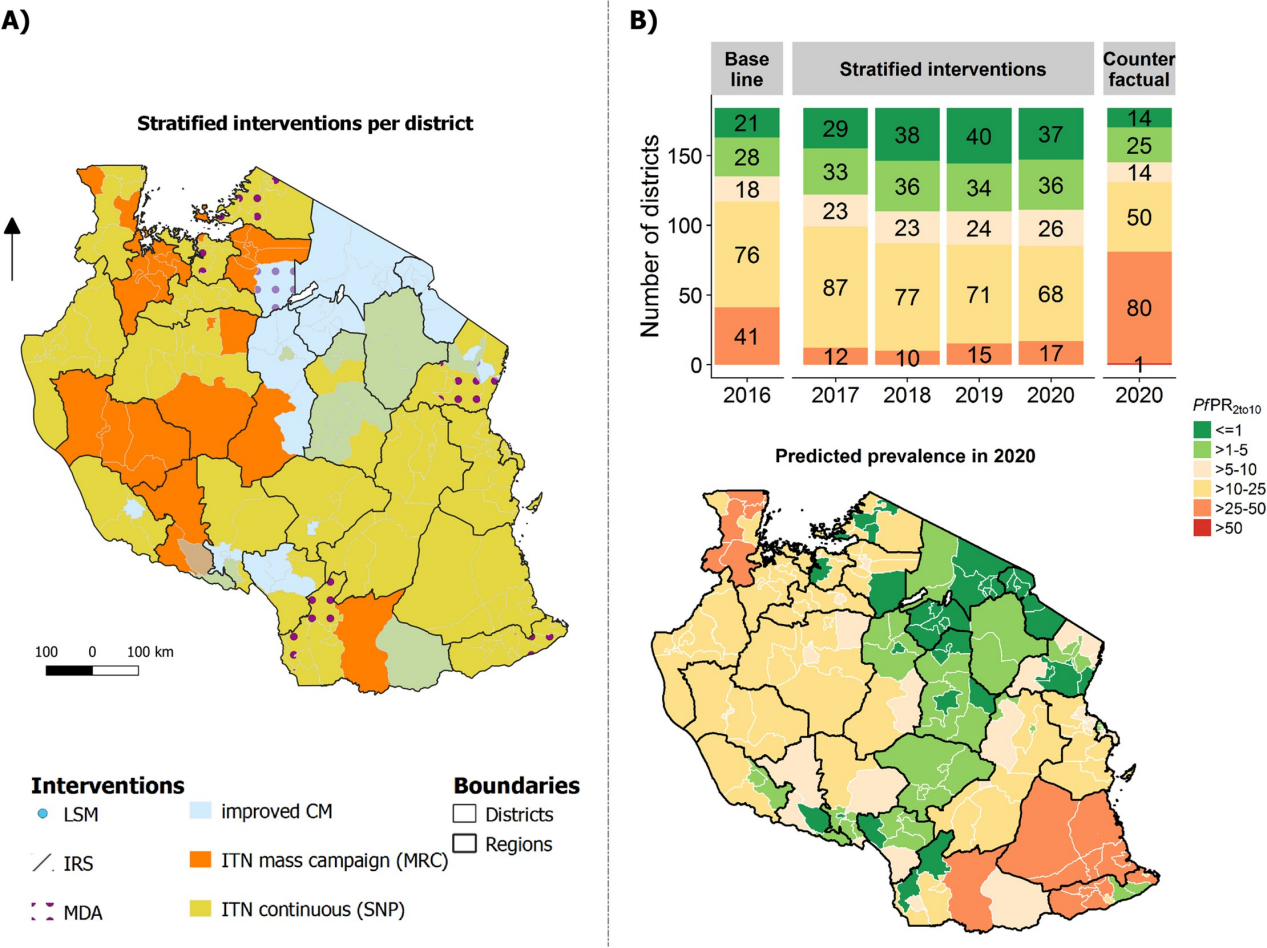
Strategy optimised for cost-effectiveness and simulated impact (Strategy 2). (A) Allocation of interventions per council. (B) Predicted prevalence per council in 2020 shown in the map and number of councils per simulated endemic group for 2017–2020 shown in the bar chart.

In most councils, the distribution of ITNs (mainly continuous), which is assumed to lead to a high level of use, would be the most cost-effective intervention. The intervention allocation optimised for cost-effectiveness entailed distribution of ITNs in all but very few councils (149 councils), together with an increase in case management coverage (50 councils) and a mass drug campaign (13 councils). The optimal intervention allocation per councils, its expected impact on prevalence and resulting percentage of population living per endemic category is shown in Fig 6. It was simulated that the national malaria prevalence in 2020 would be 10.5% (95%CI: 9.1% - 11.9%), corresponding to a prevalence reduction of 25.0% (95% CI: 19.7%-30.2). This strategy was further predicted to avert around 37 million cases between 2017 and 2020 while saving around $54 million in treatment costs. The total costs for this strategy were estimated at around $254 Mio ($6.90 per case averted), and $131 million excluding treatment costs for 2017–2020. MDA was selected in 23 councils (12.5%), yet contributed 50% of the estimated costs.

#### Strategy 3: Allocation of interventions according to the selection of cost-minimised interventions that lead to the NSMP target

**Fig 7:**
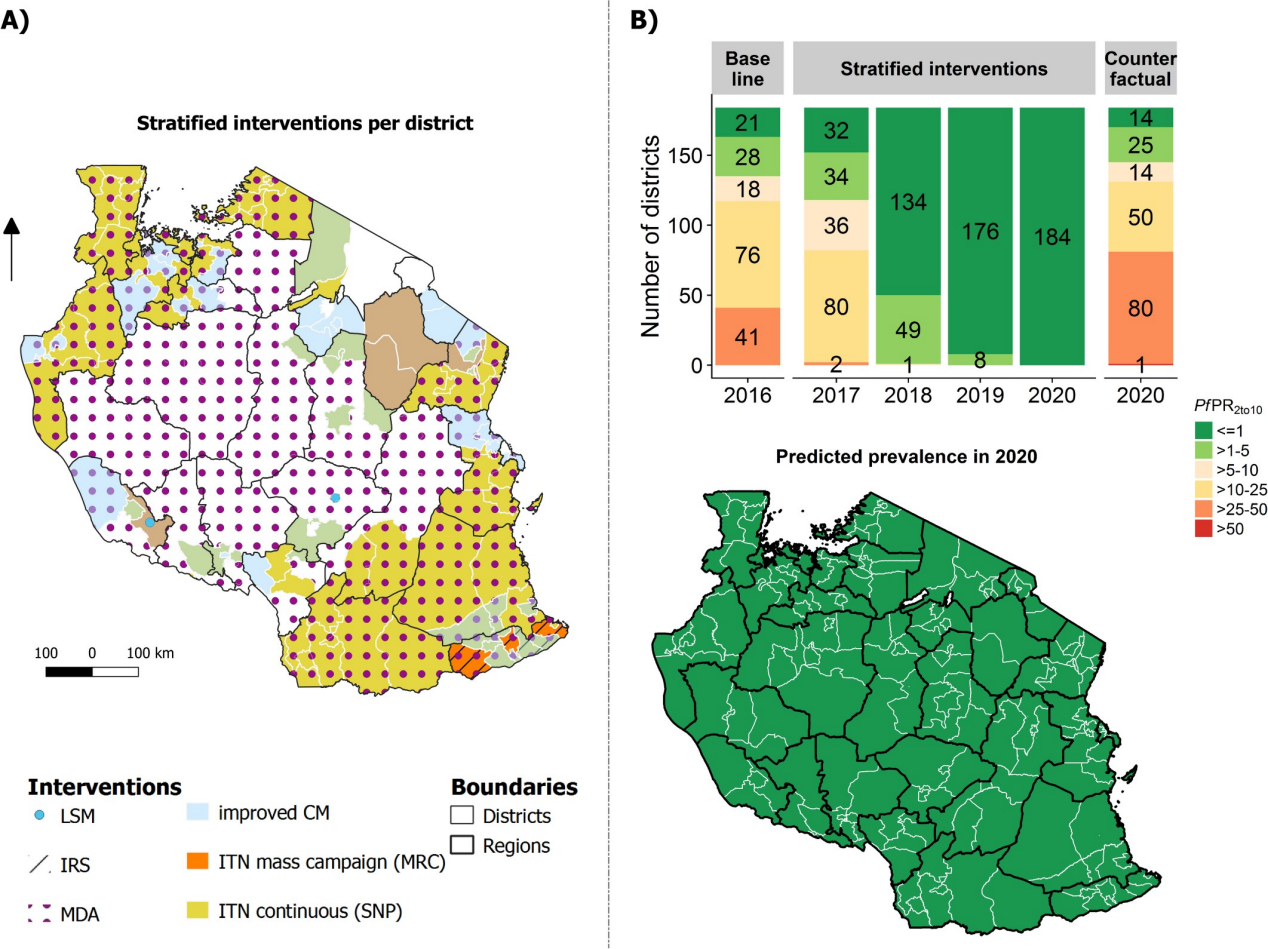
Strategy according to the selection of cost-minimised interventions that lead to the NSMP target (Strategy 3). (A) Allocation of interventions per council. (B) Predicted prevalence per council in 2020 shown in the map and number of councils per simulated endemic group for 2017–2020 shown in the bar chart.

The intervention stratification and intervention allocation that maximize impact on prevalence for a minimum cost are shown in Fig 7. The model predicted that the overall malaria prevalence in 2020 would be 0.43% (95%CI: 0.38% - 0.47%), corresponding to a prevalence reduction of 77.5% (95%CI: 70.5%-84.5%) between 2016 and 2020. This strategy was predicted to avert 102 million cases between 2017 and 2020 while saving around $153 million in treatment costs. The total costs for this strategy were estimated at around $891 million for 2017–2020 ($8.70 per case averted, $845 million excluding treatment costs).

### Comparison of intervention allocations per council

**Fig 8:**
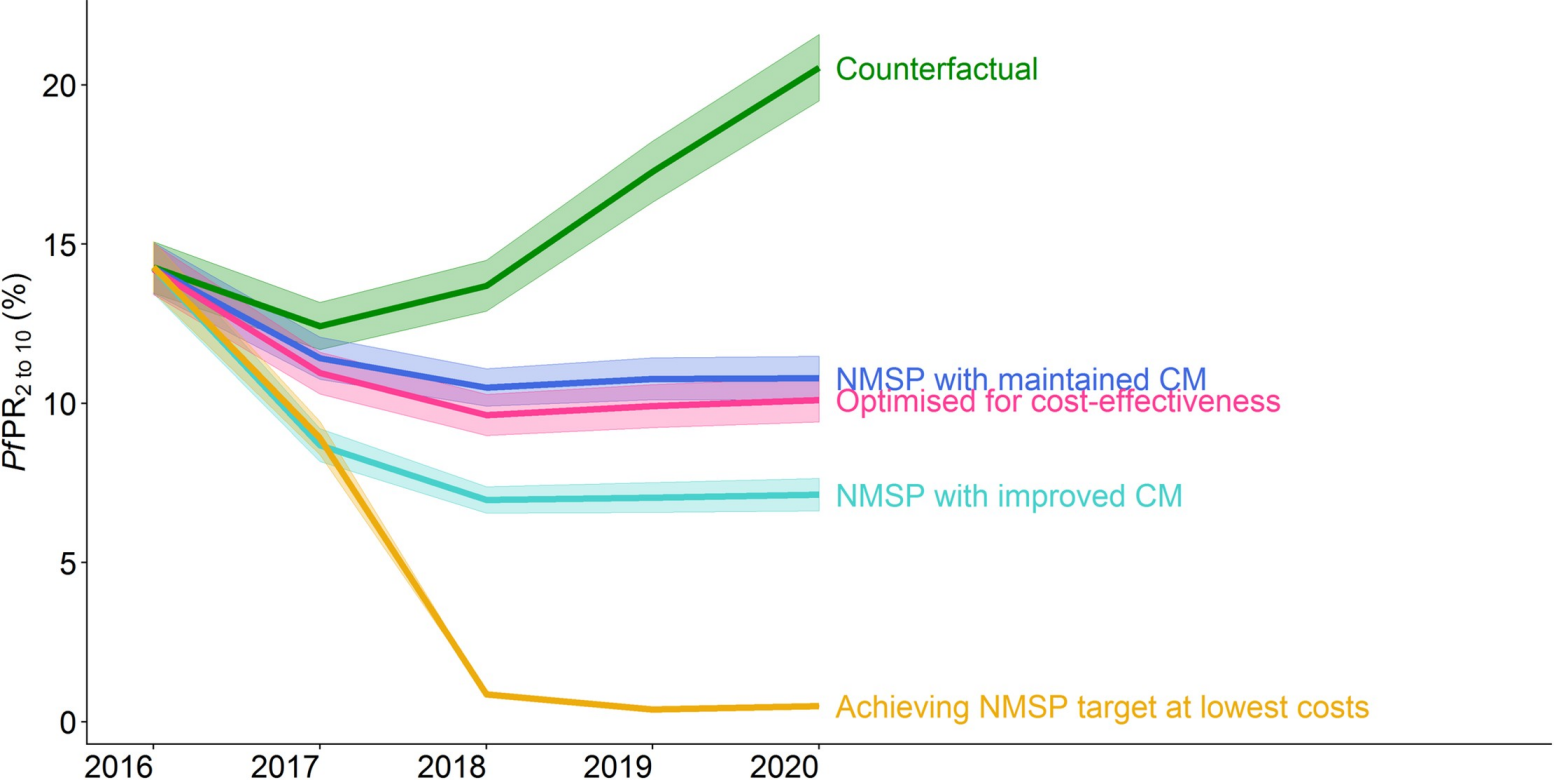
Predicted prevalence per strategy over time. The solid line shows the aggregated mean and the shaded area the 95% confidence interval based on heterogeneity among councils. The NMSP with maintained case management as well as the NMSP with improved case management refers to strategy 1, the most cost-effective intervention package to strategy 2, and the intervention package achieving the NMSP target to strategy 3. The counterfactual shows the simulated scenario with maintained case management levels and the discontinuation of vector control interventions.

At national level, the predicted impact over time was very similar between the simulated NMSP and the most cost-effective strategy, whereas the NMSP with an additional increase in case management led to a substantially lower prevalence–without reaching the national targeted prevalence of less than 1% (Fig 8), and timelines per region and impact cases are shown in S4 File. For 44 councils the interventions as allocated in the simulated NMSP were also the most cost-effective interventions and for one council the intervention achieving the national target at lowest costs. The intervention combinations achieving the national target at lowest costs were also the most cost-effective strategy for 22 councils.

## Discussion

Tanzania was formerly one of the world’s most highly endemic countries for *P*. *falciparum*. The NMCP has made a concerted effort to assemble all available data on malaria prevalance, vector compositions and resistance and intervention coverage at units of delivery deemed essential for a pragmatic, decentralised stratified future repsonse. Tanzania has demonstrated an impressive reduction in incidence and prevalence with scale-up of interventions over the last decade. Given the sub-national heterogenity of the malaria ecology in 2016, any future control demands a more nuanced selection of interventions at council levels. Exploring future projected impacts demands mathematical modelling to take into account all these factors, since the health impacts are not directly proportional to coverage and can take several years to emerge.

Simulation modelling was used to predict the impact of specific malaria interventions in 184 councils in mainland Tanzania. Council level transmission potential and the impact of past interventions were estimated by fitting the simulated prevalence to high-resolution prevalence estimates at council level. Core interventions included in the current NMSP supplemented or not by MDA were simulated when deployed alone or in combination. The impact of the intervention allocation according to the current NMSP was compared between vector control interventions alone or in combination with an increase of case management coverage. Three main strategies of interventions assigned at council level defined packages of interventions either chosen to minimise the incremental cost-effectiveness ratio or to meet the national target of reducing prevalence to less than 1% nationally by 2020 at minimal cost.

The requirements of the NMSP constrained the geographical units and timeframe for the analysis, the list of interventions, the target coverages (Table 2) and the measures that were optimised subject to these constraints. Administrative councils represent the operational units of the Tanzanian NMCP, and the geographical heterogeneities mean that the most appropriate intervention strategies are different by council, so a stratification and intervention allocation at this level is essential. In contrast, sub-council spatial heterogeneity was not considered, even though there is important smaller-area variation in intervention usage, components of vectorial capacity [82,83] and malaria endemicity [84,85]. While the EIR can be estimated based on only a few key parameters [86,87], the available mosquito bionomic data are inadequate for detailed parameterisations at council level or lower, and the workflow did not make use of the uncertainty or sub-council variation in the predictions from the geostatistical models.

The transmission potential and the starting point for the future interventions are important determinants of impact, and a very good fit to the prevalence for 2003 and 2016 was generally obtained by representing each council as a weighted average of settings varying in ITN historical coverage and pre-intervention EIR. Exceptions to the good fit to pre-intervention data were found in the Southern Zone, where the observed and geospatial model predicted prevalence sometimes exceeded the maximum in the simulated prevalence. The councils included in the school net program were estimated to have particularly high pre-intervention EIR, and it is unclear whether this is because they were indeed higher transmission areas, or if the differences arise because of over-estimation of the average impact of nets. This could have arisen because of reliance on the standard *OpenMalaria* parameterisations on data from experimental huts and field trials, where ITNs are unusually well implemented. *OpenMalaria* analyses of vaccine impacts suggest that the temporal trend in intermediate years is less important in determining future impacts [88], and the modelled time trends did not consider factors like housing, climate change and urbanisation [89,90] that are beyond the control of the NMCP. In general, the model fitted the time trends only moderately well and with considerable uncertainty, especially for 2012–2015 (which lacked both ITN coverage and prevalence data). This was especially true for councils with high ITN coverage, where estimates of prevalence and coverage were highly correlated. Moreover, the assumption of standard patterns of attrition and decay in effectiveness of ITNs imposed a maximum on the fitted rate of decline in prevalence, which in particular affected the estimates for councils without the school net program which made use of the ITN decay model.

The need for a limited number of intervention strata with standard fixed coverage targets meant that there was only one (high) target coverage for each intervention. This took into account technical, but not operational or financial feasibility [91], although variations in access, provider adherence, population compliance and other operational challenges [92–94] will generally lower effective coverage, especially for prompt and effective curative care [95]. An improvement in future stratification and intervention allocation exercises would be to define stratum-specific targets in terms of increments in coverage, rather than uniform target levels.

The methods used by the program to maintain coverage of the primary interventions (behaviour change communication campaigns to keep up use of ITNs, surveillance of clinical cases to keep up treatment rates) [22], were not analysed (thus avoiding double-counting of effects). Interventions not expected to affect prevalence in children and transmission in the community (*e*.*g*. intermittent preventive treatment for pregnant women) were also excluded, as were or others that the NMCP are not yet considering, such as vaccines [96], pyrethroid and piperonyl butoxide (PBO) treated nets [97,98], or other novel vector control tools [99]. Among interventions included, modelling larviciding is a particular challenge. Models of vector control, in general, are sensitive to local variations in mosquito ecology, but the feasibility and effectiveness and costs of larviciding are particularly challenging to predict because the achievable coverage depends critically on local larval ecology [100].

As a single intervention, ITN deployments were simulated to have the highest impact, irrespective of the distribution scheme. This is in agreement with other studies indicating that ITNs alone will only very gradually shift high prevalence areas (*Pf*PR >40%) to very low transmission and are unlikely to interrupt it [101–103]. In agreement with previous analyses using *OpenMalaria* the ITN effects synergised with increases in effective coverage of case management [104]. The simulations predicted high impact despite the assumed high resistance against pyrethroids, due to the physical barrier, while in practice this also depends on the vector population, biting behaviour and physical state of the net [105,106].

With no improvement in the effective coverage of case management, the simulated NMSP was predicted to only slightly reduce the baseline prevalence from 2016 until 2020, while an improvement in effective coverage could have a substantial effect. There may be some marginal benefits in optimising timing and effectiveness duration of larviciding, as there is limited evidence on large scale deployment of larviciding [107]. It could be that the reduction of emerging adult mosquitoes lasted not long enough to have a lasting impact on the coming transmission season [108]. Therefore the deployment of larviciding needs careful consideration, especially as larviciding is planned to be scaled up despite reported limitations in the impact depending on ingredient and conductor [109]. Overall, the predicted impact was very heterogeneous between councils with the impact depending on current and historical levels of transmission as extensively described previously [110–112]. This should encourage the use of council-level malaria control targets as in Ethiopia, where the transition of councils into the next lower prevalence endemicity class is one of the targets [113].

Only strategies including MDA in most councils appeared to be technically capable of achieving the original ambitious target of 1% national prevalence in 2020. Deployment of MDA at 80% coverage would be expected to bring the prevalence in most councils beneath this threshold (Fig 4). Though very high coverage of MDA has been achieved in Zanzibar [114], this would be extremely difficult to achieve and sustain across so many councils. Historically MDA programs have rarely achieved the coverage needed to meet such targets [92,115], and we concur with the WHO Global Malaria Program Global Technical Strategy [2] and other modelling studies [29] concluding that additional innovative tools are needed to achieve elimination. The strategy including MDA is clearly unaffordable with drug costs alone averaging $195 million per annum and the impact of such MDA would, in any case, be transient [28,33,116,117], especially as many areas in Tanzania still have high transmission potentially leading to high importation rates to areas with lower transmission and impeding sustainability of elimination efforts.

The intervention stratification that was most cost-effective conditional on these constraints comprised mostly ITNs, case management, with MDA in just a few councils. This is broadly in agreement with a previous intervention stratification proposed for Tanzania [29]. Previous modelling and cost-effectiveness studies found that IRS with CM were the most cost-effective combination if high coverage could be achieved and otherwise the combination of ITN and CM [40,45]. In our findings IRS was not included in any council, due to low impact and high costs. The costs of $30 per household sprayed correspond to the very high costs of targeted IRS, as shown in Tanzania for 2015 [118], and other distribution schemes may have lower costs and IRS might be cost-effective in specific areas [119,120]. In many low transmission settings, ITNs alone were the most cost-effective strategy, which agrees with Hansen *et al*. [121]; however, in practice, case-management might be prioritised over ITNs. The strategy optimised for cost-effectiveness suggested only an improvement in case management few very low transmission councils, whereas in practice discontinuation of vector control might not always be possible and certainly not recommendable without further evidence and in-depth analysis [112,122]. Moreover, resistance, although accounted for in this analysis, was not analysed in-depths and assumed to be homogeneous across the country with remained high effectiveness. In reality, the resistance varies per setting and species. To address high resistance, the WHO recommends deployment of PBO treated bed nets [123], which would influence the cost-effectiveness estimates of ITNs deployments.

The costing is from a provider perspective and is only indicative, including only consumable costs. It ignores salaries and infrastructure costs (much of which fall outside the NMCP budget), and economies of scale or scope (which would be particularly relevant for full costing of improved case management). The analysis does capture returns to scale [124], in terms of health impacts of varying coverage. The annual budget of the program was between $100 Mio and $150 Mio from 2010 until 2017 [125]. The most cost-effective simulated strategy was estimated to fit into this envelope, with simulated costs of $64 Mio per annum over four years, while the simulations of the current NMSP estimated its costs (including treatment costs) at $137 Mio. As the interactions between the modelling and NMCP evolve, it will become feasible to make more nuanced use of the data and to broaden the scope of the optimisation, while propagating uncertainties throughout the analysis. For instance, council level targets, varying target coverages [126,127] and sequential introduction of interventions [30] might be considered. The economic analysis could include additional costs, economies of scale and scope, optimisation within defined budgets, or extended time horizons with discounting.

## Conclusion

The developed modelling workflow generated information to understand the predicted impact of three main strategies, and allowed an *in silico* assessment of the targets included in the National Malaria Strategic Plan 2015–2020. This work has been used to suggest optimal stratification under the given operational and financial constraints. Results suggest that current interventions are not enough to reach the national aim of a prevalence lower than 1% by 2020 and that a revised strategic plan needs to consider additional interventions, especially in high transmission areas. The *OpenMalaria* model together with the calibration methodology developed here provides a helpful tool for assessing the feasibility of country specific targets and for determining alternative more impactful intervention combinations at sub-national level. Hence, the application of modelling can support strategic planning of malaria control, if based on realistic assumptions and done in close collaboration with the national malaria control programme.

## Supporting information

S1 File Developing a time-series risk map for Tanzania 1990–2017 based on empirical survey data. The file includes a comprehensive description of the geostatistical model used to derive annual prevalence estimates based on empirical survey data.
(DOCX)

S2 File Data sources, model parameterisation, calibration method, and future intervention deployment and effectiveness. (DOCX)

S3 File Cost calculations. (DOCX)

S4 File Additional results. The file includes a description of the estimated EIR in comparison to reported EIR obtained from literature, and figures of the sub-national fitting performance. It further includes the predicted intervention impact (prevalence and cases) per region and strategy, as well as population-weighted versus unweighted estimates.
(DOCX)
