# Supplementary material for: Simulating the council-specific impact of anti-malaria interventions: A tool to support malaria strategic planning in Tanzania
Source: PLoS One. 2020 Feb 19;15(2):e0228469. doi: 10.1371/journal.pone.0228469 (PMC7029840; doi:10.1371/journal.pone.0228469)
Supplement: S1 File — The file includes a comprehensive description of the geostatistical model used to derive annual prevalence estimates based on empirical survey data. (DOCX) [file pone.0228469.s001.docx]

**S1-file: Developing a time-series malaria risk map for Tanzania 1990-2017 based on empirical survey data**

**1. Assembly o*f Plasmodium* *falciparum* prevalence surveys**

Details of parasite prevalence survey data assembly, cleaning and geo-coding are provided elsewhere [1]. Important national household surveys of malaria infection prevalence included those undertaken in 2007/2008 [2], 198 clusters sampled as part of national scale-up of ITN programme in 2008 [NMCP, unpublished data], 2012 [3], 2015 [4] and 2017 [5]. In addition, national school-based surveys were undertaken in 2014-15 [6] and 2017 [NMCP, unpublished data].

5772 surveys undertaken at 4146 unique locations between August 1980 and May 2018 were included in the modelling of infection prevalence. The majority of the surveys were undertaken after 2010 (3464, 60%), Rapid Diagnostic Tests (RDTs) were used to detect malaria infection in 4040 (70%) surveys, microscopy in 1692 surveys (29%) and RDTs confirmed with microscopy in 40 (1%) of surveys. The spatial distribution of the data repository used in subsequent analysis is shown in Fig S1.1.


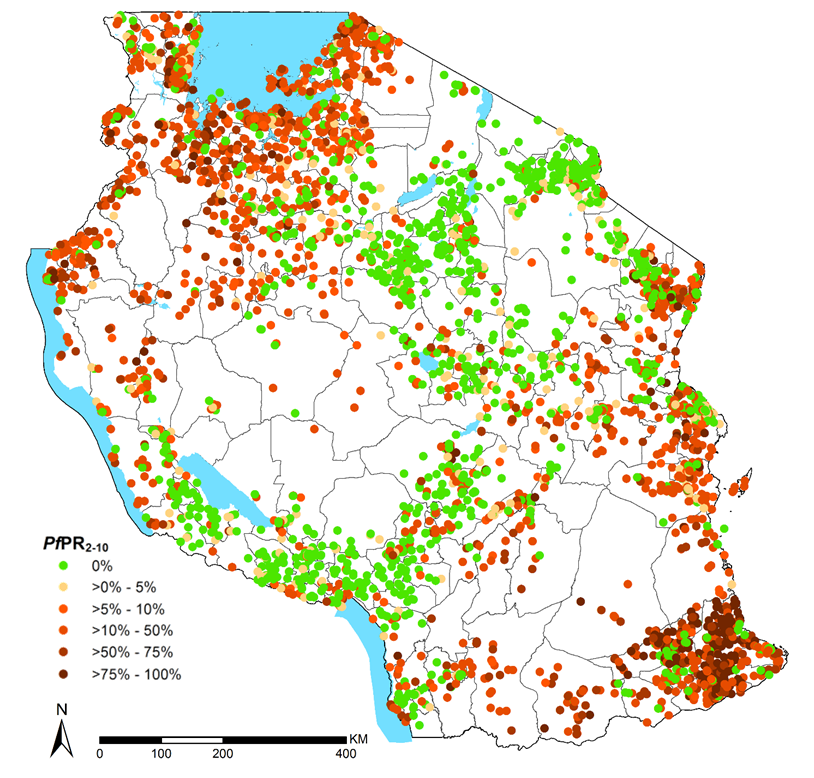


**Fig S1.1**: Distribution of 5772 malaria prevalence surveys undertaken at 4146 locations between August 1980 and May 2018. Surveys documented malaria prevalence across different age groups and were standardized to the 2 to 10 years age group (*Pf*PR_2-10_) [1,7].

**2. Geostatistical analysis**

Model Based Geostatistics (MBG) [8,9] is a likelihood-based approach that allows a prediction of a health outcome of interest using sparsely sampled data. This modelling framework has also been extended to interpolate both the spatial and temporal variation of disease prevalence through the analysis of repeated cross-sectional data [10]

To model changes in *Pf*PR_2_*_−_*_10_ by borrowing strength of information across time and space, an MBG model was used. In order to avoid estimates of prevalence resulting from misspecified regression relationships, a decision was made not to include covariates during the modelling exercise The inclusion of covariates in geostatistical models was examined by Weiss et al [11] and applied in Malawi and Tanzania [12,13] among others, whereas the specification of covariates was outside the scope of the presented work., following the approach of previous national malaria risk mapping efforts in Somalia [14], Kenya [15] and Malawi [16]. Let *x* be the location of a surveyed community in year *t*. Define a spatio-temporal Gaussian process, *S*(*x, t*), and unstructured random effects, *Z*(*x, t*), to account for the unexplained variation between and within communities, respectively. Conditionally on *S*(*x, t*) and *Z*(*x, t*), the counts of positive tests for *P. falciparum* were assumed to follow mutually independent binomial distributions with number of trials *N*, corresponding to number of sampled individuals, and probability of a positive outcome *p*(*x, t*) at location *x* (n=surveyed locations) and year *t* (1990 – 2017) given by

$log \left\{ \frac{p(x,t)}{1-p(x,t)} \right\} =\alpha+\beta mA+\gamma MA+S\left( x,t \right)+Z\left( x,t \right).$ (1)

where *mA* and *MA* are the minimum and maximum age among the sampled individuals at a location *x* and time *t*. In carrying the spatio-temporal predictions, *mA* and *MA* were set to 2 and 10 respectively to standardise to the age group 2-10 years. A stationary and isotropic Gaussian process for the spatio-temporal random effects is assumed *S*(*x, t*), with an exponential correlation function given as

$cor\left\{ S\left( x,t \right), S(x^{'},t^{'}) \right\}=e^{\left\{ -||u||/\varphi\right\}}e^{\left\{ -|v|/\psi\right\}}$ (2)

where *φ* and *ψ* are scale parameters which regulate the rate of decay of the spatial and temporal correlation for the increasing distance and time separation, respectively; *u* = ||*x* − $x^{'}$|| is the distance in space between the location of any two communities, one at *x* and the other at $x^{'}$; *ν* = |*t* − $t^{'}$| is the time separation in years between any two surveys.

The model parameters were estimated via Monte Carlo maximum likelihood in the R statistical software environment using the PrevMap package [17]. The targets for predictions were *Pf*PR_2−10_ over the 1×1 km regular grid covering the whole of mainland Tanzania. Maps of malaria risk were generated for every year from1990 to 2017 using ArcMap 10.5 (ESRI Inc., Redlands, CA, USA) (Figure SI 2).


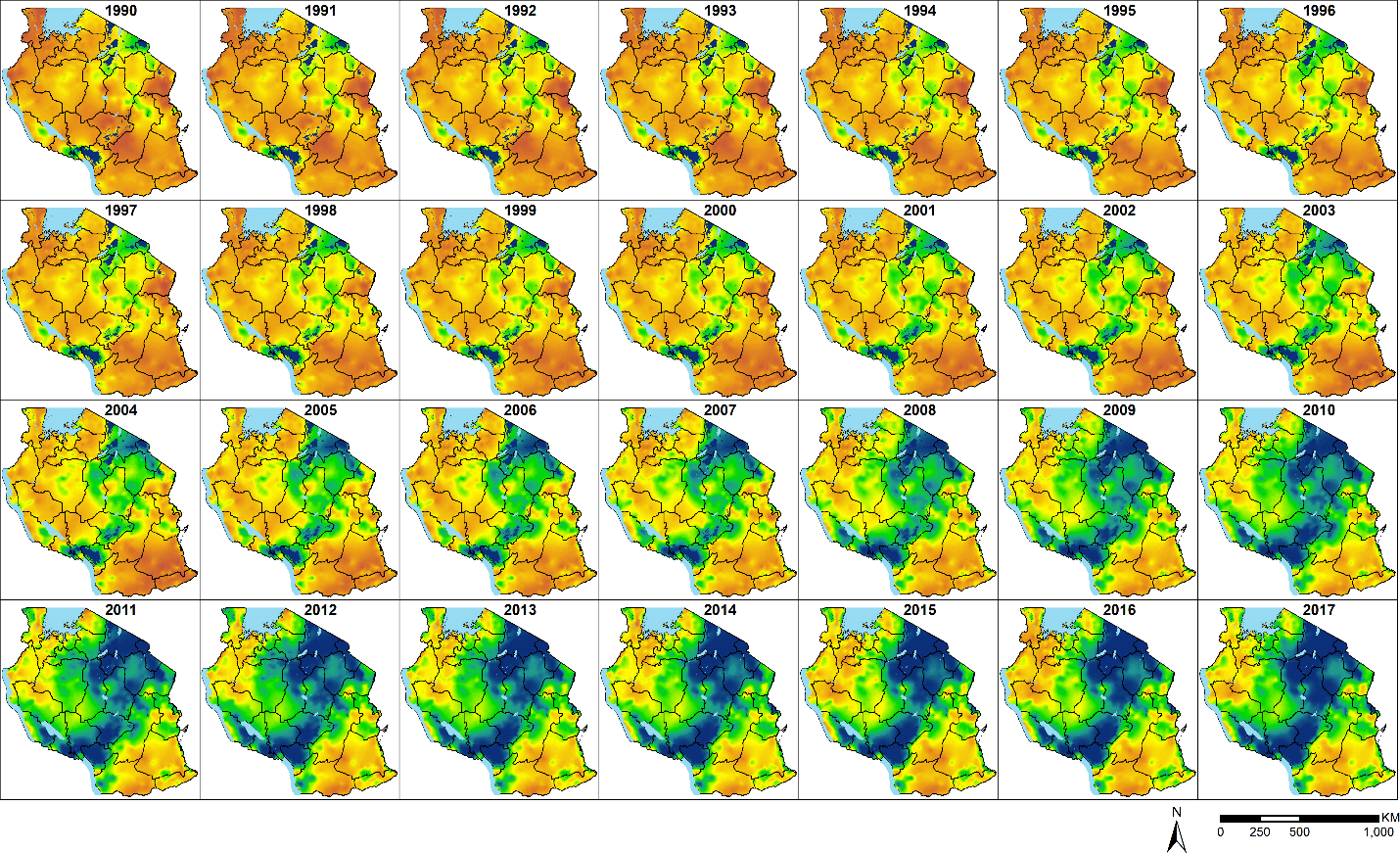


**Fig S1.2**: Annual predicted posterior mean community *Plasmodium falciparum* parasite rate standardized to the age group 2–10 years (*Pf*PR_2–10_) at 1 × 1 km spatial resolution from 1990 to 2017 ranging from zero (dark blue) to 94% (dark orange in Tanzania. Note all pixels are treated as zero *Pf*PR_2-10_ if they are represented by a temperature suitability index (TSI) of zero [18], these areas, located at high-altitude, have ambient temperatures that cannot support a period long enough for sporogony in the local dominant vectors and are therefore intrinsically climatically refractory to local malaria transmission.

**3. Model validation**

The model was validated using two methods: first by testing evidence against the residual spatio-temporal correlation in the data through the following variogram- based validation algorithm [10]: 1) Generate a point estimate $Z(x_{i}, t_{i})$ i.e. $\tilde{Z}(x_{i}, t_{i})$ from a non-spatio-temporal model, for each observed location $x_{i}$ and time $t_{i}$. ; 2) Permute the order of the data, including $\tilde{Z}(x_{i}, t_{i})$, while holding $(x_{i}, t_{i})$ fixed; 3) Compute the empirical semi-variogram for $\tilde{Z}(x_{i}, t_{i})$; 4) Repeat steps (1) and (2) a large number of times, say *B****;*** 4) Using the resulting *B* empirical variograms to generate 95% confidence intervals at each of the pre-defined distance bins. To conclude that there is no evidence against the adopted spatio-temporal model correlation the empirical semi-variogram from the original data must fall within the generated 95% confidence intervals (Fig S1.3). Secondly, validation statistics based on a 10% hold-out dataset (577 survey data points) for correlation against observed and predicted estimates of *Pf*PR_2-10_, bias and mean absolute error (Fig S1.4).


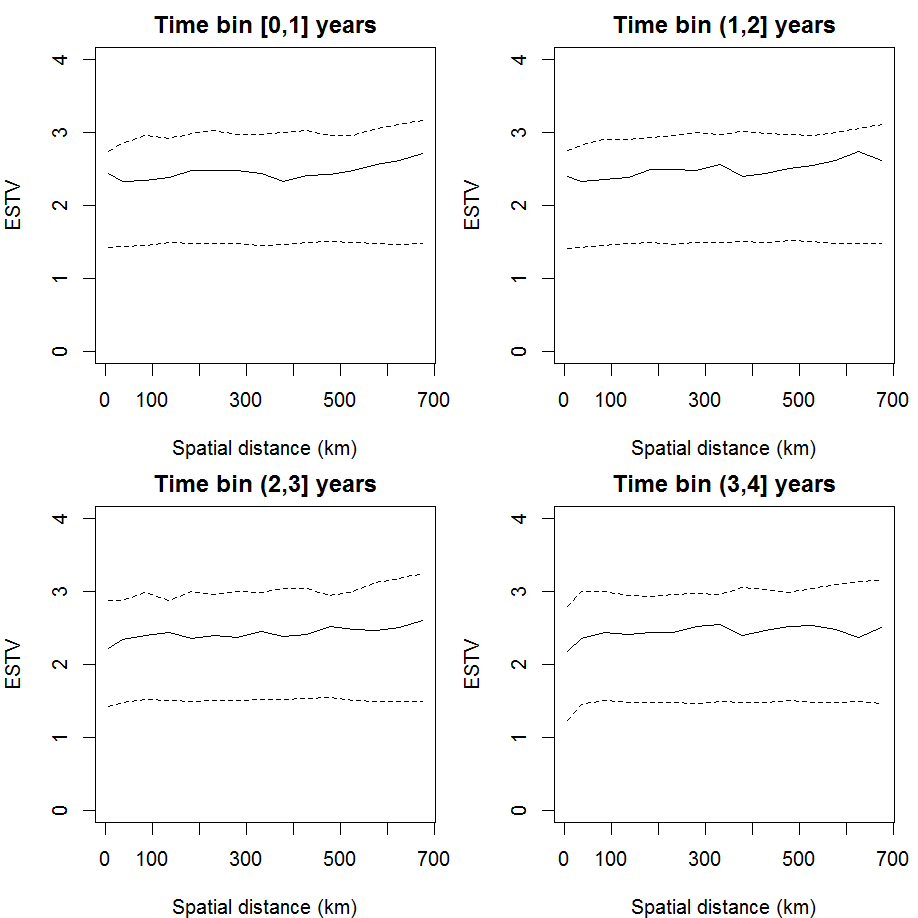


**Fig S1.3.** The solid line in each panel show the empirical spatial spatio-temporal variogram (ESTV), at four different time lags. The dashed lines represent the 95% confidence intervals generated under the hypothesis that the fitted spatio-temporal covariance function correspond to the true covariance function that generated the data. At any of the four time lags, the ESTV falls within the 95% bandwidth, which is evidence that the adopted covariance function is compatible with the data.


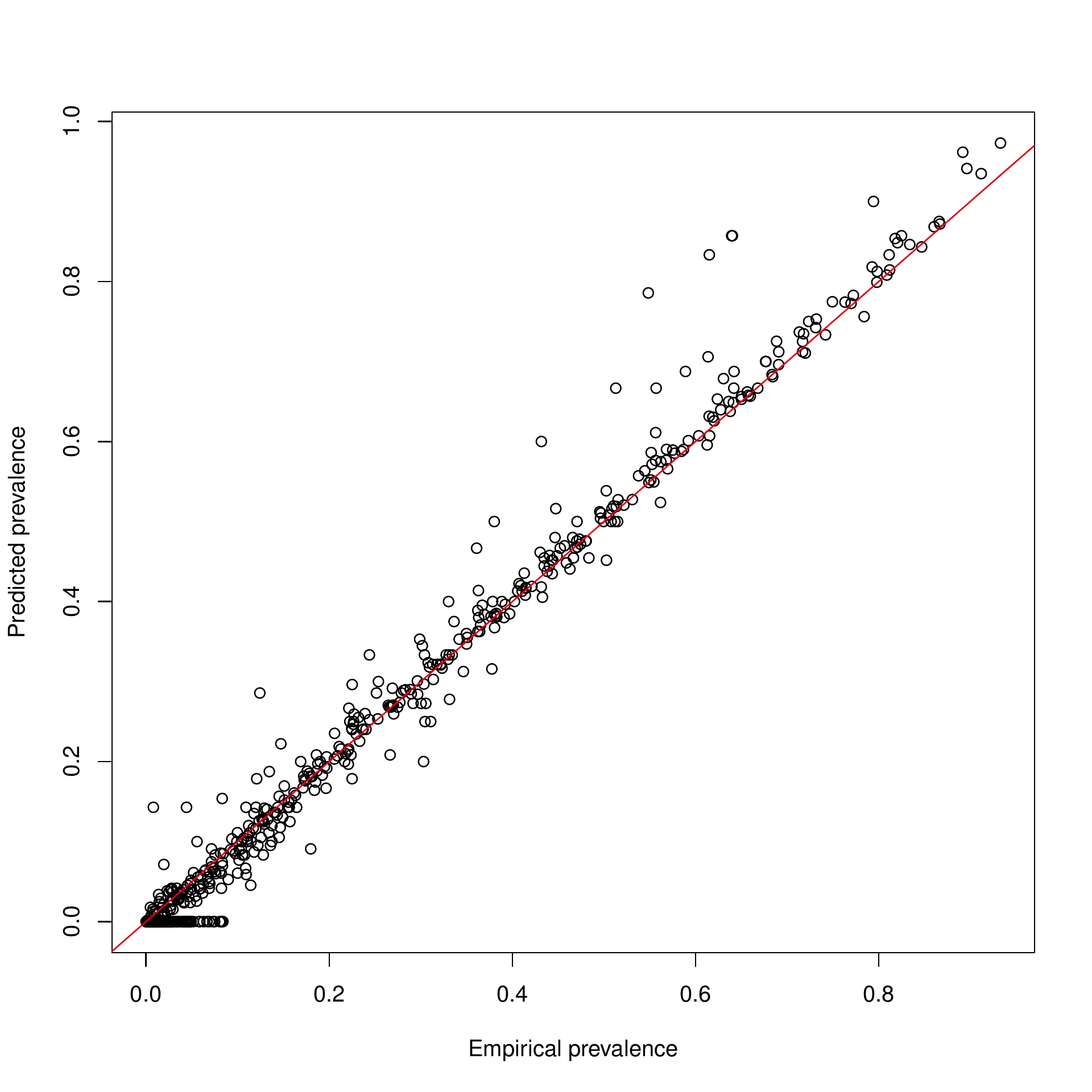


**Fig S1.4.** Scatter plot of the predicted prevalence from the geostatistical model (y-axis) against the empirical prevalence (x-axis). The predictive performance of the model was assessed on a test sample of 577 hold-out data points, resulting in a MAE of 0.7% and a bias of 0.4%.

**4. Acknowledgements**

The many scientists, archivists, institutions and national control programmes, who have helped assemble malaria data from across Tanzania over the last 22 years include the following:

Salim Abdulla, Mike Bangs, Jubilate Barnard, James Beard, Michael Beasley, Anders Bjorkman, Rene Bødker, Teun Bousema, Ilona Carneiro, Frank Chacky , Jong-Yil Chai, Prosper Chaki, Daniel Chandromohan, Mercy Chiduo, Jaffu Chilongola, Stephano Cosmas, Mark Divall, Stefan Dongus, Chris Drakeley, Mathew Dukes, Filbert Francis, Joanna Gallay**,** Yvonne Geissbühler, Blaise Genton, Nicodem J Govella, Francesco Grandesso, George Greer, Kara Hanson, Deus Ishengoma, Timona Jarha, Patrick Kachur, Rashid Khatib, Rose Kibe, Gerry Killeen, Safari Kinunghi, William Kisinza, Samson Kiware, Immo Kleinshmidt, Astrid Knoblauch, Karen Kramer, Paul Lango, Martha Lemnge, Christian Lengeler, Neil F Lobo, Zudson Lucas, Rose Lusinde, John Lusingu, Zawadi Mageni, Stephen Magesa, Julie Makani, Robert Malima, Alpha Malishee, Celine Mandara, Renata Mandike, Alpahaxar Manjurano, Tanya Marchant, Honoratia Masanja, Fabian Mashauri, Fabiano Massawe, Caroline Maxwell, Ben Mayala, Leonard Mboera, Peter McElroy, Clara Menendez, Sigsbert Mkude, Yeromin Mlacha, Bruno Mmbando, Ally Mohammed, Fabrizio Molteni, Dominic Mosha, Frank Mosha, Jackline Mosha, Daniel Msellemu, Hassan Mshinda, Frank Mtei, Zacharia J Mtema, Athuman Muhili, Theonest Mutabingwa, Victoria Mwakalinga, Dismas Mwalimu, Yusufu Mwita, Irene Mwoga, Isaack Namango, Kenneth Nchimbi, Rhita Njau, Baraka Nzobo, Fredros Okumo, John Owuor, Milka Owuor, Faith Patrick, Lynn Paxton, Emilie Pothin, Natasha Protopopoff, Hugh Reyburn, Mark Rowland, Tanya Russell, Sunil Sazawa, David Schellenberg, Self Shekalaghe, Clive Shiff, Method Segeja, Rosemary Silaa, Thomas Smith, Paul Smithson, Bob Snow, José Sousa-Figueiredo, Glades Stanley, Deborah Sumari, Thor Theander, Andrew Tomkins, Patrick Tungu, Jacobien Veenemans, Hans Verhoef, Ying Zhou

**References**

1. Snow RW, Sartorius B, Kyalo D, Maina J, Amratia P, Mundia CW, et al. The prevalence of Plasmodium falciparum in sub Saharan Africa since 1900. Nature. 2017;550:515–8.

2. TACAIDS, Zanzibar AIDS Commission (ZAC), National Bureau of Statistics (NBS) [Tanzania], MEASURE DHS, Macro International Inc. Tanzania HIV/AIDS and Malaria Indicator Survey 2007-08. Dar Es Salaam,Tanzania: Tanzania Commission for AIDS (TACAIDS); 2008.

3. TACAIDS, Zanzibar AIDS Commission (ZAC), National Bureau of Statistics (NBS) [Tanzania], Office of the Chief Government Statistician (OCGS), ICF International 2013. Tanzania HIV/AIDS and Malaria Indicator Survey 2011-12. Dar Es Salaam, Tanzania: TACAIDS, ZAC, NBS, OCGS, and ICF International; 2013.

4. MoHCDGEC, Ministry of Health (MoH) (Zanzibar), National Bureau of Statistics (NBS) [Tanzania], Office of Chief Government Statistician (OCGS), ICF International. Tanzania Demographic and Health Survey and Malaria Indicator Survey (TDHS-MIS) 2015-2016. Dar es Salaam, Tanzania and Rockville, Maryland, USA: MoHSW, MoH, NBS, OCGS, and ICF International; 2016.

5. Ministry of Health, Community Development, Gender, Elderly and Children (MoHCDGEC) [Tanzania Mainland], Ministry of Health (MoH) [Zanzibar], National Bureau of Statistics (NBS), Office of the Chief, Government Statistician (OCGS), ICF. Tanzania Malaria Indicator Survey 2017. Dar es Salaam, Tanzania, and Rockville, Maryland, USA: MoHCDGEC, MoH, NBS, OCGS, and ICF; 2017.

6. Chacky F, Runge M, Rumisha SF, Machafuko P, Chaki P, Massaga JJ, et al. Nationwide school malaria parasitaemia survey in public primary schools, the United Republic of Tanzania. Malar J. 2018;17:452.

7. Smith DL, Guerra CA, Snow RW, Hay SI. Standardizing estimates of the Plasmodium falciparum parasite rate. Malar J. 2007;6:131.

8. Diggle PJ, Tawn JA, Moyeed RA. Model-based geostatistics. J R Stat Soc Ser C Appl Stat. 1998;47:299–350.

9. Diggle PJ, Giorgi E. Model-based Geostatistics for Global Public Health: Methods and Applications. 1 edition. Boca Raton: Chapman and Hall/CRC; 2019.

10. Giorgi E, Osman AA, Hassan AH, Ali AA, Ibrahim F, Amran JGH, et al. Using non-exceedance probabilities of policy-relevant malaria prevalence thresholds to identify areas of low transmission in Somalia. Malar J. 2018;17:88.

11. Weiss DJ, Mappin B, Dalrymple U, Bhatt S, Cameron E, Hay SI, et al. Re-examining environmental correlates of Plasmodium falciparum malaria endemicity: a data-intensive variable selection approach. Malar J [Internet]. 2015 [cited 2016 Aug 30];14. Available from: http://www.ncbi.nlm.nih.gov/pmc/articles/PMC4333887/

12. Bennett A, Kazembe L, Mathanga DP, Kinyoki D, Ali D, Snow RW, et al. Mapping Malaria Transmission Intensity in Malawi, 2000–2010. Am J Trop Med Hyg. 2013;89:840–9.

13. Gosoniu L, Msengwa A, Lengeler C, Vounatsou P. Spatially Explicit Burden Estimates of Malaria in Tanzania: Bayesian Geostatistical Modeling of the Malaria Indicator Survey Data. PLoS ONE. 2012;7:e23966.

14. Giorgi E, Diggle PJ, Snow RW, Noor AM. Geostatistical methods for disease mapping and visualization using data from spatio-temporally referenced prevalence surveys. ArXiv180206359 Stat [Internet]. 2018 [cited 2018 Mar 23]; Available from: http://arxiv.org/abs/1802.06359

15. Macharia PM, Giorgi E, Noor AM, Waqo E, Kiptui R, Okiro EA, et al. Spatio-temporal analysis of Plasmodium falciparum prevalence to understand the past and chart the future of malaria control in Kenya. Malar J. 2018;17:340.

16. Chipeta MG, Giorgi E, Mategula D, Macharia PM, Ligomba C, Munyenyembe A, et al. Geostatistical analysis of Malawi’s changing malaria transmission from 2010 to 2017. Wellcome Open Res. 2019;4:57.

17. Giorgi E, Diggle PJ. PrevMap: An R Package for Prevalence Mapping. J Stat Softw. 2017;78:1–29.

18. Gething PW, Van Boeckel TP, Smith DL, Guerra CA, Patil AP, Snow RW, et al. Modelling the global constraints of temperature on transmission of Plasmodium falciparum and P. vivax. Parasit Vectors. 2011;4:92.
