# Supplementary material for: Simulating the council-specific impact of anti-malaria interventions: A tool to support malaria strategic planning in Tanzania
Source: PLoS One. 2020 Feb 19;15(2):e0228469. doi: 10.1371/journal.pone.0228469 (PMC7029840; doi:10.1371/journal.pone.0228469)
Supplement: S2 File — (DOCX) [file pone.0228469.s002.docx]

**S2-file: Data sources, model parameterisation, calibration method, and future intervention deployment and effectiveness**

**Content**

[1 Data and information sources 2](#_Toc22367551)

[2 Model parameterisation 4](#_Toc22367552)

[2.1 Vector bionomics 4](#_Toc22367553)

[2.2 Transmission intensity and seasonality 7](#_Toc22367554)

[2.3 Case management and control interventions 8](#_Toc22367555)

[3 Model calibration 15](#_Toc22367556)

[4 Simulated future scenarios 2017-2020 15](#_Toc22367557)

[References 16](#_Toc22367558)

# Data and information sources

**Table S2.1:** **Data and information sources used for parameterising *OpenMalaria* models and analysing outputs.**

| **Indicator** | **Definition** | **Use** | **Resolution** | **Age group** | **Years** | **Source** |
| --- | --- | --- | --- | --- | --- | --- |
| **Population and administrative units** | | | | | | |
| Administrative boundaries | Geographical areas that are defined by boundaries for administrative and operational purposes | To define the number of distinct models to run | NA | NA | 2018 | NMCP (unpublished data) |
| Population | Population estimates at high resolution | To extract population-weighted averages from high resolution ITN usage raster files | 5 × 5 km^2^ pixel raster | All | 2012 | WorldPop  <http://www.worldpop.org.uk/> |
|  | Total number of people per council | To calculate costs of interventions targeting a certain proportion of the population within councils | Council | All | 2012 | NBS [1]  <https://www.nbs.go.tz/> |
| Population per household | Number of people living in the same household | To determine the number of total household per council as denominator for IRS coverage calculation | Council | All | 2012 | NBS [1]  <https://www.nbs.go.tz/> |
| **Vector bionomics** | | | | | | |
| Vector occurrence and sporozoites rates | Counts of mosquito species per area and proportion of mosquitoes found with sporozoites per species | To determine the relative contribution per vector species to malaria transmission | Council | NA | 2016 | Entomological surveillance data  [NMCP, unpublished data] |
| Vector occurrence | Presence or absence of mosquito species at different locations and times | To determine the relative contribution per vector species to malaria transmission | Study sites | NA | NA | Personal communication, assembled literature database from the Ifakara Health Institute |
| Indoor/ outdoor biting | % of vectors affected by indoor interventions | To inform vector bionomics parameter | Study site | NA | 2016 | [3] |
| Resistance | Assumed resistance against pyrethroids (% mortality) | To inform assumptions about insecticide resistance | Council | NA | 2014, 2015 | NIMR, resistance reports  [4,5] |
| **Transmission and seasonality** | | | | | | |
| Pre-intervention Transmission intensity | Infectious bites per person per year before large scale up of control interventions | No data available for all councils and parameter was estimated | | | | |
| *Pf*PR | % of children who were tested positive using an mRDT, out of all children tested with mRDT | To compare with other prevalence sources and the simulated prevalence | Region | 0-5 years | 2008, 2012, 2016 | MIS [9–12]  <https://www.statcompiler.com/> |
|  | Model predicted prevalence (*Pf*PR_2to10_) | To fit to simulated prevalence to obtain weighted predictions per councils | 5 × 5 sq km pixel raster | 2-10 years | 1990 - 2017 | Described in S1 file |
| Seasonality | Monthly climate suitability for malaria transmission | To define the seasonal trend of transmission | Council | All | 1983-2010 | TMA [2]  [http://maproom.meteo.go.tz/ maproom/index.html](http://maproom.meteo.go.tz/%20maproom/index.html) |
| **Case management and control interventions** | | | | | | |
| CM | % of febrile children who were taken to any health facility at any time during the last two weeks out of all febrile children | To approximate the effective treatment coverage between 2003 and 2016 | Region | 0-5 years | 2008, 2012, 2016 | MIS [9–12]  <https://www.statcompiler.com/> |
| ITNs distributed | 1) total number of nets distributed  2) number of nets distributed per population | To inform the timing of ITN deployment  To distinguish between mass campaign and cumulative distribution | Council | Mixed | 2004-2016 | Commodity data  [NMCP, unpublished data] |
| ITN decay | % of nets remaining over time | To determine functions for attrition of nets 1) for calculating the ITN usage at the time of the last campaign, and 2) for use as fitting parameter | *) |  |  | VectorWorks (NetCALC)  <https://www.vector-works.org/resources/netcalc-planning-tool/>  [7]; [8] |
| ITN coverage  (effective usage) | % of the total population who slept under an ITN the night previous to survey | To calculate the ITN usage at the time of the last campaign in 2012 and 2016 | Region | 0-5 years | 2008, 2010, 2012, 2016 | MIS, DHS [9–12]  <https://www.statcompiler.com/> |
|  |  | To inform annual ITN coverage between 2003 and 2011 | 5 × 5 sq km pixel raster | All | 2003 - 2010 | MAP [13]  <https://map.ox.ac.uk> |
| IRS coverage | % of population protected by IRS | To calculate historical IRS coverage | Council | All | 2007-2016 | Commodity data [NMCP, unpublished data],  IRS spraying reports [6] |

NA = Not applicable

*) Decay functions have been modelled based on multiple field observations as described in the references.

# Model parameterisation

The setting-specific model parameterisation is separated into four sections 1) vector bionomics, 2) transmission intensity and seasonality, and 3) historical interventions. Each section describes the processes applied to derive model parameters from the available data and information, or the assumptions made in case of insufficient data.

## Vector bionomics

The vector bionomics refers to multiple characteristics attributable to the malaria vector, including the species, the human blood index, gonotrophic cycle, susceptibility to indoor interventions, resistance against insecticides, and contribution to transmission. Below it is briefly summarised for each of the attributes how the parameters were derived and the actual parameters are shown in Table S2.2.

### Mosquito species

The main malaria vectors in Tanzania are *An. funestus*, *An. gambiae s.s* and *An. arabiensis*. The parameterisations for the different mosquito species were already realised elsewhere [14,15], and only their contribution to transmission and proportion being affected by indoor interventions were adjusted for the setting specific models. In regions with no information about the vector populations and infectiousness fixed relative contributions were assumed with 80% *An. funestus*, 10% *An. gambiae s.s* and 10% *An. arabiensis*.

### Human blood index

The tendency of mosquitoes to bite on humans vs animals also influences the malaria transmission as well as the impact of interventions. The parameterisation of the human blood index for each species was extracted from the literature [14,15], assuming *An. arabiensis* to be more zoophilic than *An. funestus* and *An. gambiae s.s.*

### Gonotrophic cycle

The proportion of mosquitoes host seeking on the same day as ovipositing is also species specific and will influence the timing of the transmission cycle and therefore the impact of the interventions. The values for its parameterisation were selected according to an available parameterisation by Chitnis et al., based on literature [14,15].

### Susceptibility to indoor interventions

The susceptibility of a mosquito to indoor interventions depends on its biting pattern and level of endophily. The assumptions made in the model were based on knowledge from local experts and published literature [16,17]. *An. funestus* and *An. gambiae s.s* are mostly indoor biting while *An. arabiensis* mostly feeds outdoors [16,17]. Hence, it was assumed that indoor interventions would affect 90% of *An. funestus,* 80%, of *An. gambiae s.s,* and 30% of *An. arabiensis* mosquitoes.

### Insecticide resistance

Until 2010 all vectors were assumed to be susceptible against pyrethroids, after that the assumed pyrethroid resistance was 80%. The resistance level was assumed to be the same in all regions and for all vectors [4,18]. All species were assumed to be susceptible for insecticides used for IRS.

### Contribution of vectors to malaria transmission

In Tanzania, entomological surveys were conducted in selected sentinel sites since mainly by the National Institute for Medical Research (NIMR), for insecticide resistance monitoring. In August 2016, the NMCP started entomological surveillance throughout the country [NMCP, personal communication]. The mosquitoes were caught with light traps. At the time of the analysis, in November 2016, 874 mosquitoes were processed from thirteen councils, and the number of mosquitoes with infectious sporozoites was reported per council, but no information was available on the total number of mosquitoes caught (NMCP, unpublished data). For those, four mosquito species were listed, namely *An. gambiae s.s.,* *An. arabiensis*, *An. funestus s.s*., and *An. leesoni*. The latter two were combined into one category ‘*An. funestus’*, due to low numbers for *An. leesoni*. Reports on insecticide resistance were accessed from NIMR [4] to complete the collation of information on occurrence and vector composition for each council in Tanzania, which also included a comprehensive extraction of entomological data from the literature (Dr Fredros Okumu, Ifakara health Institute, personal communication) [3,4,16,18–27].

When available, the entomological surveillance data were used to estimate the contribution to transmission per vector species. The relative contribution of a vector species to the annual EIR was defined as the product of the relative infectiousness and occurrence of a mosquito species. For that, the relative occurrence of each vector species was assumed to be reflected by the relative frequency of the analysed mosquitoes included in the preliminary results report. Moreover, the same mosquito-specific infectiousness was assumed across all councils and calculated as the proportion of infectious mosquitoes per species (Table S2.2). In the absence of available entomological surveillance data, the collated database was screened for the occurrence of vector species*.* If all three vectors were known to be present, their contribution to EIR was distributed as 45% for *An. funestus,* 45% for *An. gambiae s.s*. and 10% for *An. arabiensis,* otherwise 80% for *An. funestus*, 10% for *An. gambiae s.s.* and 10% for *An. arabiensis* (Fig S2.1).

**Table S2.2: Malaria vector characteristics.**

| **Species specific parameter** | ***An. funestus s.l*** | ***An. gambiae s.s.*** | ***An. arabiensis*** |
| --- | --- | --- | --- |
| **Seasonality** | Same for all species following council specific climate suitability trends | | |
| **Pyrethroid resistance** | before 2011: 0%  after 2011: 80% | | |
| **Affected by indoor intervention** | 90% | 80% | 30% |
| **Human blood index** | 0.980 | 0.939 | 0.871 |
| **Proportion of mosquitoes host seeking on same day as ovipositing** | 0.616 | 0.313 | 0.313 |
| **Sporozoites rate** | 0.694 | 0.264 | 0.042 |
| **Contribution to EIR (assumed in the absence of data)**  If no information on vector occurence  If presence of all three vectors | 80%  45% | 10%  45% | 10%  10% |
| **Previously determined base parameters as described by Chitnis et al. [28] with values, accessible on the OpenMalaria wiki [14].** | | | |
| **Duration of resting stage**  “Time required for a mosquito that has encountered a host to return to host-  seeking” | 3 | 3 | 3 |
| **Extrinsic incubation period**  “The time required for sporozoites to develop in the mosquito” | 11 | 11 | 11 |
| **Proportion of eggs laid same day**  “Proportion of host-seeking parous mosquitoes that have laid eggs that day.” | 0.616 | 0.313 | 0.313 |
| **Duration of host seeking**  “Maximum length of time that a mosquito searches for a host in one day if it is unsuccessful.” | 0.33 | 0.33 | 0.33 |
| **Parous rate**  “Proportion of host-seeking mosquitoes that have laid eggs at least once.” | 0.611 | 0.623 | 0.623 |
| **Availability variance**  “Total availability rate of all nonhuman hosts” | 0 | 0 | 0 |
| **Biting probability**  “Probability that a mosquito bites a human after encountering one.” | 0.95 | 0.95 | 0.95 |
| **Probability finding resting site**  “Probability that a mosquito finds a resting place after biting a human.” | 0.95 | 0.95 | 0.95 |
| **Probability of surviving resting stage**  “Probability that a mosquito survives the resting phase after biting a human.” | 0.99 | 0.99 | 0.99 |
| **Ovipositing probability**  “Probability that a mosquito lays eggs and returns to host-seeking after biting a human” | 0.88 | 0.88 | 0.88 |


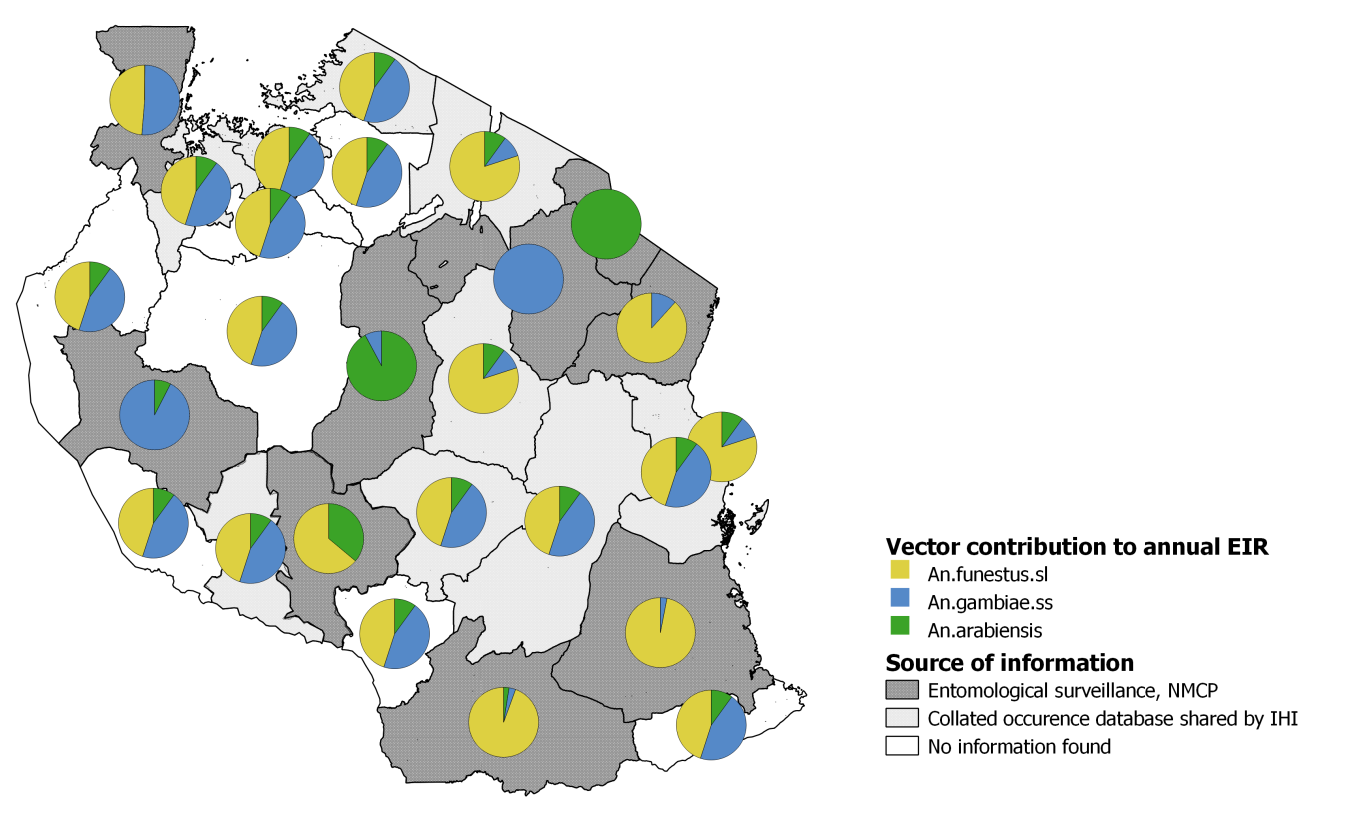


**Fig S2.1: Assumed geographic distribution of the contribution to malaria transmission per vector species.**

Dark grey areas show estimated contribution of different vectors to EIR, based on preliminary entomological data send by NMCP (NMCP unpublished data). Light grey areas show estimates derived based on a collated occurrence database. White areas show assumed vector contributions to EIR, based on a defined default distribution after discussions with experts, for those regions with missing data.

## Transmission intensity and seasonality

### Pre-intervention transmission intensity

The entomological inoculation rate (EIR) determines the prevalence and intervention impact and resurgence, and historically EIRs from less than zero up to 500 [29] or 1000 [30] were reported. The relationship between prevalence and EIR is log-linear, starting to saturate at an EIR around 100 and is mediated depending on case management, seasonality and other factors [30–32]. Therefore, six EIR levels were selected, ranging from 0 to 550 (0, 4, 16, 54, 120, 550).

### Seasonality

The vector abundance was assumed to follow the same trend as the monthly climate suitability for malaria transmission [2,33] and to be the same for all mosquito species included in the model. The climate suitability indicator combines temperature (mean temperature between 18°C and 32°C), rainfall (“precipitation accumulation > 80 mm”) and humidity (> 60%) per month [2,33](Fig S2.2).


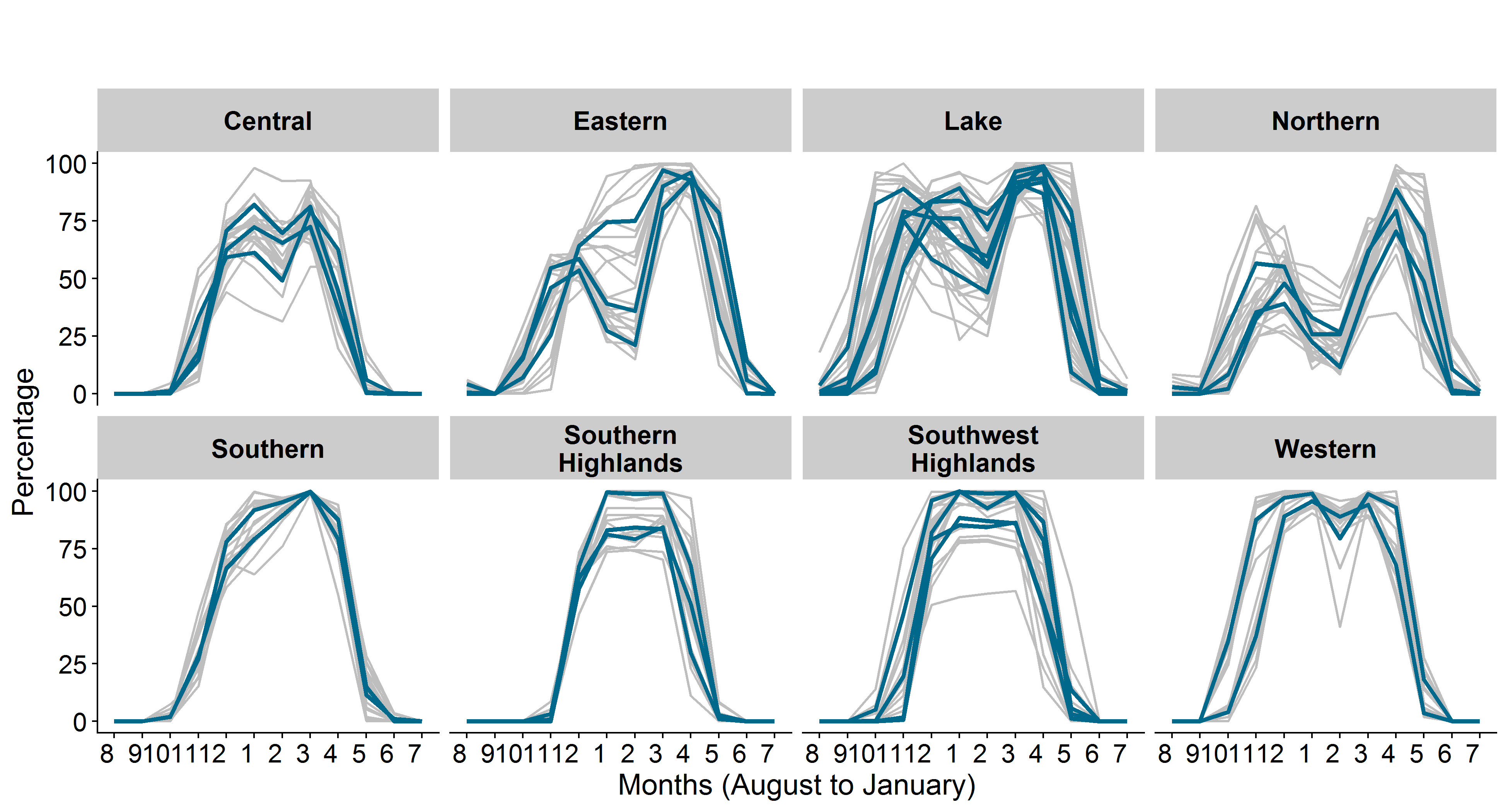


**Fig S2.2: Monthly climate suitability for malaria transmission grouped by geographical zones.**

The grey lines show the councils and the blue line aggregated mean per region. Data source: Tanzania Meteorological Agency [2,33].

## Case management and control interventions

### Case management

According to the national MIS survey results from 2016, around 66% of febrile children under the age of five years sought treatment (Fig S2.3), 36% were tested for malaria, and 43.4% received any antimalarial, of which 85% were ACTs [34]. For simplicity only the care-seeking estimates was used, assuming that out of all febrile children accessing care 59.7% are effectively treated, based on a previous study assessing effective treatment in Sub Saharan Africa [35]. Hence, the effective treatment coverage was calculated by multiplying the proportion of children accessing care per region with the scaling factor of 0.6, estimated at national level for Tanzania [35].


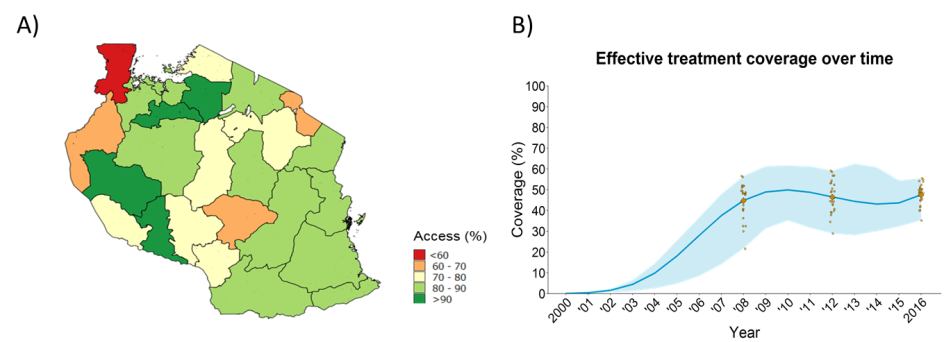


**Fig S2.3: Historical case management.**

A) Geographic distribution of the proportion of children accessing any health facility within two weeks prior survey as estimated in the TDHS-MIS 2015/16. B) National temporal trend of the estimated proportion of individuals effectively being cured when they have a fever (effective treatment coverage). The orange dots correspond to regional estimates for care seeking behaviour from malaria indicator surveys [9,11,12], scaled with the estimate for effective treatment [35]. The solid blue line corresponds to a fitted line for effective treatment based on the survey data with the assumption of no effective treatment before 2003. The light blue area corresponds to the minimum and maximum of the fitted lines.

### Insecticide-treated bed nets

In Tanzania, three large ITN mass campaigns were conducted: the universal coverage campaign (UCC) from 2009 to 2010, the keep up campaign from 2010 to 2011, and the mass replacement campaign from 2016 to 2017. In addition, continuous deployments mechanisms exist and the most relevant one at the community level is the school net program implemented since 2013 in three regions. The reported ITN usage from MIS surveys roughly as well as the estimated ITN usage from MAP roughly follow the trend in number of nets distributed, but did not capture the differences between the deployment schemes since 2013 (Fig S2.4).


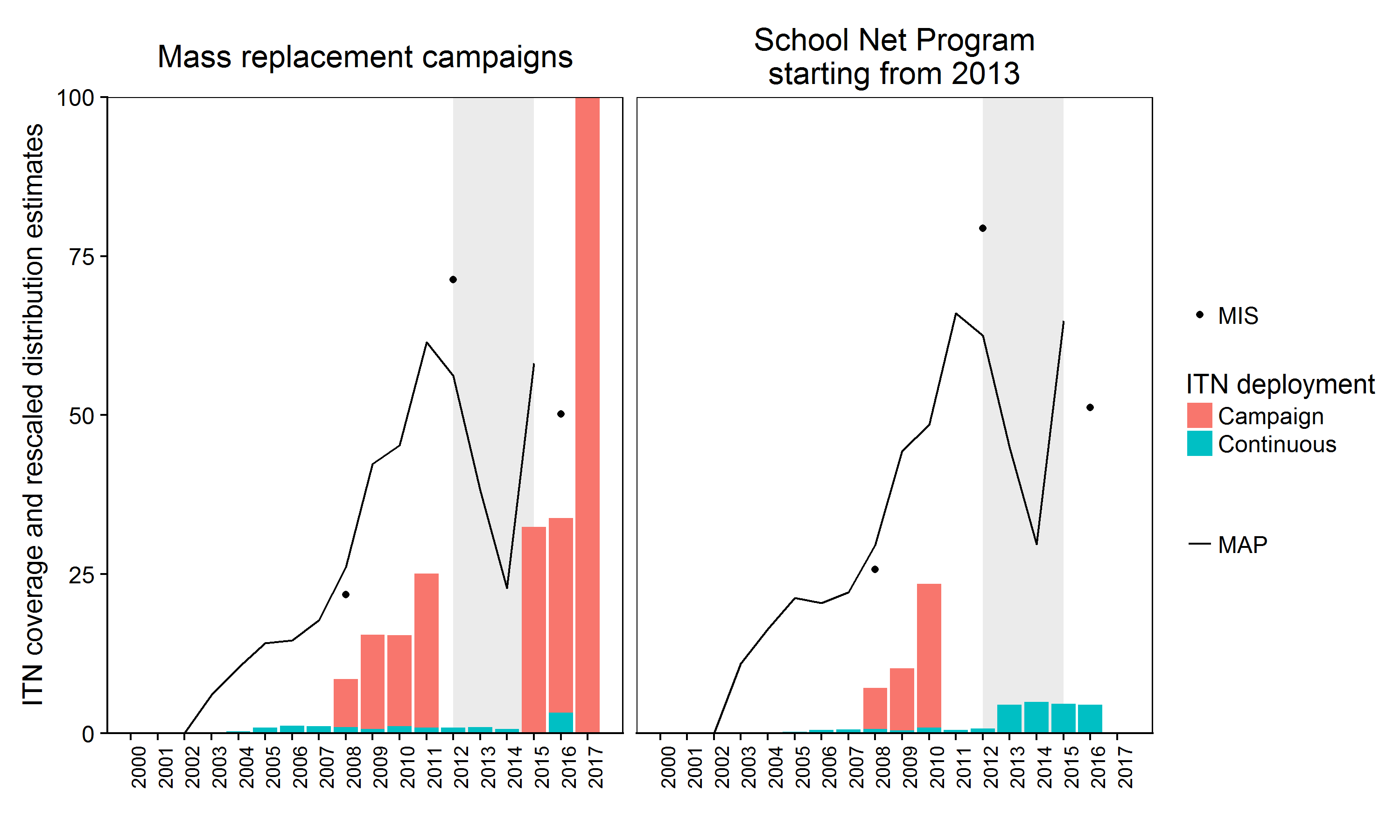


**Fig S2.4: Historical ITN coverage and distribution 2000-2016/17.**

Average estimates of ITN usage from household surveys (MIS) and the Malaria Atlas Project (MAP) between regions receiving ITNs mostly through mass campaigns after 2012 (n=23) and between regions receiving ITNs mostly through school net distributions after 2012 (n=3). The grey area shows the time between 2012 and 2015 with no available data on ITN usage from household surveys and no mass deployment campaigns of ITNs.

The estimated ITN coverage trends from MAP were similar among councils within regions (Fig S2.5).


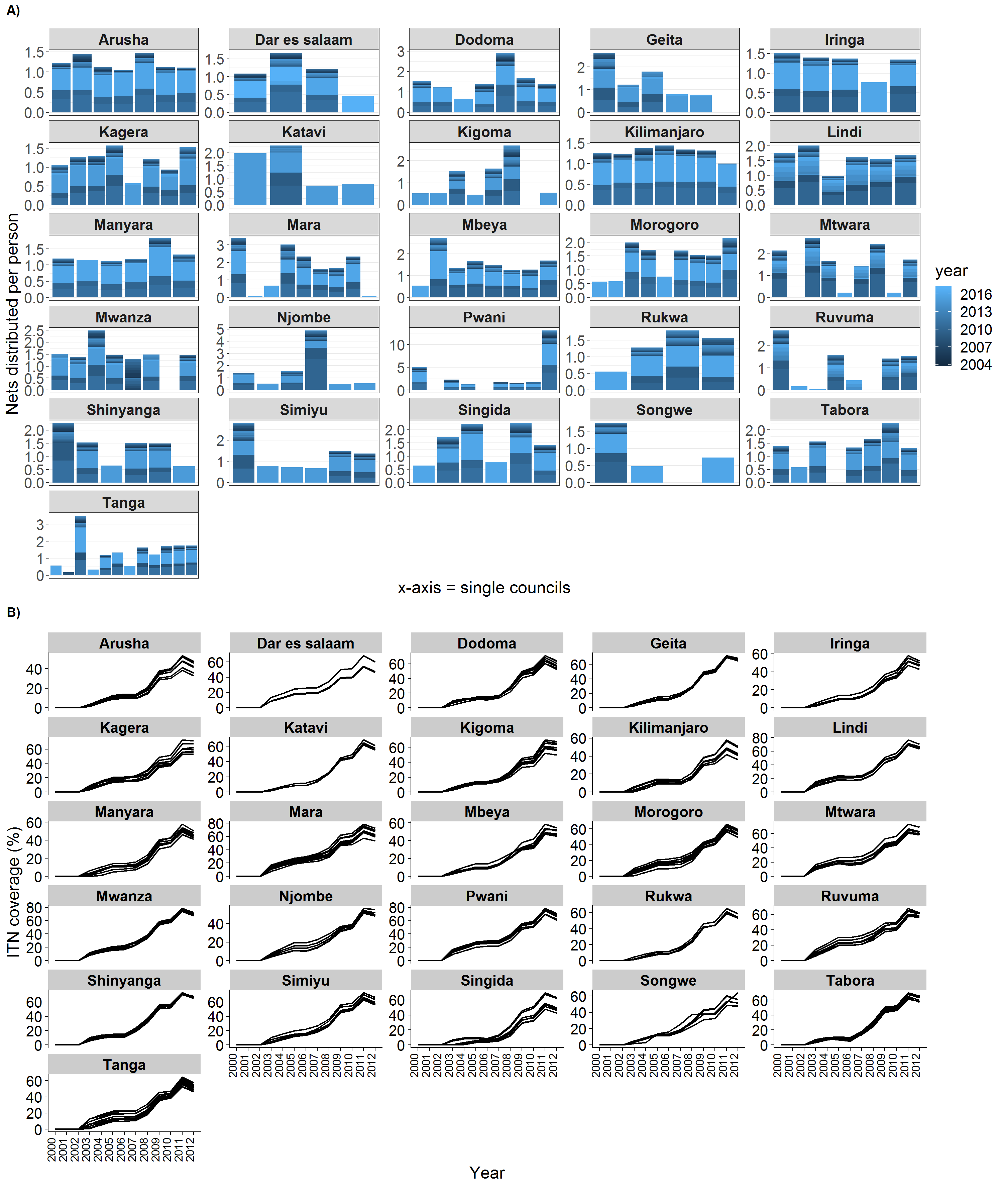


**Fig S2.5: Historical ITN usage per council and region between 2000 and 2012.**

Estimates obtained from MAP mean raster files.

**Simulated ITN deployments and coverage**

The model, does not distinguish between ITN coverage and usage, using the terms interchangeably, referring to the proportion of the people protected by a net from all indoor bites. The figure below (Fig S2.6) shows the assumed ITN use in 2016 per region and the national aggregated trend over time with range per regions used in the simulations (for each region separate trends).

**
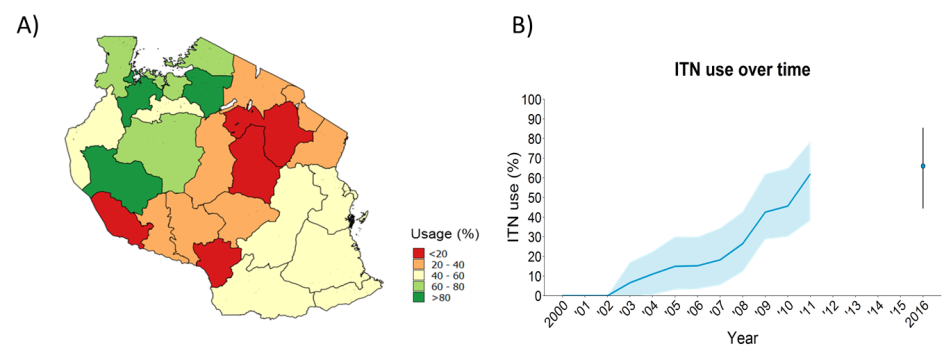
**

**Fig S2.6: Historical ITN use.**

**A)** Geographic distribution of the proportion of the population who slept under any ITN the night prior the survey in 2016; B) National temporal trend of the estimated proportion of the population who use their bed between 2000 and 2011. The map shows reported ITN usage obtained from the THDS-MIS 2016 [12]. The solid blue line corresponds to population weighted median ITN usage extracted from ITN usage raster files downloaded from the MAP website [38] and population estimates from WorldPop [39]. The light blue area corresponds to minimum and maximum estimates among regions. The blue dot and error bar in 2016 correspond to the estimates of the proportion of the population who slept under any ITN the night before the survey (mean and range among regions) [12].

Between the years 2003 and 2011, ITN distribution data (nets delivered per person), per region, showed a very similar trend as the trend of the ITN usage estimated by the Malaria Atlas Project (MAP) [38]. Hence, the estimates of the population-weighted mean ITN usage were extracted for each region and used to define the historical ITN coverage. A step function of one year effectiveness was assumed to exactly match the estimated annual estimates.

Between 2008 and 2011, the “catch up” (2008-2010) and “keep up” (2010-2011) campaigns were considered as one with universal deployment in January 2011. The regional estimates of the proportion of the population using their nets the night before the survey was used as a proxy for bed net usage among all age groups and was extracted from the MIS 2012 survey data. The presumed effective use of nets during the 2011 campaign was back-calculated using the date of the distribution and the net use from 2012, assuming exponential attrition of nets with a half-life of three years [10,11].

Between 2012 and 2016, nets were distributed to school children annually in three regions, namely Lindi, Mtwara, and Ruvuma [40], while no large scale net distribution took place in other regions. For councils without ITN deployments between those years, the decay of nets distributed in 2011 was estimated during the model calibration, whereas, for councils included in the school net program, the annual ITN coverage between 2013 and 2016 was estimated. This allowed to reproduce the reported increase in prevalence between the MIS surveys in 2012 and 2016 [11,12] in some councils. ITN decay curves were estimated from the data and compared with the assumed ITN decay used in NetCALC [7] (NetCalc 3.2 background document available from <https://www.vector-works.org/resources/netcalc-planning-tool/>).

In 2016, a mass distribution of ITNs was conducted at the same time than the TDHS-MIS 2015-16 surveys. The coverage of ITN distribution was therefore adjusted to account for the difference of timing between campaign and survey. For the adjustment, the regions were divided into three groups: 1) regions in which the net distribution took place before the survey, 2) regions in which the net distribution took place after the survey 3) regions in which there are no mass campaigns but only continuous (annual) school net distribution. For the regions, surveyed before the mass campaign in 2016, a scaling factor was derived from those regions surveyed before and after the mass campaign in 2016, using ITN usage estimates from the MIS surveys in 2010, 2012 and 2016. The scaling factor was derived by estimating the relative increase in coverage after mass campaign depending on the coverage before the mass campaign (Fig S2.7). The ITN groups and ITN usage estimates per region are shown in Table S2.3. The adjusted ITN use among the whole population in 2016 was estimated to be on average 66% ranging from 44.4% to 85% across the regions (Fig S2.7). The parameterisation of ITN efficacy is described elsewhere [8].


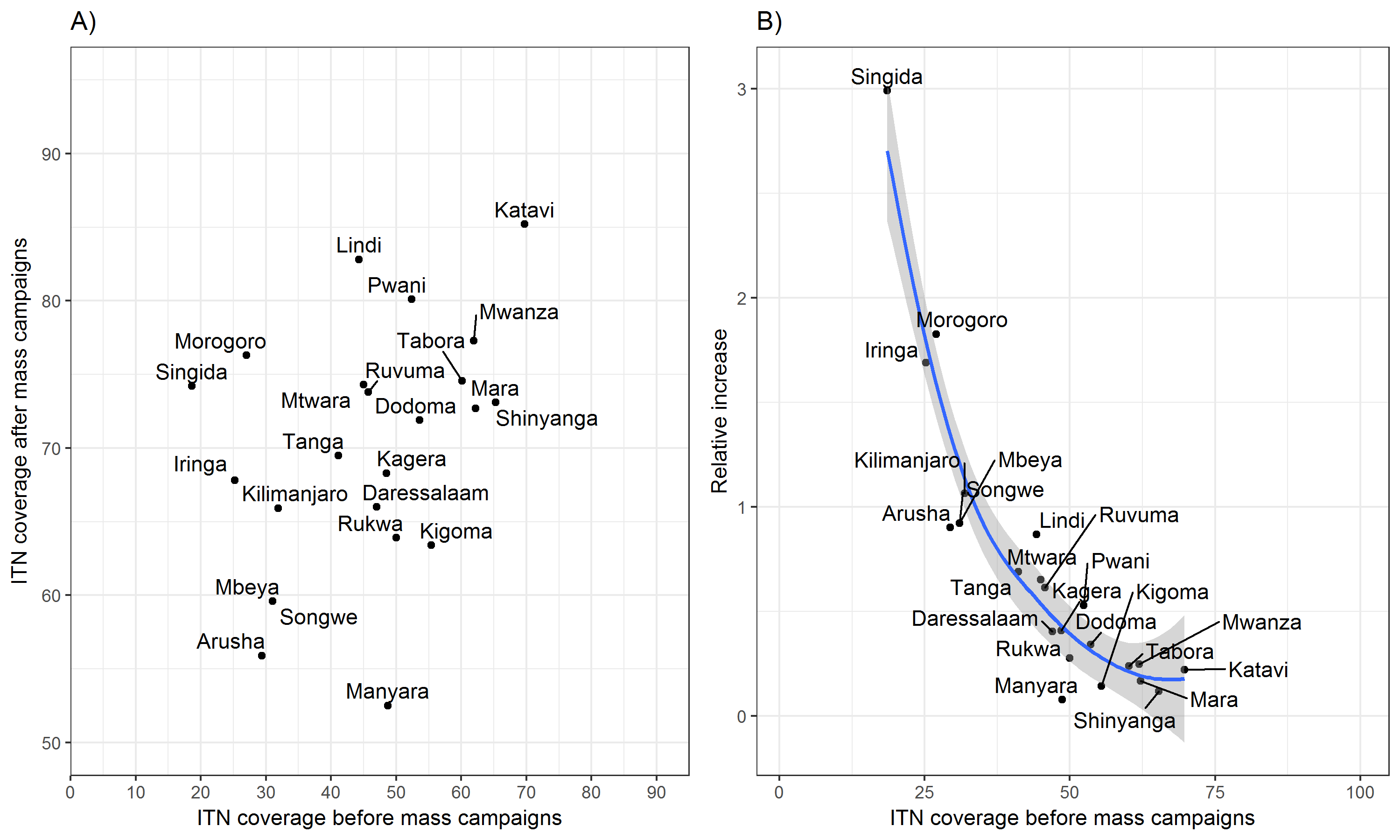


**Fig S2.7: Relation between reported ITN use before and after mass distribution campaign.**

**A)** Mean ITN usage estimates from MIS 2010 and 2012 for before mass campaigns, and MIS 2012 and 2015/16 for after mass campaigns (deployments in 2011 and 2015) per region. **B)** Mean ITN usage before mass campaign and relative increase in coverage per region with fitted loess function. Note: in both plots, only regions are shown which had the MIS survey before the ITN mass campaign in either 2012 or 2016 (n= 23, missing= 3).

The timing of the ITN deployments was assumed to be the same across all regions, but accounting for differences in deployments i.e. school net distributions in Mtwara, Lindi and Ruvuma (Table S2.3).

**Table S2.3: ITN deployment coverage per region in 2016.**

| **Campaign and survey timing** | **Adjustment method** | **Region** | **Mass campaign**  **date** | **MIS**  **2015-16**  **date** | **MIS**  **ITN use**  **2015-16 (%)** | **Adjusted ITN coverage 2016 (%)** | |
| --- | --- | --- | --- | --- | --- | --- | --- |
| Campaign before survey | Attrition of nets function to get the usage at deployment. Since the change was < 1%, the original value was kept | Geita | 01.10.2015 | 07.01.2016 | 85.5 | | |
|  |  | Kagera | 01.08.2015 | 20.01.2016 | 67.5 | | |
|  |  | Katavi | 01.07.2015 | 12.01.2016 | 85.2 | | |
|  |  | Kigoma | 01.08.2015 | 28.10.2015 | 54.3 | | |
|  |  | Mara | 01.10.2015 | 24.10.2015 | 66.7 | | |
|  |  | Mwanza | 01.08.2015 | 16.10.2015 | 68 | | |
|  |  | Shinyanga | 01.09.2015 | 10.10.2015 | 46.8 | | |
|  |  | Simiyu | 01.11.2015 | 09.01.2016 | 83.5 | | |
|  |  | Tabora | 01.07.2015 | 20.01.2016 | 75.6 | | |
| Campaign after survey | Scaled by increase ratio before/after campaign  (The scaling ratio was obtained from reported ITN usage from the DHS and MIS surveys in 2010 and 2012, in comparison to the ITN campaigns in 2009-2011) | Arusha | 01.02.2016 | 24.09.2015 | 29.2 | | 67.4 |
|  |  | Daressalaam | 24.12.2016 | 20.09.2015 | 54.4 | | 75.6 |
|  |  | Dodoma | 01.05.2016 | 28.11.2015 | 16.7 | | 70.8 |
|  |  | Iringa | 01.02.2016 | 26.11.2015 | 28 | | 68.1 |
|  |  | Kilimanjaro | 01.03.2016 | 15.09.2015 | 36.1 | | 64.5 |
|  |  | Manyara | 01.01.2016 | 28.11.2015 | 12.6 | | 65.9 |
|  |  | Mbeya | 22.11.2016 | 19.11.2015 | 26.4 | | 69.0 |
|  |  | Morogoro | 07.06.2016 | 01.12.2015 | 47.5 | | 68.7 |
|  |  | Njombe | 01.11.2015 | 20.10.2015 | 16.9 | | 70.9 |
|  |  | Pwani | 01.05.2016 | 08.01.2016 | 48.1 | | 69.3 |
|  |  | Rukwa | 01.12.2016 | 20.10.2015 | 15.1 | | 69.5 |
|  |  | Singida | 01.01.2016 | 20.01.2016 | 29.7 | | 67.1 |
|  |  | Songwe | 01.12.2016 | 03.11.2015 | 26.4 | | 69.0 |
|  |  | Tanga | 12.05.2016 | 30.11.2015 | 33.3 | | 65.3 |
| SNP before survey | Estimated in model calibration process when fitting to prevalence | Lindi |  | 30.12.2015 | 48.2 | | - |
|  |  | Mtwara |  | 06.11.2015 | 44.4 | | - |
|  |  | Ruvuma |  | 11.01.2016 | 47.8 | | - |

#### *Attrition of nets*

For nets distributed in 2012 various decay curves were simulated for councils that did not receive nets through schools. For nets distributed in 2016 and in future, a half-life of three years [36,37], following an exponential decay function as defined by Briët et al. [8] was assumed (line 4 in Fig S2.8).

**BOX 1: Smooth-compact decay function:**

$$y=\exp\frac{k-k}{(1-\left( \frac{t}{L} \right)^{2})}$$

$$t=time,$$

$$k=shape parameter \left( no dimension \right),$$

$$L= rate of decay, either the time until half decay or the time until full decay$$

$$Note: \mathrm{for}t <L, otherwise 0$$

Source: <https://github.com/SwissTPH/openmalaria/wiki/ModelDecayFunctions>


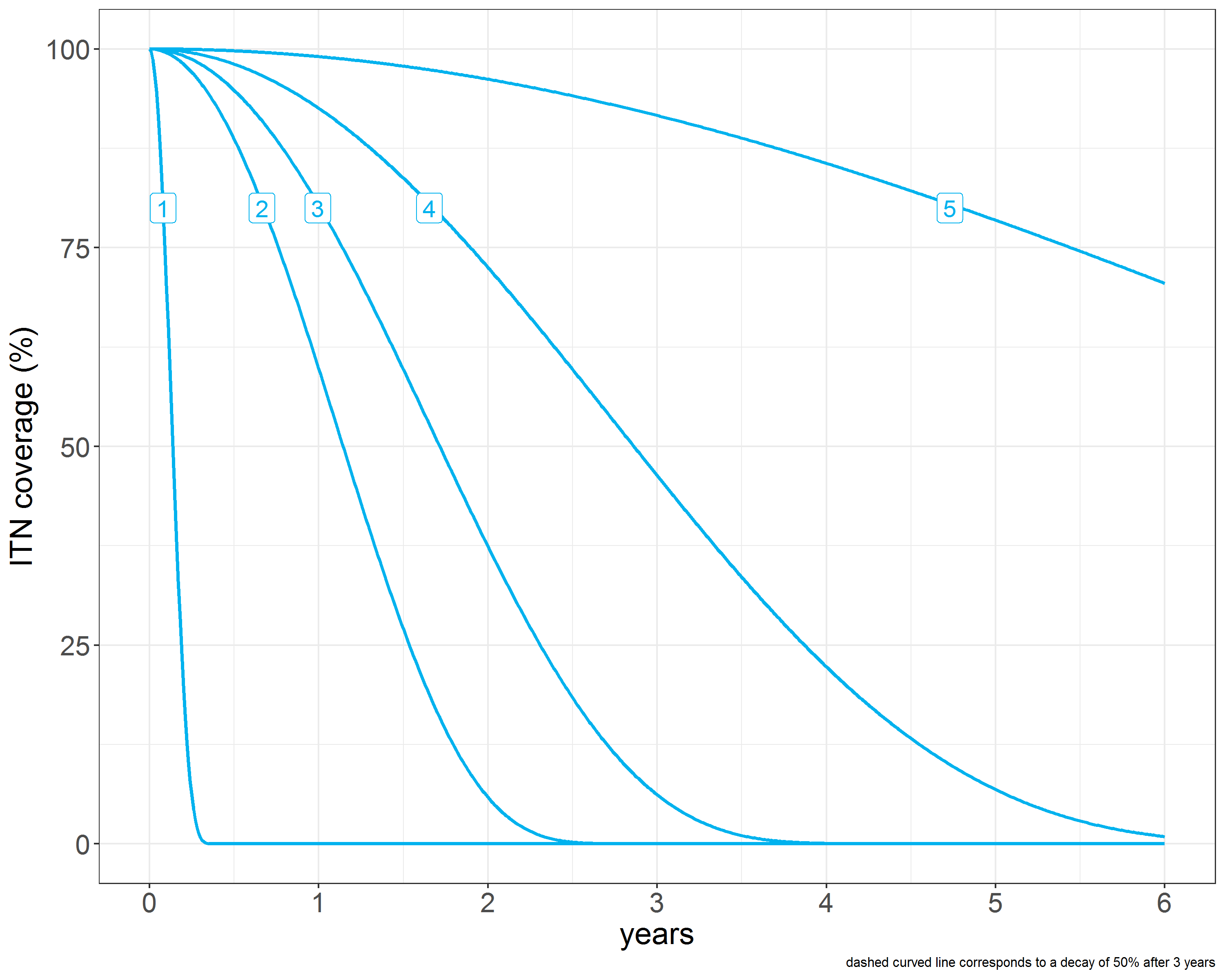


**Fig S2.8: ITN decay curves.**

### Indoor residual spraying

IRS implementation started in Mainland Tanzania in 2006/7 in the Lake Zone, targeting high prevalence areas, prone to malaria epidemics [41]. The coverage was calculated by dividing the targeted population by the total population of the council and start date of the IRS campaign used as deployment date. The maximum coverage and average timing were used for defining IRS deployments at regional level for Geita, Mara, Mwanza and Shinyanga (Fig S2.9). The IRS coverage and timing were aggregated per region, assuming the whole region had IRS.


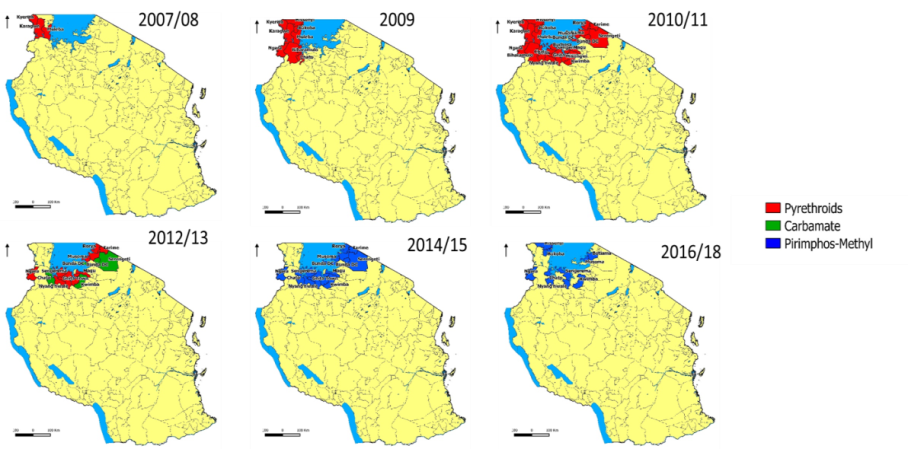


**Fig S2.9:** **Geographical distribution of IRS campaigns with various type of active ingredient between 2007 and 2018.**

Source: NMCP, reproduced with permission.

# Model calibration

The model was calibrated by fitting the simulated prevalence to the geospatial model predicted prevalence using MCMC methods and Gibbs sampler for likelihood estimation, estimating the pre-intervention EIR in 2003 and ITN usage parameter in 2012 per council. The Bayesian model was run separately for each council, with an adaptation phase of 100 iterations for one chain. The JAGS model was updated with 10’000 further iterations, and 10’000 samples were drawn from the posterior distribution, with a thinning interval of 10. Model priors were the geospatial prevalence predictions, and the likelihood functions the simulated prevalence, and the weighting parameter was estimated and applied to the estimated output parameter within the JAGS model. The outputs provided weights for each simulation which allowed reconstructing the historical trend of malaria transmission for each council. Since the predicted impact depends on the pre-intervention EIR (2003), and on the year before the deployment of future interventions in 2016 (baseline), the years 2003 and 2016 were weighted more than the years in between.

# Simulated future scenarios 2017-2020

In total six interventions were simulated as described below:

- Case management was simulated assuming an immediate increase in the effective treatment coverage to 85% via improvements in case management in 2017 with constant effectiveness.
- ITNs were simulated assuming nets treated with pyrethroids and with two different types of deployment: 1) mass replacement campaign (MRC) in January 2019, 2) continuous distributions every year, e.g. via schools net programs (SNP). The ITN coverage was set equal to the usage, assuming that all individuals who have a net are using the net [36]. The ITN parameterisation is described elsewhere [8].
- IRS was simulated assuming annual deployments in September, corresponding to the end of the dry season in most regions. The active ingredients organophosphate (Actellic 50EC) and carbamates (Bendiocarb) were rotated, starting with Bendiocarb in 2017. The effect duration was assumed to six and three months, respectively for Bendiocarb and Actellic.
- Larval source management (LSM) was simulated assuming larviciding rounds every two weeks for three months during the dry season. The effect was simulated with a constant reduction of 60% in the emergence rate of adult mosquitoes for fourteen days after deployment, irrespective of vector species.
- Mass drug administration (MDA) was simulated assuming deployment of three rounds per year for the years 2017 to 2020. The deployment rounds were in June, August and October. The effectiveness, not parameterised to field data, was simulated with immediate blood-stage clearance without protective effect. A previous MDA modelling study concluding that coverage needs to be very high for MDA to be effective [42]. However, not all in the population are eligible for MDA deployment or can be reached, and a moderate to target coverage of 80% was used.

# References

1. National Bureau of Statistics (NBS), Tanzania, Office of Chief Government Statistician (OCGS), Zanzibar. 2012 Population and Housing Census. Dar Es Salaam, Tanzania: NBS and OCGS; 2013.

2. Grover-Kopec EK, Blumenthal MB, Ceccato P, Dinku T, Omumbo JA, Connor SJ. Web-based climate information resources for malaria control in Africa. Malar J. 2006;5: 38. doi:10.1186/1475-2875-5-38

3. Ngowo HS, Kaindoa EW, Matthiopoulos J, Ferguson HM, Okumu FO. Variations in household microclimate affect outdoor-biting behaviour of malaria vectors. Wellcome Open Res. 2017;2: 102. doi:10.12688/wellcomeopenres.12928.1

4. Kisinza W, Nkya T, Msangi S, Mbilu T, Batengana B, Lyimo E, et al. Detection and Monitoring of Insecticide Resistance in Malaria Vectors in Tanzania Mainland. Dar es Salaam, Tanzania: National Institute for Medical Research; 2015.

5. Kisinza W, Tungu P, Nkya T, Malima R, Zawadi M, Msangi S, et al. Detection and Monitoring of Insecticide Resistance to Malaria Vectors in Tanzania Mainland. Dar es Salaam, Tanzania: National Institute for Medical Research; 2014.

6. Africa IRS (AIRS) Project, PMI. Tanzania end of spray report spray campaign: February 3 – April 4, 2016. Bethesda, MD: Abt Associates Inc; 2016. Available: https://www.pmi.gov/docs/default-source/default-document-library/implementing-partner-reports/tanzania-end-of-spray-report-2016-airs.pdf

7. Koenker HM, Yukich JO, Mkindi A, Mandike R, Brown N, Kilian A, et al. Analysing and recommending options for maintaining universal coverage with long-lasting insecticidal nets: the case of Tanzania in 2011. Malar J. 2013;12: 150. doi:10.1186/1475-2875-12-150

8. Briët OJ, Hardy D, Smith TA. Importance of factors determining the effective lifetime of a mass, long-lasting, insecticidal net distribution: a sensitivity analysis. Malar J. 2012;11: 20. doi:10.1186/1475-2875-11-20

9. TACAIDS, Zanzibar AIDS Commission (ZAC), National Bureau of Statistics (NBS) [Tanzania], MEASURE DHS, Macro International Inc. Tanzania HIV/AIDS and Malaria Indicator Survey 2007-08. Dar Es Salaam,Tanzania: Tanzania Commission for AIDS (TACAIDS); 2008.

10. NBS/Tanzania NB of S-, Macro ICF. Tanzania Demographic and Health Survey 2010. 2011. Available: http://dhsprogram.com/publications/publication-FR243-DHS-Final-Reports.cfm

11. TACAIDS, Zanzibar AIDS Commission (ZAC), National Bureau of Statistics (NBS) [Tanzania], Office of the Chief Government Statistician (OCGS), ICF International 2013. Tanzania HIV/AIDS and Malaria Indicator Survey 2011-12. Dar Es Salaam, Tanzania: TACAIDS, ZAC, NBS, OCGS, and ICF International; 2013.

12. MoHCDGEC, Ministry of Health (MoH) (Zanzibar), National Bureau of Statistics (NBS) [Tanzania], Office of Chief Government Statistician (OCGS), ICF International. Tanzania Demographic and Health Survey and Malaria Indicator Survey (TDHS-MIS) 2015-2016. Dar es Salaam, Tanzania and Rockville, Maryland, USA: MoHSW, MoH, NBS, OCGS, and ICF International; 2016.

13. Bhatt S, Weiss DJ, Mappin B, Dalrymple U, Cameron E, Bisanzio D, et al. Coverage and system efficiencies of insecticide-treated nets in Africa from 2000 to 2017. eLife. 2015;4: e09672. doi:10.7554/eLife.09672

14. Chitnis N, Smith T, Schapira A. Parameter Values for Transmission Model. 2010. Available: https://github.com/SwissTPH/openmalaria/wiki/XmlEntoVector

15. Chitnis N, Smith T, Steketee R. A mathematical model for the dynamics of malaria in mosquitoes feeding on a heterogeneous host population. J Biol Dyn. 2008;2: 259–285. doi:10.1080/17513750701769857

16. Kabula B, Derua YA, Tungui P, Massue DJ, Sambu E, Stanley G, et al. Malaria entomological profile in Tanzania from 1950 to 2010: a review of mosquito distribution, vectorial capacity and insecticide resistance. Tanzan J Health Res. 2011;13: 319–331.

17. Lwetoijera D, Harris C, Kiware S, Dongus S, Devine GJ, McCall PJ, et al. Effective autodissemination of pyriproxyfen to breeding sites by the exophilic malaria vector Anopheles arabiensis in semi-field settings in Tanzania. Malar J. 2014;13: 161. doi:10.1186/1475-2875-13-161

18. Kisinza WN, Nkya TE, Kabula B, Overgaard HJ, Massue DJ, Mageni Z, et al. Multiple insecticide resistance in Anopheles gambiae from Tanzania: a major concern for malaria vector control. Malar J. 2017;16: 439. doi:10.1186/s12936-017-2087-2

19. Kabula B, Tungu P, Matowo J, Kitau J, Mweya C, Emidi B, et al. Susceptibility status of malaria vectors to insecticides commonly used for malaria control in Tanzania. Trop Med Int Health. 2012;17: 742–750. doi:10.1111/j.1365-3156.2012.02986.x

20. Mwanziva CE, Kitau J, Tungu PK, Mweya CN, Mkali H, Ndege CM, et al. Transmission intensity and malaria vector population structure in Magugu, Babati District in northern Tanzania. Tanzan J Health Res. 2011;13: 54–61.

21. Killeen GF, Smith TA, Ferguson HM, Mshinda H, Abdulla S, Lengeler C, et al. Preventing Childhood Malaria in Africa by Protecting Adults from Mosquitoes with Insecticide-Treated Nets. PLoS Med. 2007;4. doi:10.1371/journal.pmed.0040229

22. Charlwood JD, Smith T, Lyimo E, Kitua AY, Masanja H, Booth M, et al. Incidence of Plasmodium falciparum infection in infants in relation to exposure to sporozoite-infected anophelines. Am J Trop Med Hyg. 1998;59: 243–251.

23. Biro S. Investigations on the Bionomics of Anopheline Vectors in the Ifakara Area (Kilombero District, Tanzania). BSBS, Basler Schnelldr. Schlattmann. 1987.

24. Kaindoa EW, Matowo NS, Ngowo HS, Mkandawile G, Mmbando A, Finda M, et al. Interventions that effectively target Anopheles funestus mosquitoes could significantly improve control of persistent malaria transmission in south–eastern Tanzania. PLoS ONE. 2017;12. doi:10.1371/journal.pone.0177807

25. Smith T, Charlwood JD, Kihonda J, Mwankusye S, Billingsley P, Meuwissen J, et al. Absence of seasonal variation in malaria parasitaemia in an area of intense seasonal transmission. Acta Trop. 1993;54: 55–72. doi:10.1016/0001-706X(93)90068-M

26. Temu EA, Minjas JN, Coetzee M, Hunt RH, Shift CJ. The role of four anopheline species (Diptera: Culicidae) in malaria transmission in coastal Tanzania. Trans R Soc Trop Med Hyg. 1998;92: 152–158.

27. Huho B, Briët O, Seyoum A, Sikaala C, Bayoh N, Gimnig J, et al. Consistently high estimates for the proportion of human exposure to malaria vector populations occurring indoors in rural Africa. Int J Epidemiol. 2013; dys214. doi:10.1093/ije/dys214

28. Chitnis N, Hardy D, Smith T. A Periodically-Forced Mathematical Model for the Seasonal Dynamics of Malaria in Mosquitoes. Bull Math Biol. 2012;74: 1098–1124. doi:10.1007/s11538-011-9710-0

29. Lengeler C, Armstrong‐Schellenberg J, D’Alessandro U, Binka F, Cattani J. Relative versus absolute risk of dying reduction after using insecticide-treated nets for malaria control in Africa. Trop Med Int Health. 1998;3: 286–290. doi:10.1046/j.1365-3156.1998.00236.x

30. Beier JC, Killeen GF, Githure JI. Short report: entomologic inoculation rates and Plasmodium falciparum malaria prevalence in Africa. Am J Trop Med Hyg. 1999;61: 109–113.

31. Penny MA, Maire N, Bever CA, Pemberton-Ross P, Briët OJT, Smith DL, et al. Distribution of malaria exposure in endemic countries in Africa considering country levels of effective treatment. Malar J. 2015;14: 384. doi:10.1186/s12936-015-0864-3

32. Stuckey EM, Smith TA, Chitnis N. Estimating malaria transmission through mathematical models. Trends Parasitol. 2013;29: 477–482. doi:10.1016/j.pt.2013.08.001

33. Tanzania Meteorological Agency. Seasonal Climatic Suitability for Malaria Transmission. [cited 9 Mar 2018]. Available: http://maproom.meteo.go.tz/maproom/Health/CSMT/index.html?resolution=.0375

34. Ministry of Health, Community Development, Gender, Elderly and Children (MoHCDGEC), Ministry of Health (MoH) (Zanzibar), National Bureau of Statistics (NBS) [Tanzania], Office of Chief Government Statistician (OCGS), ICF International. Tanzania Demographic and Health Survey and Malaria Indicator Survey (TDHS-MIS) 2015-2016. Dar es Salaam, Tanzania and Rockville, Maryland, USA: MoHSW, MoH, NBS, OCGS, and ICF International; 2016.

35. Galactionova K, Tediosi F, Savigny D de, Smith T, Tanner M. Effective Coverage and Systems Effectiveness for Malaria Case Management in Sub-Saharan African Countries. PLOS ONE. 2015;10: e0127818. doi:10.1371/journal.pone.0127818

36. Kilian A, Byamukama W, Pigeon O, Atieli F, Duchon S, Phan C. Long-term field performance of a polyester-based long-lasting insecticidal mosquito net in rural Uganda. Malar J. 2008;7: 49. doi:10.1186/1475-2875-7-49

37. WHO. Achieving and maintaining universal coverage with long-lasting insecticidal nets for malaria control. World Health Organization; 2017.

38. Malaria Atlas Project. Malaria Atlas Project. 2016 [cited 18 Aug 2016]. Available: http://www.map.ox.ac.uk

39. Tatem AJ. WorldPop, open data for spatial demography. Sci Data. 2017;4: 170004. doi:10.1038/sdata.2017.4

40. Lalji S, Ngondi JM, Thawer NG, Tembo A, Mandike R, Mohamed A, et al. School Distribution as Keep-Up Strategy to Maintain Universal Coverage of Long-Lasting Insecticidal Nets: Implementation and Results of a Program in Southern Tanzania. Glob Health Sci Pract. 2016;4: 251–263. doi:10.9745/GHSP-D-16-00040

41. RTI International. Tanzania Vector Control Scale-up Project: Spray Performance Report. November 2011–May 2012. RTI International; 2012. Available: https://www.pmi.gov/docs/default-source/default-document-library/implementing-partner-reports/tanzania-vector-control-scale-up-project-spray-performance-report-november-2011-may-2012.pdf?sfvrsn=5

42. Pemberton-Ross P, Chitnis N, Pothin E, Smith TA. A stochastic model for the probability of malaria extinction by mass drug administration. Malar J. 2017;16. doi:10.1186/s12936-017-2010-x
