# Supplementary material for: Simulating the council-specific impact of anti-malaria interventions: A tool to support malaria strategic planning in Tanzania
Source: PLoS One. 2020 Feb 19;15(2):e0228469. doi: 10.1371/journal.pone.0228469 (PMC7029840; doi:10.1371/journal.pone.0228469)
Supplement: S3 File — (DOCX) [file pone.0228469.s003.docx]

# S3 File: Outcome and cost calculations

The model predicted outcomes were further processed, calculating outcome measures, such as prevalence or cases averted and attaching costs to the simulated intervention scenarios per council (Table S3.1).

**Table S3.1: Parameter and abbreviations used.**

| Symbol | Name | Unit /description |
| --- | --- | --- |
| $t$ | Time | Years |
| $j$ | Setting | Councils |
| $k$ | Age group | 0-5 years; 2-10 years, 0-99 years (total population) |
| $l$ | Intervention scenario | Future interventions (single or combined) |
| **Abbreviations** | **Full name** | **Description** |
| $c$ | Counterfactual | Discontinuation of all interventions apart from case management running at current levels, as in 2016 |
| $b$ | Baseline | Baseline year 2016 |
| $U$ | Uncomplicated | Uncomplicated malaria cases |
| $S$ | Severe | Severe malaria cases |
| $PopPerNet$ | Population per net | Number of people sleeping under the same net. |
| $Pop.perHH$ | Populationper household | Number of people living in the same household. |
| ***OpenMalaria* output*** | **Description** | |
| $nPatent$ | The number of human hosts whose total (blood-stage) parasite density is above the detection threshold. | |
| $nHost$ | Total number of humans. | |
| $nUncomplicated$ | Number of episodes (uncomplicated). An episode of uncomplicated malaria is a period during which an individual has symptoms caused by malaria parasites present at the time of illness, where the symptoms do not qualify as severe malaria. […] | |
| $nSevere$ | Number of episodes (severe).An episode of severe malaria is a period during which an individual has symptoms, qualifying as severe malaria, caused by malaria parasites present at the time of illness. […] | |

*) <https://github.com/SwissTPH/openmalaria/wiki/XmlMonitoring>

**Table S3.2: Calculated outcome measures from OpenMalaria monitoring measures**

| Outcome | Description | Calculation |
| --- | --- | --- |
| Prevalence | Percentageof patent infections above the detection limit (200 parasites/ul blood) out of the total population | ${Pf\mathrm{PR}}_{jklt} =\left( \frac{{nPatent}_{jklt}}{{nHost}_{jklt}} \right)$ |
| Incidence | The number of all symptomatic malaria infections per 1000 population, regardless of health seeking behaviour. | ${Incidence}_{jklt}=\left( \frac{{nUncomplicated}_{jklt}+{nSevere}_{jklt}}{{nHost}_{k}} \right)*1000$ |
| Malaria cases | The number of all symptomatic malaria infections per council population, regardless of health seeking behaviour. | ${Cases.U}_{jklt}=\left( \frac{{nUncomplicated}_{jklt}}{{nHost}_{k}} \right)*{Population}_{2016}$  ${Cases.S}_{jklt}=\left( \frac{{nSevere}_{jklt}}{{nHost}_{k}} \right)*{Population}_{2016}$  ${Cases}_{jklt}=\left( \frac{{nUncomplicated}_{jklt}+{nSevere}_{jklt}}{{nHost}_{k}} \right)*{Population}_{2016}$ |
| Malaria cases averted | The number of malaria cases averted by intervention compared to the counterfactual. | ${Cases.Averted.U}_{jklt}= {Cases.U}_{jkct}- {Cases.U}_{jklt}$  ${Cases.Averted.S}_{jklt}= {Cases.S}_{jkct}- {Cases.S}_{jklt}$  ${Cases.Averted}_{jklt}= {Cases}_{jkct}- {Cases}_{jklt}$ |
| Relative reduction in malaria cases | The number of malaria cases averted by intervention compared to the counterfactual | ${Cases.red.perc}_{jklt}=\left( \frac{\left( {Cases}_{jkct}- {Cases}_{jklt} \right)}{{Cases}_{tjkc}} \right)*100$ |

**Costs estimates**

All intervention costs were costs per person in USD (Table S2.6). The numbers of rounds of an intervention were per year and had the values “0” for no intervention deployment and “1” for intervention deployment. The cost calculation for school net distribution took into account that with time more nets need to be distributed to maintain a certain coverage level in the population. The annual total costs were calculated per council, considering the council population of 2016. The costs of each scenario were summed over the years 2017-2020 and interventions in that strategy. Three types of cost were generated per scenario: total net costs, total costs, and total intervention costs (excluding CM). The treatment savings (treatment costs from cases averted) were subtracted from the total net cost (Table S2.7).

**Table S3.3: Unit costs per intervention.**

|  | Cost | Unit | Source |
| --- | --- | --- | --- |
| CM | $2.1 (Uncomp)  $49.4 (Severe) | Per episode | [1] |
| MRC | $4.00 | Per net,  (~ 2 people per net) | Personal communication with NMCP |
| SNP | $3.11 |  |  |
| IRS | $30 | Per structure  (~5 people per hh*) | [2] |
| MDA | $5.95 | Cost per drug  (= cost per person) | [3,4] |
| LSM | $4.32 | Per population per year (3 rounds a year) | [5] and personal communication |

*) Council specific based on data from the population census [6].

**Table S3.4: Cost calculations.**

| Type | Calculation |
| --- | --- |
| Case management | ${CMcost.U}_{jlt}=\left( {Cases.U}_{jlt}*{TreatmentCoverage}_{jlt} \right)*Treatment.cost.U$  ${CMcost.S}_{jlt}=\left( {Cases.S}_{jlt}*{TreatmentCoverage}_{jlt} \right)*Treatment.cost.S$  ${CMcost}_{jlt}={CMcost.U}_{jlt}+ {CMcost.S}_{jlt}$ |
| Treatment savings | ${TreatmentSavings.U}_{jlt}=\left( {CasesAverted.U}_{jlt}*{TreatmentCoverage}_{jlt} \right)*Treatment.cost.U$  ${TreatmentSavings.S}_{jlt}=\left( {CasesAverted.S}_{jlt}*{TreatmentCoverage}_{jlt} \right)*Treatment.cost.S$  ${TreatmentSavings}_{jlt}={TreatmentSavings.U}_{jlt}+ {TreatmentSavings.S}_{jlt}$ |
| ITN | ${ITNcost.MRC}_{jlt}=\left( \left( \frac{{Population}_{2016}}{PopPerNet} \right) *{ITNcov.MRC}_{jlt}*{MRCrounds}_{jlt} \right)*ITN.cost.MRC$  ${ITNcost.SNP}_{jlt}=\left( \left( \left( \frac{{Population}_{2016}}{PopPerNet} \right)*{attrition}_{k}*{ITNcov.SNP}_{jlt} \right)*{SNProunds}_{jlt} \right)*ITN.cost.SNP$  ${ITNcost}_{jlt}={ITNcost.MRC}_{jlt}+ {ITNcost.SNP}_{jlt}$ |
| IRS | ${IRScosts}_{jlt}=\left( \left( \frac{{Population}_{2016}*{IRScoverage}_{jlt}}{Pop.perHH} \right)*{IRSrounds}_{jlt} \right)*IRScost$ |
| LSM | ${LSMcosts}_{jlt}=\left( \left( {Population}_{2016}*{LSMcoverage}_{jlt} \right)*{LSMrounds}_{jlt} \right)*LSMcost$ |
| Total costs | ${Total costs}_{jlt}= {CMcosts}_{jlt}+{ITNcosts}_{jlt}+{IRScosts}_{jlt}+{LSMcosts}_{jlt}+{MDAcosts}_{jlt}$ |
| Total net costs | ${Total net costs}_{jlt}= {Total costs}_{jlt}- {TreatmentSavings}_{jlt}$ |
| Intervention costs | ${Intervention costs}_{jlt}= {Total costs}_{jlt}- {CMcosts}_{jlt}$ |

**References**

1. Galactionova K, Tediosi F, Camponovo F, Smith TA, Gething PW, Penny MA. Country specific predictions of the cost-effectiveness of malaria vaccine RTS,S/AS01 in endemic Africa. Vaccine. 2017;35: 53–60. doi:10.1016/j.vaccine.2016.11.042

2. Africa IRS (AIRS) Project, PMI. Tanzania end of spray report spray campaign: February 3 – April 4, 2016. Bethesda, MD: Abt Associates Inc; 2016. Available: https://www.pmi.gov/docs/default-source/default-document-library/implementing-partner-reports/tanzania-end-of-spray-report-2016-airs.pdf

3. Walker PGT, Griffin JT, Ferguson NM, Ghani AC. Estimating the most efficient allocation of interventions to achieve reductions in Plasmodium falciparum malaria burden and transmission in Africa: a modelling study. Lancet Glob Health. 2016;4: e474–e484. doi:10.1016/S2214-109X(16)30073-0

4. Scott N, Hussain SA, Martin-Hughes R, Fowkes FJI, Kerr CC, Pearson R, et al. Maximizing the impact of malaria funding through allocative efficiency: using the right interventions in the right locations. Malar J. 2017;16. doi:10.1186/s12936-017-2019-1

5. Rahman R, Lesser A, Mboera L, Kramer R. Cost of microbial larviciding for malaria control in rural Tanzania. Trop Med Int Health. 2016;21: 1468–1475. doi:10.1111/tmi.12767

6. National Bureau of Statistics (NBS), Tanzania, Office of Chief Government Statistician (OCGS), Zanzibar. 2012 Population and Housing Census. Dar Es Salaam, Tanzania: NBS and OCGS; 2013.
