# Supplementary material for: Simulating the council-specific impact of anti-malaria interventions: A tool to support malaria strategic planning in Tanzania
Source: PLoS One. 2020 Feb 19;15(2):e0228469. doi: 10.1371/journal.pone.0228469 (PMC7029840; doi:10.1371/journal.pone.0228469)
Supplement: S4 File — The file includes a description of the estimated EIR in comparison to reported EIR obtained from literature, and figures of the sub-national fitting performance. It further includes the predicted intervention impact (prevalence and cases) per region and strategy, as well as population-weighted versus unweighted estimates. (DOCX) [file pone.0228469.s004.docx]

**S4-file: Additional results**

Contents

[1 Estimated pre-intervention EIR 2](#_Toc22294585)

[1.1 Comparison of estimated and observed pre-intervention EIRs 2](#_Toc22294586)

[2 Fitting performance at sub-national level 5](#_Toc22294587)

[2.1 Pre-intervention prevalence 2003 6](#_Toc22294588)

[2.2 Baseline prevalence in 2016 7](#_Toc22294589)

[2.3 Historical trend 7](#_Toc22294590)

[3 Comparison of impact per intervention strategies per region 8](#_Toc22294591)

[4 Population weighed and unweighted mean prevalence 12](#_Toc22294592)

[References 13](#_Toc22294593)

# Estimated pre-intervention EIR

The estimated council-specific pre-intervention EIR reflects the trend in geospatial predicted prevalence used for fitting, shown in the main manuscript Fig 2 and in Fig S1.2. The geographical pattern is similar to previously published prevalence risk maps [1–3], and climate factors [4] (Fig S4.1).


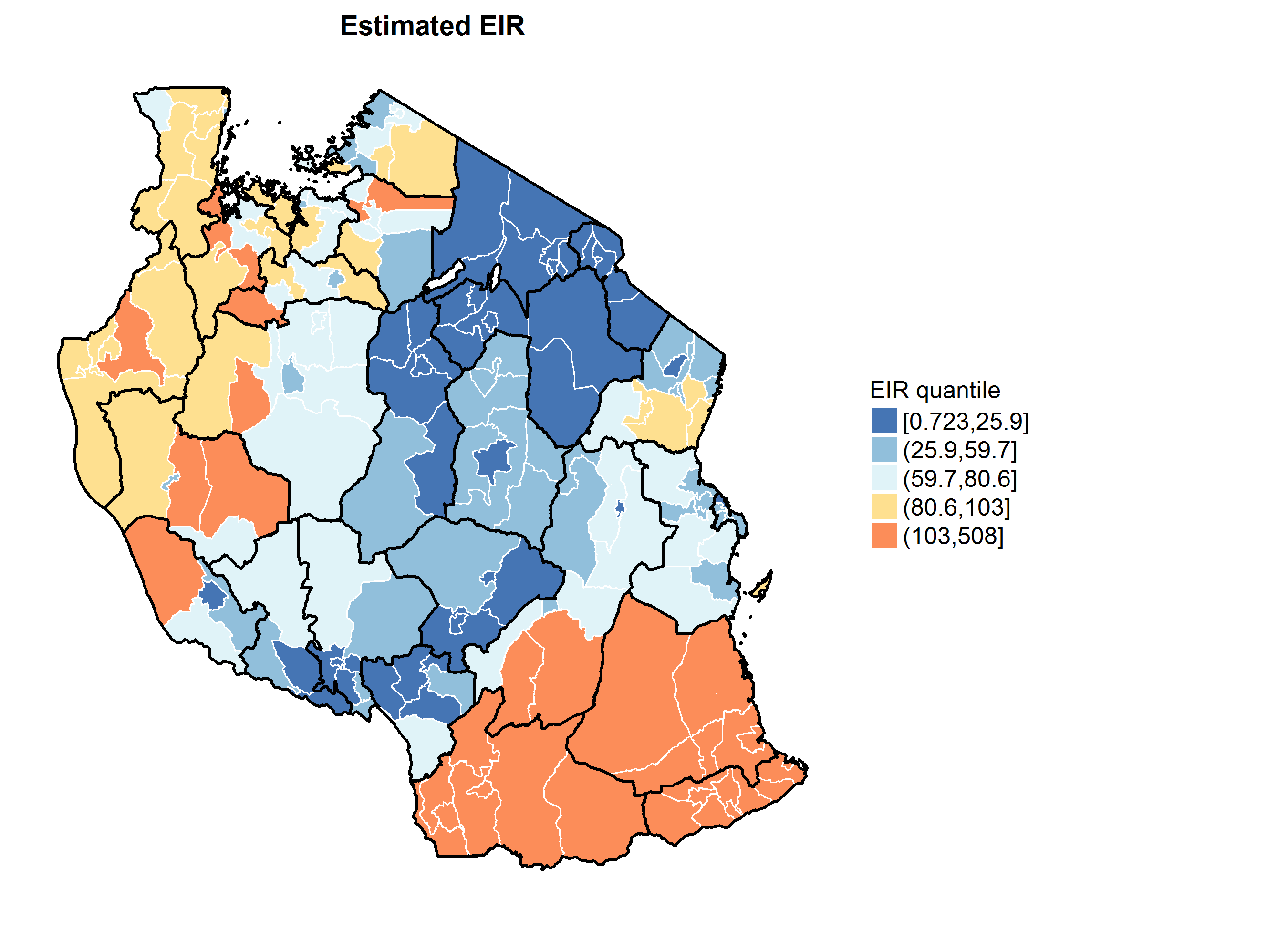


**Fig S4.1. Map of the estimated pre-intervention EIR per council.**

## Comparison of estimated and observed pre-intervention EIRs

The validation of estimates of EIR is challenging due to high variations in entomological outcomes, which highly depend on the local environment, often seasonality and vector species. A previously literature review on entomological studies in Tanzania was used to extract annual EIR values between 1990 and 2005 (Dr Fredros Okomu personal communication). The identified studies were compared with the database from Massey et al. [5], to ensure most relevant studies were captured, and 15 unique studies were identified, reporting EIR estimates for 53 study sites in 14 councils from four regions (Table S3.1). Most of the study sites were located in Kilombero and Ulanga (17 and 12 study sites respectively), while in 6 councils only one study site was included. Some studies reported the EIR per vector species [6]; in that case, the total annual EIR was calculated by summing the annual EIR per species, and some studies sites reported daily EIR, in which case annual EIRs were projected, as in [7]. The limited available data did not permit a proper validation, and a simple comparison of the reported EIR with the model-estimated EIR was done. The single annual EIR estimates for all species were compared with the mean, median and credible intervals of the estimated EIR. Overall, the comparison showed high variability in both estimated and reported values (Fig S3.2). Possible reasons for large discrepancies could be ecological factors of the study sites since heterogeneity within councils was not accounted for in the modelling. For example, in Bagamoyo, Shiff et al. conducted a study to assess the relationship between transmission and altitude [8]; hence the EIR values show high variation due to different elevation of the study sites included. The EIR estimation for rural Moshi was extremely far from the observed values, which could be due to differences in study sites of the prevalence studies used for the geospatial model used to estimate the EIR, and the sites included in the study from Ijumba et al., which was all located in different agro-ecosystems [9]. The comparison between reported annual EIR and the estimated EIR, based on the geo-spatial prevalence predictions (S1 file), is also limited, as the relationship between prevalence and EIR is not linear [10]. For high levels of EIR, prevalence is expected to have little variations. In OpenMalaria the simulations are plateauing around a prevalence of 80% prevalence and an EIR of 100 ibpa [11], and for high transmission the trends rather than the actual EIR need to correspond to each other, as in Tanga, estimated EIRs of >80 correspond to reported EIRs of > 200 ibpa and above.


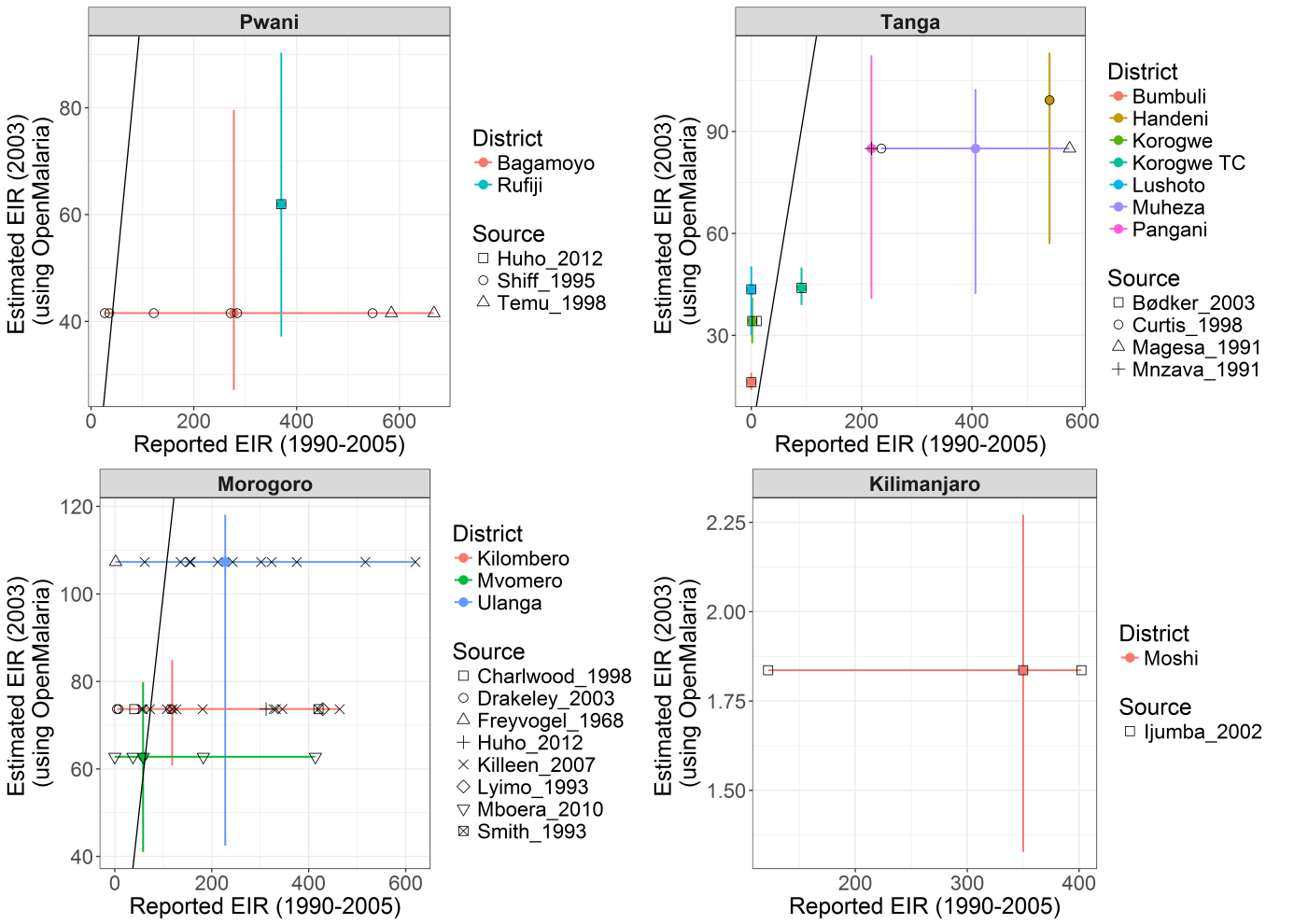


**Fig S4.2. Comparison of estimated and observed pre-intervention EIR around the year 2000 (1990-2005).** N=11 councils. Points show EIR values reported per study site per council and source^[[1]](#footnote-1)^. Vertical error bars credible intervals, and horizontal error bars represent minimum and maximum values aggregated per council (no horizontal error bars if there was only one study site per council). The black line shows perfect correspondence.

**Table S4.1: Comparison of estimated and observed pre-intervention EIR around the year 2000 (1990-2005)**

| **Region** | **Council** | **Estimated annual EIR  based on model predictions*** | | | | **Reported**  **annual EIR** | **Study site** | **Source** |
| --- | --- | --- | --- | --- | --- | --- | --- | --- |
|  |  | **mean** | **median** | **q2.5** | **q97.5** |  |  |  |
| Kilimanjaro | Moshi | 1.83 | 1.84 | 1.33 | 2.27 | 122 | Chekereni | [9] |
| Kilimanjaro | Moshi | 1.83 | 1.84 | 1.33 | 2.27 | 350 | Kisangasangeni | [9] |
| Kilimanjaro | Moshi | 1.83 | 1.84 | 1.33 | 2.27 | 402 | Mvuleni | [9] |
| Morogoro | Kilombero | 73.53 | 73.68 | 60.83 | 84.88 | 40.2 | Idete | [12] |
| Morogoro | Kilombero | 73.53 | 73.68 | 60.83 | 84.88 | 4.4 | Ifakara | [13] |
| Morogoro | Kilombero | 73.53 | 73.68 | 60.83 | 84.88 | 114.4 | Ifakara | [13] |
| Morogoro | Kilombero | 73.53 | 73.68 | 60.83 | 84.88 | 7.6 | Ifakara | [13] |
| Morogoro | Kilombero | 73.53 | 73.68 | 60.83 | 84.88 | 312 | Ulanga/Kilombero | [6] |
| Morogoro | Kilombero | 73.53 | 73.68 | 60.83 | 84.88 | 54.65-463.74 | 11 villages | [14]* |
| Morogoro | Kilombero | 73.53 | 73.68 | 60.83 | 84.88 | 126.85 | Itongoa A | [14] |
| Morogoro | Kilombero | 73.53 | 73.68 | 60.83 | 84.88 | 428.8 | Michenga | [15] |
| Morogoro | Kilombero | 73.53 | 73.68 | 60.83 | 84.88 | 420 | Namawala | [15] |
| Morogoro | Ulanga | 100.1 | 107.3 | 42.48 | 118.12 | 1.51 | Matanila-Chindwangi | [16] |
| Morogoro | Ulanga | 100.1 | 107.3 | 42.48 | 118.12 | 61.68 - 620.02 | 11 villages | [14]* |
| Morogoro | Mvomero | 62.16 | 62.77 | 41.03 | 79.86 | 58.4 | Mkindo | [17] |
| Morogoro | Mvomero | 62.16 | 62.77 | 41.03 | 79.86 | 182.5 | Mkindo | [17] |
| Morogoro | Mvomero | 62.16 | 62.77 | 41.03 | 79.86 | 37.5 | Luhindo | [17] |
| Morogoro | Mvomero | 62.16 | 62.77 | 41.03 | 79.86 | 0 | Dakawa | [17] |
| Morogoro | Mvomero | 62.16 | 62.77 | 41.03 | 79.86 | 413.5 | Mtibwa | [17] |
| Pwani | Rufiji | 61.73 | 61.94 | 37.15 | 90.32 | 370 | Rufiji | [6] |
| Pwani | Bagamoyo | 45.34 | 41.56 | 27.15 | 79.59 | 284 | Matimbwa | [8] |
| Pwani | Bagamoyo | 45.34 | 41.56 | 27.15 | 79.59 | 26.7 | Chasimba | [8] |
| Pwani | Bagamoyo | 45.34 | 41.56 | 27.15 | 79.59 | 547.5 | Zinga | [8] |
| Pwani | Bagamoyo | 45.34 | 41.56 | 27.15 | 79.59 | 271.6 | Mapinga | [8] |
| Pwani | Bagamoyo | 45.34 | 41.56 | 27.15 | 79.59 | 122.1 | Yombo | [8] |
| Pwani | Bagamoyo | 45.34 | 41.56 | 27.15 | 79.59 | 35.5 | Kongo | [8] |
| Pwani | Bagamoyo | 45.34 | 41.56 | 27.15 | 79.59 | 584 | Kongo | [18] |
| Pwani | Bagamoyo | 45.34 | 41.56 | 27.15 | 79.59 | 667 | Matimbwa | [18] |
| Tanga | Bumbuli | 16.31 | 16.24 | 13.93 | 19 | 0.08 | Balangai | [19] |
| Tanga | Korogwe | 34.33 | 34.25 | 27.68 | 41 | 9.7 | Magundi | [19] |
| Tanga | Korogwe | 34.33 | 34.25 | 27.68 | 41 | 1.7 | Kwamhanya | [19] |
| Tanga | Korogwe | 34.33 | 34.25 | 27.68 | 41 | 1.8 | Bagamoyo | [19] |
| Tanga | Korogwe TC | 44.09 | 43.99 | 38.93 | 49.98 | 91 | Kwameta | [19] |
| Tanga | Lushoto | 42.45 | 43.56 | 30 | 50.31 | 0.03 | Milungui | [19] |
| Tanga | Handeni | 95.29 | 99.25 | 56.95 | 113.22 | 540.2 | Hale | [7] |
| Tanga | Muheza | 81.24 | 85.02 | 42.18 | 102.52 | 235.6 | Temgini and Enzi | [7] |
| Tanga | Muheza | 81.24 | 85.02 | 42.18 | 102.52 | 576.7 | Kumbamtoni | [20] |
| Tanga | Pangani | 82.7 | 85.03 | 40.76 | 112.45 | 217.7 | Kikwazu | [21] |
| *) Unpublished data and EIR values per village not shown. | | | | | | | | |

# Fitting performance at sub-national level

The calibration of the model at council level used as much as possible local data and complementary information at a lower resolution when needed. Only two parameters were fitted to ensure adequation with historical malaria trends between 2003 and 2016. The figures below show the fitting of the baseline prevalence in 2003 and in 2016 per council grouped by region (Fig S4.3 and Fig S4.4) and, and the historical prevalence over time by region (Fig S4.5).

## Pre-intervention prevalence 2003


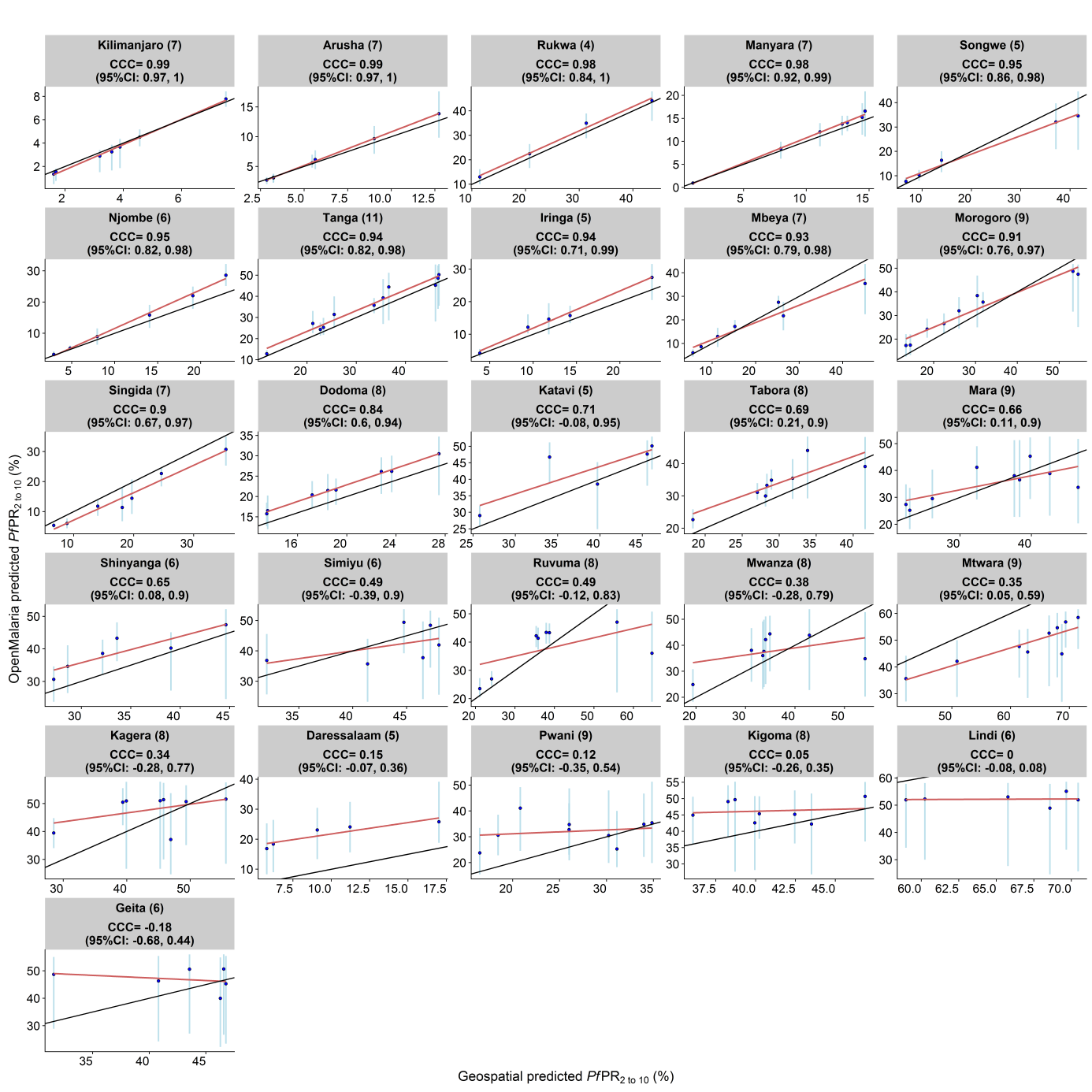


**Fig S4.3. Fitting per 2003 prevalence per council, grouped by region.**

The facets show each region with the number of councils in the brackets. Each point represents one council within the region. The scatter plots (right) shows the respective prevalence estimates (points), with the regression line (blue line), and perfect correspondence line (black line). CCC = Lin’s concordance correlation coefficients [22].

## Baseline prevalence in 2016


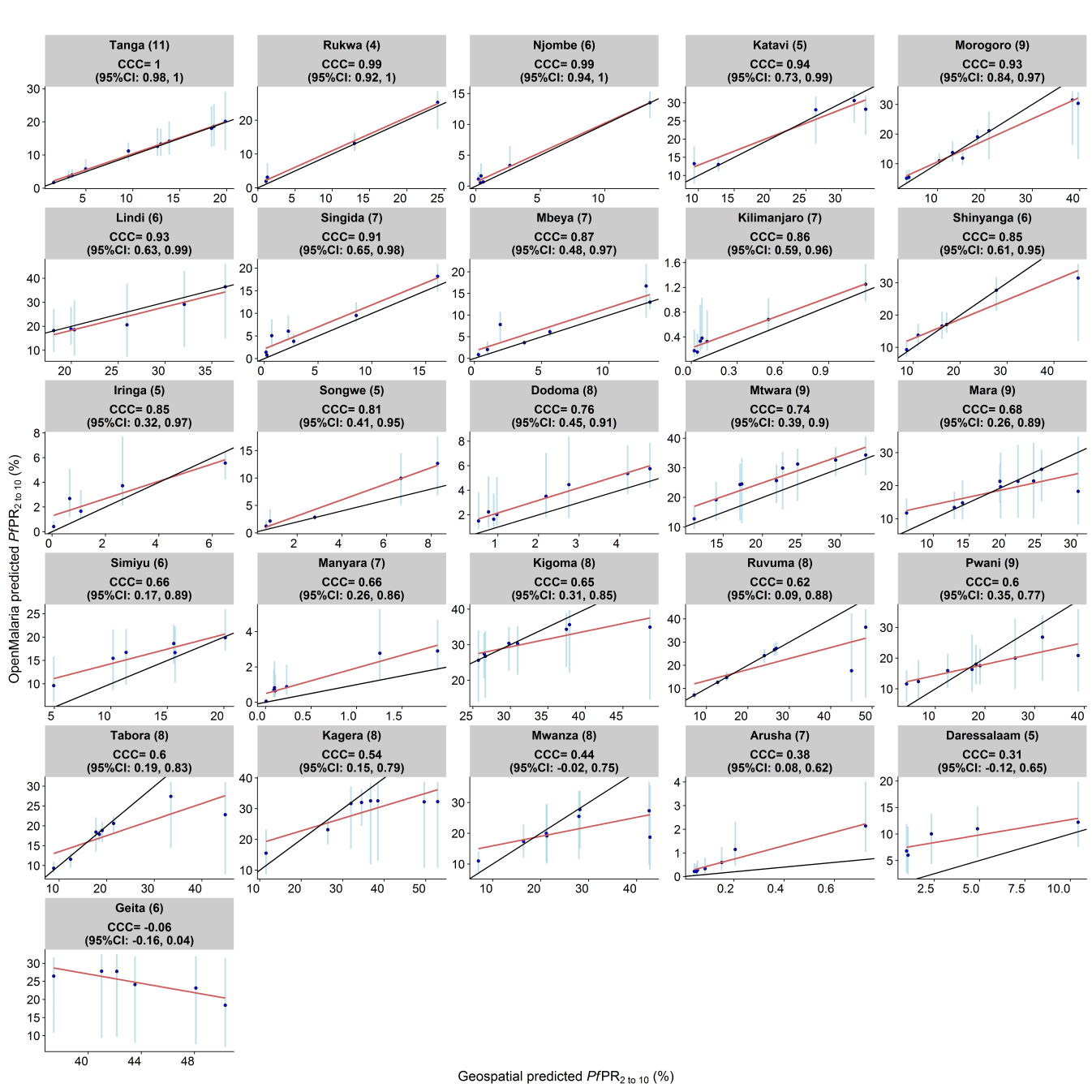


**Fig S4.4.** **Fitting per 2016 prevalence per council, grouped by region**.

The facets show each region with the number of councils in the brackets. Each point represents one council within the region. The scatter plots (right) shows the respective prevalence estimates (points), with the regression line (blue line), and perfect correspondence line (black line). CCC = Lin’s concordance correlation coefficients [22].

## Historical trend

The ITN coverage was assumed to be known and therefore fixed (see S2 File), and in some regions and councils, the reduction in prevalence did not correspond to the increase in coverage over time. It was assumed that the pre-intervention EIR and the baseline year before future intervention deployment would be the most relevant time points for the model to reproduce.


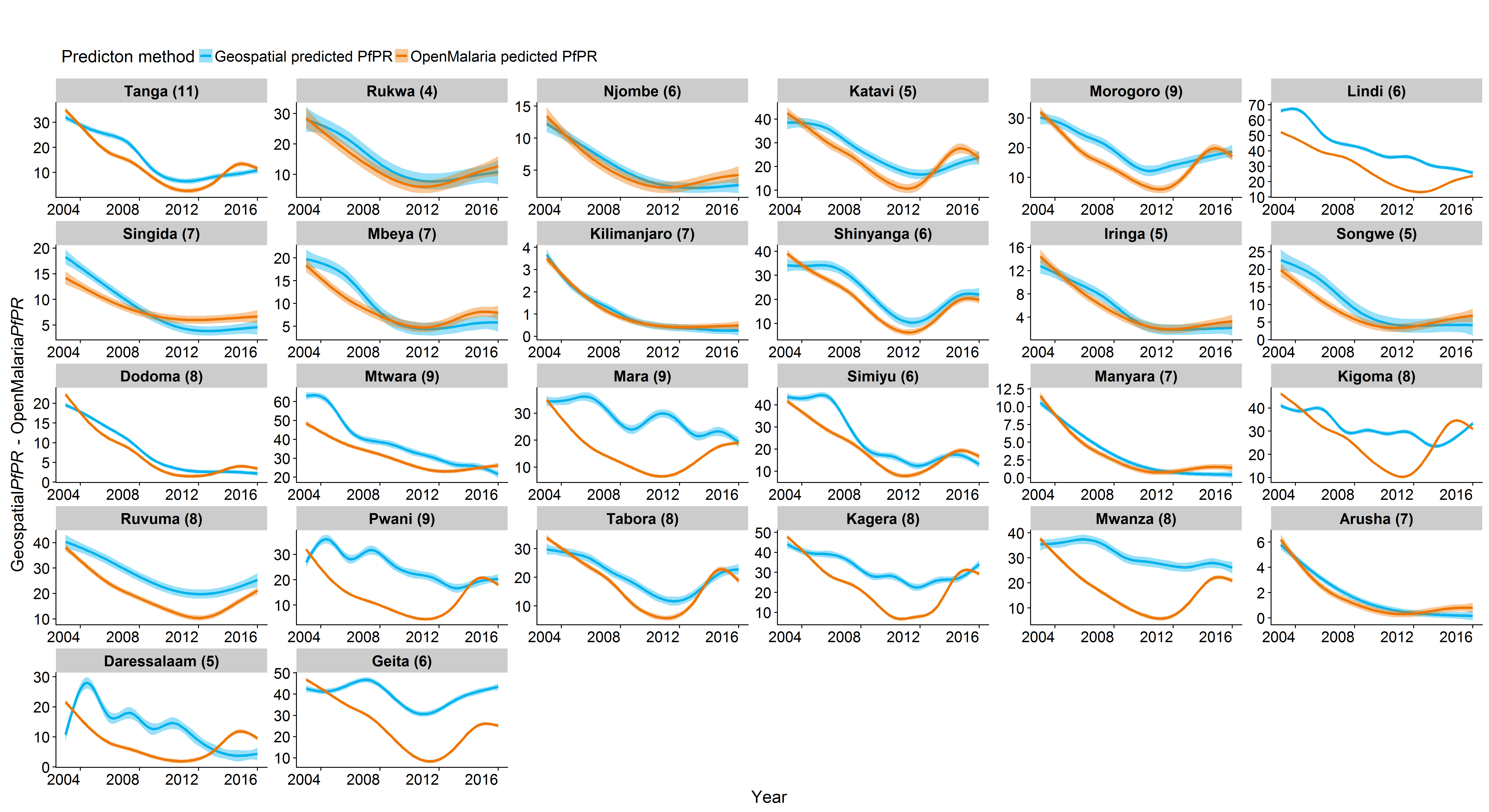


**Fig S4.5.** **Reproduced historical trend aggregated per region.**

The prevalence estimates per council (n=184) were aggregated per region (n=26). The number in brackets shows the number of councils within a region and the facets was sorted by the fitting performance, as shown in Fig 4.4.

# Comparison of impact per intervention strategies per region

Three strategies were used for stratification and the choice of interventions. For each council, the choice of the interventions was defined as (1) the interventions that comply with the current NMSP a) without or b) with an improvement of the case management, (2) the interventions that form the most cost-effective strategy or (3) the interventions that lead to the national target with the smallest resources. These strategies were compared to the counterfactual scenario, which was defined as the discontinuation of vector control and with maintained case management at current levels in 2016 (see main manuscript).

The predictions of the 184 councils were aggregated per region for simplicity and visualisation of sub-national heterogeneity. The regional mean predictions for prevalence as well as for cases averted are shown in Fig S4.6-8. Overall, the counterfactual scenario led to an increase in prevalence everywhere, but at different speed and to different levels. The rebound depends on the pre-intervention transmission intensity and effectiveness of historical interventions (not shown). The most cost-effective strategy lies between both the current NMSP, with and without improved case management and the strategy leading to the national target was the most impactful strategy in terms of prevalence and cases averted. The order of the strategies varied substantially in very low regions, namely in Arusha, Kilimanjaro, and Manyara, most likely due to stochastic and model uncertainty at such low transmission levels. The most cost-effective strategy was predicted to avert more cases and to reach lower prevalences than both the NMSP strategies in some regions but not in others and the differences between the two current NMSPs, with maintained and with improved case management, varied among the regions.

Those variations were likely due to differences in setting specific increase in case management levels, varying from 40% to 21% increase among the regions. Furthermore, region-specific differences may arise when interventions, which were part of the current NMSP, were discontinued in the other strategies. For instance, IRS was part of the current NMSP in the Lake Zone but was predicted not to be cost-effective and discontinued. Comparing the current NMSP with the most cost-effective strategy in those regions is different from the other regions which did not have IRS in the past. Another example would be the discontinuation of ITNs in low transmission regions, in Arusha, Kilimanjaro and Manyara (main manuscript Fig 6). The sub-national comparison highlights the heterogeneity and the importance to consider the historical trends, differences in seasonality and entomology when predicting likely impact of future intervention scenarios at local level and the importance to select proper outcome measures depending on transmission intensity.


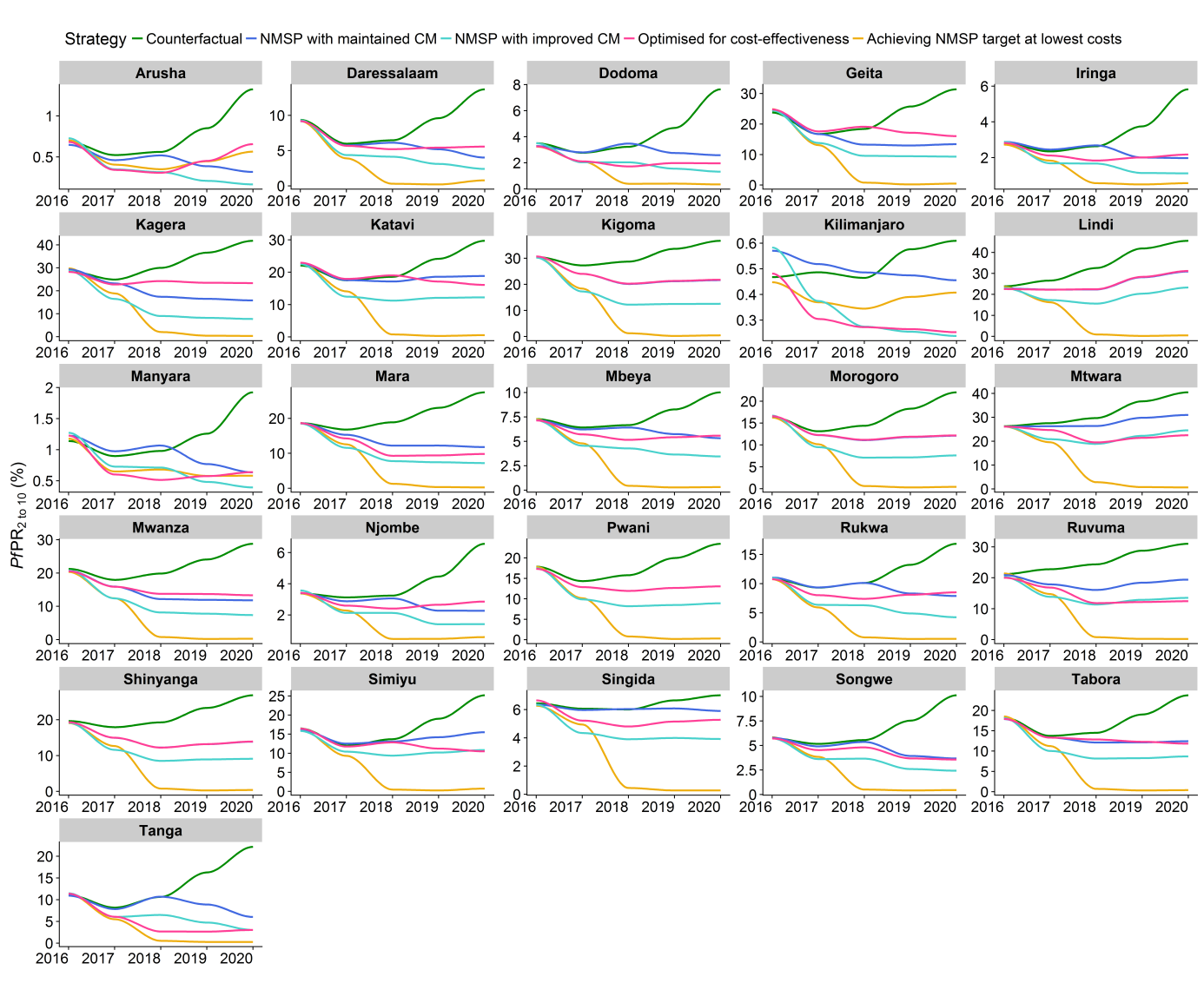


**Fig S4.6. Predicted prevalence over time per strategy and region.**

Solid lines show the aggregated mean of council predictions. Confidence intervals showed high overlap and were removed.


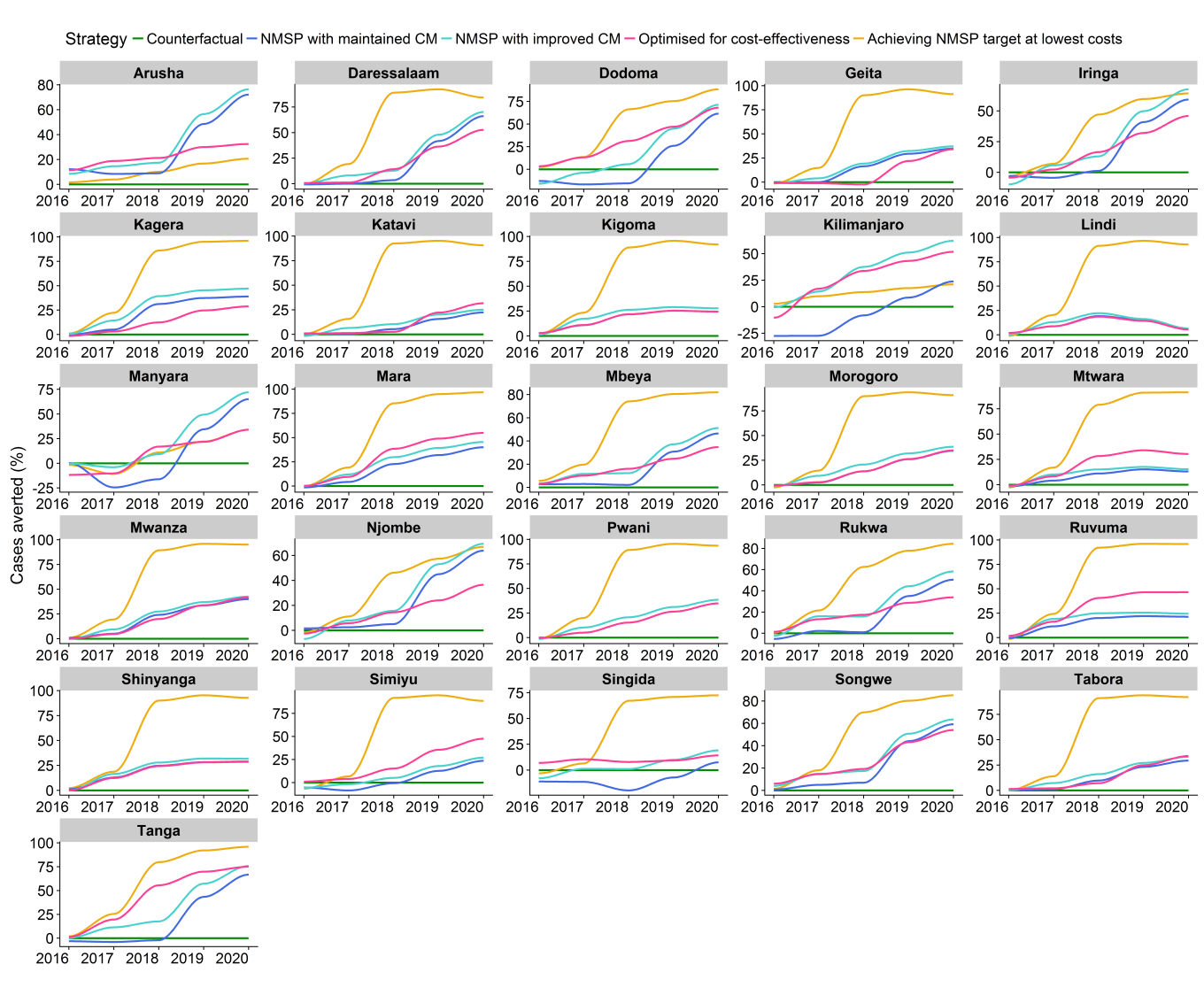
**Fig S4.7: Reduction in cases (%) over time per strategy and region.**

Solid lines show the aggregated mean of council predictions. The confidence intervals showed high overlap and were removed.


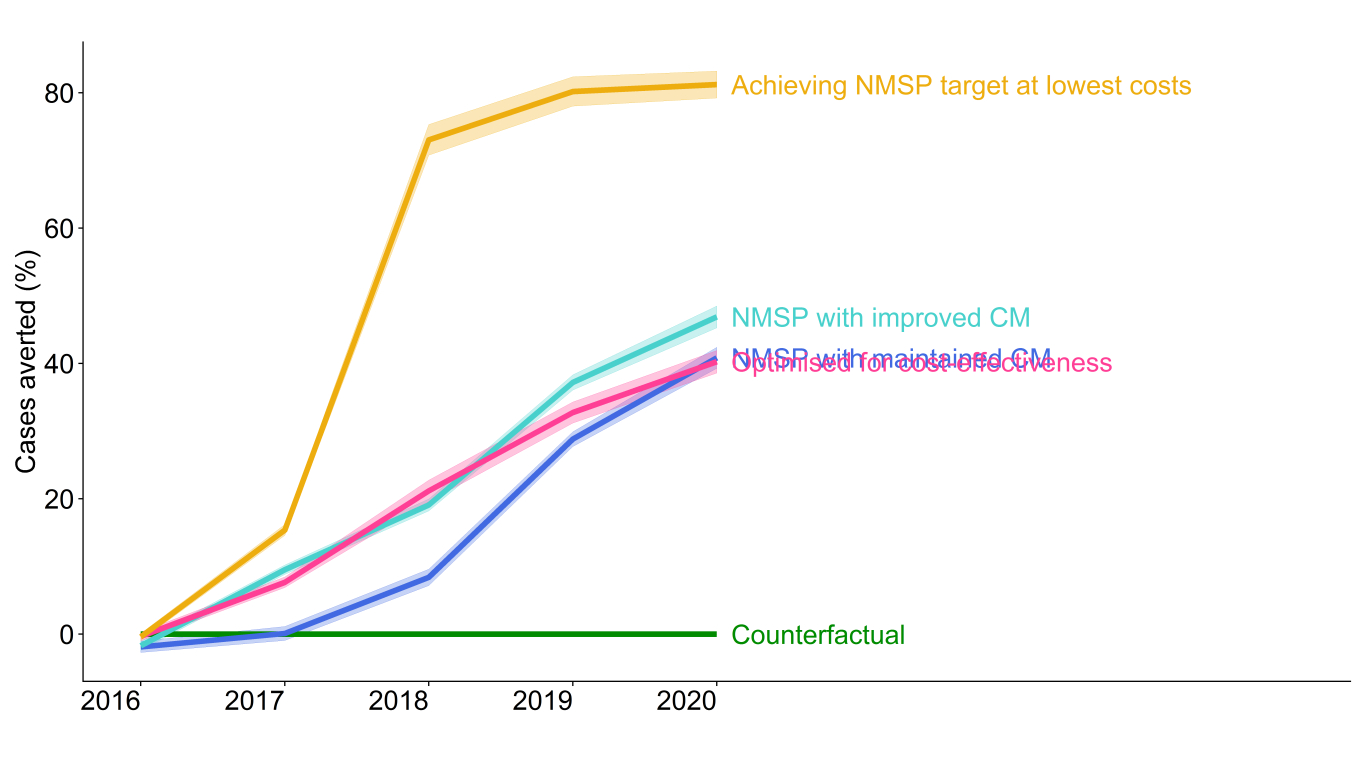
**Fig S4.8: Reduction in cases (%) over time per strategy.**

The solid line shows the aggregated mean and the shaded area the 95% confidence interval based on heterogeneity among councils. The NMSP with maintained case management as well as the NMSP with improved case management refers to strategy 1, the most cost-effective intervention package to strategy 2, and the intervention package achieving the NMSP target to strategy 3. The counterfactual shows the simulated scenario with maintained case management levels and the discontinuation of vector control interventions.

# Population weighed and unweighted mean prevalence

The presented predicted prevalences, aggregated at country level, were unweighted averages of the prevalence among the councils. The table below (Table 2) shows the averages weighted by the council population to obtain population-weighted mean predictions at national level. The two estimates, unweighted and weighted, were compared per strategy for three prevalence outcomes. The three prevalence outcomes were the predicted prevalence in 2020, the relative prevalence reduction compared to the baseline in 2016 (*Pf*PR_2020_ – *Pf*PR_2016_/*Pf*PR_2016_), and the relative reduction in prevalence compared to the counterfactual in 2020 (*Pf*PR_strategy_ – *Pf*PR_counterfactual_/*Pf*PR_counterfactual_). The weighted prevalence was very similar to the unweighted prevalence for all strategies for the prevalence, and the prevalence reduction compared to counterfactual, and showed higher differences in the prevalence reductions compared to the baseline in 2016 (Table S3.2).

**Table S4.2: Comparison of population-weighted and unweighted predicted mean prevalences per strategy at national level for 2020.**

| **Strategy** | ***Pf*PR_2to10_ (%)** | | ***Pf*PR_2to10_ reduction compared to**  **baseline in 2016 (%)** | | ***Pf*PR_2to10_ reduction compared to counterfactual (%)** | |
| --- | --- | --- | --- | --- | --- | --- |
|  | **unweighted** | **weighted** | **unweighted** | **weighted** | **unweighted** | **weighted** |
| Counterfactual | 21.5 | 20.8 | -65.8 | -60.2 | 0.0 | 0.0 |
| NMSP | 11.2 | 10.1 | 23.8 | 30.2 | 51.0 | 54.2 |
| NMSP with improved case management | 7.4 | 6.5 | 52.1 | 56.9 | 69.0 | 71.5 |
| Optimised for cost-effectiveness | 10.5 | 10.0 | 25.0 | 29.1 | 53.0 | 54.2 |
| Achieving NMSP target at lowest costs | 0.4 | 0.5 | 77.5 | 79.6 | 87.4 | 88.1 |

# References

1. Gosoniu L, Msengwa A, Lengeler C, Vounatsou P. Spatially Explicit Burden Estimates of Malaria in Tanzania: Bayesian Geostatistical Modeling of the Malaria Indicator Survey Data. PLoS ONE. 2012;7: e23966. doi:10.1371/journal.pone.0023966

2. Chacky F, Runge M, Rumisha SF, Machafuko P, Chaki P, Massaga JJ, et al. Nationwide school malaria parasitaemia survey in public primary schools, the United Republic of Tanzania. Malar J. 2018;17: 452. doi:10.1186/s12936-018-2601-1

3. Hagenlocher M, Castro MC. Mapping malaria risk and vulnerability in the United Republic of Tanzania: a spatial explicit model. Popul Health Metr. 2015;13: 2. doi:10.1186/s12963-015-0036-2

4. National Malaria Control Programme (Tanzania), WHO, Ifakara Health Institute, KEMRI-Wellcome Trust (Kenya). An epidemiological profile of malaria and its control in mainland Tanzania. Report funded by Roll Back Malaria and Department for International Development-UK; 2013 p. 152.

5. Massey NC, Garrod G, Wiebe A, Henry AJ, Huang Z, Moyes CL, et al. A global bionomic database for the dominant vectors of human malaria. Sci Data. 2016;3: 160014. doi:10.1038/sdata.2016.14

6. Huho BJ, Killeen GF, Ferguson HM, Tami A, Lengeler C, Charlwood JD, et al. Artemisinin-based combination therapy does not measurably reduce human infectiousness to vectors in a setting of intense malaria transmission. Malar J. 2012;11: 118. doi:10.1186/1475-2875-11-118

7. Curtis CF, Maxwell CA, Maxwell CA, Finch RJ, Finch RJ, Njunwa KJ. A comparison of use of a pyrethroid either for house spraying or for bednet treatment against malaria vectors. Trop Med Int Health. 1998;3: 619–631. doi:10.1046/j.1365-3156.1998.00281.x

8. Shiff CJ, Minjas JN, Hall T, Hunt RH, Lyimo S, Davis JR. Malaria infection potential of anopheline mosquitoes sampled by light trapping indoors in coastal Tanzanian villages. Med Vet Entomol. 1995;9: 256–262. doi:10.1111/j.1365-2915.1995.tb00131.x

9. Ijumba JN, Mosha FW, Lindsay SW. Malaria transmission risk variations derived from different agricultural practices in an irrigated area of northern Tanzania. Med Vet Entomol. 2002;16: 28–38.

10. Beier JC, Killeen GF, Githure JI. Short report: entomologic inoculation rates and Plasmodium falciparum malaria prevalence in Africa. Am J Trop Med Hyg. 1999;61: 109–113.

11. Penny MA, Maire N, Bever CA, Pemberton-Ross P, Briët OJT, Smith DL, et al. Distribution of malaria exposure in endemic countries in Africa considering country levels of effective treatment. Malar J. 2015;14: 384. doi:10.1186/s12936-015-0864-3

12. Charlwood JD, Smith T, Lyimo E, Kitua AY, Masanja H, Booth M, et al. Incidence of Plasmodium falciparum infection in infants in relation to exposure to sporozoite-infected anophelines. Am J Trop Med Hyg. 1998;59: 243–251.

13. Drakeley C, Schellenberg D, Kihonda J, Sousa CA, Arez AP, Lopes D, et al. An estimation of the entomological inoculation rate for Ifakara: a semi-urban area in a region of intense malaria transmission in Tanzania. Trop Med Int Health. 2003;8: 767–774. doi:10.1046/j.1365-3156.2003.01100.x

14. Killeen GF, Smith TA, Ferguson HM, Mshinda H, Abdulla S, Lengeler C, et al. Preventing Childhood Malaria in Africa by Protecting Adults from Mosquitoes with Insecticide-Treated Nets. PLoS Med. 2007;4. doi:10.1371/journal.pmed.0040229

15. Smith T, Charlwood JD, Kihonda J, Mwankusye S, Billingsley P, Meuwissen J, et al. Absence of seasonal variation in malaria parasitaemia in an area of intense seasonal transmission. Acta Trop. 1993;54: 55–72. doi:10.1016/0001-706X(93)90068-M

16. Freyvogel TA, Kihaule PM. Report on a limited anopheline survey at Ifakara, South-Eastern Tanzania. Acta Trop. 1968;25: 17–28.

17. Mboera LEG, Senkoro KP, Mayala BK, Rumisha SF, Rwegoshora RT, Mlozi MRS, et al. Spatio-temporal variation in malaria transmission intensity in five agro-ecosystems in Mvomero district, Tanzania. Geospatial Health. 2010;4: 167–178. doi:10.4081/gh.2010.198

18. Temu EA, Minjas JN, Coetzee M, Hunt RH, Shift CJ. The role of four anopheline species (Diptera: Culicidae) in malaria transmission in coastal Tanzania. Trans R Soc Trop Med Hyg. 1998;92: 152–158.

19. Bødker R, Akida J, Shayo D, Kisinza W, Msangeni HA, Pedersen EM, et al. Relationship Between Altitude and Intensity of Malaria Transmission in the Usambara Mountains, Tanzania. J Med Entomol. 2003;40: 706–717. doi:10.1603/0022-2585-40.5.706

20. Magesa SM, Wilkes TJ, Mnzava AEP, Njunwa KJ, Myamba J, Kivuyo MDP, et al. Trial of pyrethroid impregnated bednets in an area of Tanzania holoendemic for malaria Part 2. Effects on the malaria vector population. Acta Trop. 1991;49: 97–108. doi:10.1016/0001-706X(91)90057-Q

21. Mnzava AEP. Epidemiology and control of malaria transmission by residual house spraying with ddt and lambdacyhalothrin in two populations of the Anopheles gambiae complex in Tanga region. University of Basel. 1991.

22. Lin LI-K. A Concordance Correlation Coefficient to Evaluate Reproducibility. Biometrics. 1989;45: 255–268. doi:10.2307/2532051

1. EIR estimates were collated from literature and shared by Dr Fredros Okumu, Ifakara Health Institute and each reference was individually reviewed if accessible. [↑](#footnote-ref-1)
